# Efficacy of non-invasive brain stimulation combined with antidepressant medications for depression: a systematic review and meta-analysis of randomized controlled trials

**DOI:** 10.1186/s13643-024-02480-w

**Published:** 2024-03-20

**Authors:** Yuan Tao, Qian Liang, Fenghong Zhang, Shaofan Guo, Lingyun Fan, Fei Zhao

**Affiliations:** 1 School of Nursing, Gansu University of Chinese Medicine, Lanzhou, 73000 PR China; 2 Second Provincial Peoples Hospital of Gansu, Lanzhou, 73000 PR China; 3 Key Laboratory of Environmental Ecology and Population Health in Northwest Minority Areas, Medical College of Northwest Minzu University, Lanzhou, 730030 PR China

**Keywords:** NIBS, Depression, Transcranial magnetic stimulation, Transcranial direct current stimulation

## Abstract

**Background:**

Antidepressants, noninvasive brain stimulation (NIBS), and their combination are commonly used in routine clinical practice. Nevertheless, there is a continuous dispute regarding whether the effectiveness of NIBS in combination with antidepressants exceeds that of antidepressants alone. This meta-analysis aimed to evaluate the existing evidence and draw a definitive conclusion on this issue.

**Methods:**

We conducted a comprehensive search of five databases: Embase, PubMed, Web of Science, SinoMed, and the Cochrane Database of Randomized Controlled Trials. The search was conducted until October 6, 2023. The primary outcomes were the pre- and post-intervention depression and anxiety scores. Secondary outcomes included dropout rates, response rates, and certain levels of neurotransmitters [ 5-hydroxytryptamine (5-HT), dopamine (DA), and gamma-aminobutyric acid (GABA)] at the end of the intervention. Subgroup, meta-regression, and sensitivity analyses were performed to explore the sources of heterogeneity. The data were analysed using R 4.2.2.

**Results:**

We included 18 RCTs [1357 participants; 11 studies used repetitive transcranial magnetic stimulation (rTMS) and 7 studies used transcranial direct current stimulation (tDCS)]. The follow-up duration varied from two weeks to three months. Overall, whether in combination with rTMS or tDCS, antidepressants proved more effective in alleviating depressive symptoms compared to when used as monotherapy. However, this advantage was not evident during the follow-up period. (*p* > 0.05). And the combination's efficacy in improving anxiety was found to be lacking. Post-treatment serum levels of 5-HT, DA, and GABA were higher in the rTMS group were higher than antidepressant medication group (*p* < 0.05). Furthermore, subgroup analysis results indicated that only the rTMS + antidepressant medication treatment significantly improved remission and remission rates. The meta-regression results showed that the type of antidepressant and the sex of the participants had a significant association with the depression score.

**Conclusion:**

Combination treatment with NIBS was significantly more effective in improving depression symptoms than medication alone. rTMS combined with antidepressants appears to be more effective in improving response and remission rates. However, efficacy may be influenced by the type of medicine used in combination, and long-term efficacy data is lacking.

**Systematic review registration:**

PROSPERO CRD42023388259.

**Supplementary Information:**

The online version contains supplementary material available at 10.1186/s13643-024-02480-w.

## Background

Depression affects over 264 million people worldwide, making it one of the most prevalent mental health challenges [1]. Its recurring characteristics seriously affect the patient's daily functions and quality of life. According to a previous investigation conducted in the United States, the number of individuals suffering from depression increased dramatically during the COVID-19 pandemic, rising from 8.70% to 14.4% [2]. Despite considerable advancements in the pathophysiology and treatment of depression, a large number of patients do not respond to first-line treatment, approximately one-quarter do not respond to electroconvulsive treatment [3], and 30%-50% of patients do not respond to psychotherapy or medication [4]. Furthermore, approximately 10% of patients develop chronic diseases and suffer from severe cognitive impairment and psychosocial dysfunction [5]. Therefore, there is undoubtedly a need to explore more effective treatments for depression to reduce medical and economic costs.

Non-invasive brain stimulation (NIBS), including repetitive transcranial magnetic stimulation (rTMS) and transcranial direct current stimulation (tDCS), has been increasingly used to treat mental disorders because of its non-invasive nature, safety, and low economic burden [6–8]. tDCS is a non-invasive brain modulation technique that modulates cortical activity through the application of a weak direct current of 1–2 mA [9]. rTMS is applied to the prefrontal cortex to induce magnetic fields that modulate functional connectivity within and between the two cortical networks, thereby alleviating depressive symptoms [10].

While multiple meta-analyses have demonstrated the positive therapeutic effects of NIBS on various mental illnesses. For instance, Vergallito et al.'s [7] meta-analysis demonstrated the efficacy of rTMS treatment for anxiety disorders. Additionally, Hyde and colleagues [8] conducted a series of random-effects meta-analyses and indicated the positive effects of NIBS on anxiety, depression, and substance use disorders. However, these studies solely explored the clinical efficacy of NIBS as a standalone intervention. While each modality has traditionally been explored and developed as a monotherapy, it is typically used in combination. Moreover, the causes and mechanisms of depression are complex and diverse, and combination therapy is typically more comprehensive and targeted compared to singular interventions [11–13]. The number of clinical trials exploring the efficacy of NIBS combination therapy has increased significantly in recent years. There have been studies showing the combination of NIBS with psychosocial interventions exhibits significant therapeutic efficacy in alleviating moderate-to-severe depressive symptoms [11]. Although there have also been systematic reviews [12, 14] that evaluated the effect of the combination of NIBS with antidepressant intervention for major depressive disorder (MDD) and have shown it could accelerate the antidepressant effect of antidepressant medications. Nevertheless, these two systematic reviews encompassed a limited quantity of studies, and the overall quality of the studies was low, potentially resulting in inadequate reliability of the findings. Currently, the efficacy of NIBS in combination with antidepressants remains controversial. It was reported [15] that the outcome of active tDCS treatment was preferable to that of sham treatment in a study with 43 MDD patients. In contrast, Burkhardt’s study [16], a recent randomised controlled trial (RCT) published in *Lancet*, reached the opposite: there was no intergroup difference in the mean improvement in depression scores between active and sham stimulations.

These differences could be attributed to variances in the study methodology and NIBS parameter variables among RCTs [17], such as montages parameters (e.g., current density, stimulation frequency, and stimulus intensity) [18], individual differences in patients (e.g., age, symptom severity, and genetic factors) [19, 20], and types of medications combined [e.g., selective serotonin reuptake inhibitors (SSRIs) and serotonin-norepinephrine reuptake inhibitors (SNRI)] [16, 21]. However, it remains unclear how each of these factors and their interactions influence the efficacy of NIBS.

Based on all relevant published studies, we conducted a systematic review and meta-analysis to determine whether NIBS increases the efficacy of antidepressant medication. Our objectives were as follows: (1) to assess the clinical efficacy of two treatment strategies (tDCS combined with antidepressant medication and rTMS combined with antidepressant medication) and (2) to validate the robustness of the study conclusion through sensitivity analysis, bias risk assessment, meta regression and publication bias evaluation.

## Methods

This study's methodology adhered to the Cochrane Handbook for Systematic Reviews of Interventions [22]. The reporting of the study was conducted in accordance with the Preferred Reporting Items for Systematic Reviews and Meta-Analysis (PRISMA) guidelines [23]. This study was registered with PROSPERO (CRD42023388259).

### Searches

Two of the authors independently electronically searched PubMed, Embase, Web of Science, the Cochrane Database of Randomized Controlled Trials and SinoMed using the following words and phrases: (1)"Antidepressive Agents"[Mesh] OR "Antidepressive Agents, Second-Generation"[Mesh] OR "Antidepressive Agents, Tricyclic"[Mesh] OR (Antidepressive Agents OR "antidepress*" OR Selective serotonin reuptake inhibitors OR SSRIs OR Tricyclic antidepressant OR Serotonin and noradrenaline reuptake inhibitors OR Noradrenergic and specific serotonergic antidepressant OR Norepinephrine and dopamine reuptake inhibitors OR Monoamine oxidase inhibitors OR Vortioxetine OR Vilazodone OR Agomelatine OR Serotonin OR amitriptyline OR bupropion OR citalopram OR desvenlafaxine OR duloxetine OR escitalopram OR fluoxetine OR fluvoxamine OR levomilnacipran OR milnacipran OR mirtazapine OR nefazodone OR paroxetine OR reboxetine OR sertraline OR venlafaxine OR vilazodone OR vortioxetine)[Title/Abstract]; (2) "Transcranial Magnetic Stimulation"[Mesh] OR "Transcranial Direct Current Stimulation"[Mesh] OR (noninvasive Brain Stimulation OR NIBS OR Transcranial Magnetic Stimulation OR Transcranial Magnetic Stimulations OR Repetitive Transcranial Electrical Stimulation OR rTMS OR Cathodal Stimulation OR Transcranial Direct Current Stimulation OR Cathodal Stimulation tDCS OR Cathodal Stimulation tDCSs OR Transcranial Random Noise Stimulation OR Transcranial Alternating Current Stimulation OR Transcranial Electrical Stimulation OR Anodal Stimulation tDCS OR Anodal Stimulation tDCSs)[Title/Abstract];(3) "Depression"[Mesh] OR "Depressive Disorder"[Mesh] OR (Depress* OR "dysthymi* OR mood disorder* OR affective disorder* [Title/Abstract]). The ultimate search method was "(1) AND (2) AND (3)". Further complementary access to the relevant literature can be gained by reading the references incorporated into the literature. The deadline for the search was October 6, 2023. In addition, ClinicalTrials.gov (https://www.clinicaltrials.gov/) and Google Scholar (www.scholar. google.com.cn) were used as supplementary search. The specific search strategy is in (Additional file 1. Search strategies).

### Inclusion criteria

The population, intervention, comparison, outcome, and study designs (PICOS) framework [24] was the basis for the selection criteria. Studies meeting the following criteria were included in the meta-analysis:

Participants: adult individuals aged over 18 years who have been diagnosed with depression. The diagnosis of depression met DSM-IV, DSM-5, ICD-10 diagnostic criteria, or the depression disorder prevention guide.

Interventions: one of the NIBS techniques (rTMS or tDCS) was used in the interventions combined with antidepressant medications (the type and dose of medications were not restricted).

Comparison: the control group that received only medication did not receive the NIBS technique intervention.

Outcomes: the primary outcome was the depression scale score measured by the Hamilton Depression Rating Scale (HDRS), the Montgomery-Asberg Depression Rating Scale (MADRS), or the Beck Depression Inventory Rating Scale (BDI). The anxiety scale score measured was by State­Trait Anxiety Inventory (STAI). The secondary outcomes were clinical response rates, remission rates, drop-out rates, and changes in certain levels of neurotransmitters after intervention [i.e., dopamine (DA), gamma-aminobutyric acid (GABA), and 5-hydroxytryptamine (5-HT)]. The response rate was defined as a 50% or greater reduction in depression scores from baseline. The remission rate was defined by the criteria used in each trial (for example, an endpoint HDRS score ≤ 7 or MADRS score ≤ 10). If studies reported both the HDRS and MADRS scores, we analysed the scores from the scales used to define response and remission in their trials. The drop-out rate defined as the proportion of participants who prematurely discontinued their participation in the study for any cause.

Study designs: randomised controlled trials (RCTs), including parallel-group RCTs and crossover RCTs. We also considered quasi-randomised controlled trials (quasi-RCTs), in which the allocation was systematic but not random (e.g., based on hospitalisation number).

### Exclusion criteria

The exclusion criteria were as follows: (1) patients with other disorders (such as schizophreni, obsessive­compulsive disorder, substance use disorders, etc.); (2) non-simple depression patients (such as postpartum depression, bipolar disorder, geriatric depression, secondary depression, and vascular depression); (3) conference articles and case reports; (4) duplicate articles or duplicative datasets from the same trial; (5) articles lacking any of the primary outcomes; (6) articles not in Chinese or English.

### Study selection and data collection process

All of the search results were imported into the Zotero software, and duplicates were removed. Screening, eligibility determination, and inclusion in this systematic review followed the same procedure. Two reviewers each individually evaluated one article, and a third author resolved any differences. A data extraction form was prepared in accordance with the Cochrane Handbook for Systematic Reviews of Interventions. The same method previously mentioned was used to collect data. The main contents were extracted as follows: (1) first author's name and year of article publication; (2) clinical characteristics of the included studies (age, sample size, and types of depression); (3) treatment/control group information, including forms, doses, duration of antidepressant use, and treatment stimulation parameters of NIBS; (4) primary outcome; and (5) secondary outcome. All data are expressed as mean and standard deviation (SD). As this meta-analysis compared the values of the data change between the experimental and control groups before and after the intervention, the collected data had to be converted. The difference between the pre- and post-intervention assessed values was the change in value. If the change was negative, the estimated value after the intervention was lower than that before the intervention; otherwise, a positive value indicated an increase. The following formulas were used to determine the mean value of change: $\overline{{X }_{c}}=\overline{{X }_{a}}-\overline{{X }_{b}}$; and SD value change: ${S}_{c}=\sqrt{{S}_{a}^{2}+{S}_{b}^{2}-2\times corr\times {S}_{a}\times {S}_{b}}$, (corr = 0.50) [25]. If available, the intention-to-treat (ITT) or modified intention-to-treat (mITT) data were preferred to over data based only on completer.

### Quality assessment

We used the Risk of Bias Assessment Tool (ROB 2.0) [26] of the Cochrane Reviewers' Handbook 6.1 to assess the quality of the included studies. The five domains of ROB 2.0 are as follows: 1) the bias that is caused by the randomization method; 2) the bias that is caused by deviations from the interventions that were anticipated; 3) the bias that is caused by the absence of outcome data; 4) the bias that is generated by an evaluation of the outcome; and 5) the bias that is derived from the selection of the results that were presented. The risk of bias for each module was discussed and agreed upon by two researchers who composed each module. If a consensus judgement could not be reached, experts in evidence-based medicine, epidemiology, or health statistics were asked to assess it, and this conclusion was used to evaluate the total risk of bias in the article.

### Statistical analysis and synthesis of results

Odds ratios (OR) were used for dichotomous variables, and mean differences (MD) or standardised mean differences (SMD) were used for continuous variables. For each outcome, 95% confidence intervals (CI) were used as effect statistics. I^2^ was used to measure the degree of study outcome heterogeneity. We interpreted I^2^ following the Cochrane Handbook guidelines, considering the limitations of specific thresholds [27]: 0% to 40% may not be important; 30% to 60% may indicate moderate heterogeneity; 50% to 90% may indicate substantial heterogeneity; 75% to 100% is considered considerable heterogeneity. If there was significant statistical heterogeneity among the stuides, the source of heterogeneity was further analyzed, and a random-effects model was used for meta-analysis after excluding the effect of significant clinical heterogeneity. Significant clinical heterogeneity was addressed using methods such as subgroup, sensitivity, or only descriptive analyses. Test level ɑ = 0.05.

Analyses were conducted using the 'meta' and 'metafor' packages [28] for R software (version R × 64 4.2.2). All algorithms and scripts utilized within the software for conducting the statistical analysis are available in Additional file 3: algorithms and scripts. A funnel plot was used to assess publication bias, with asymmetry signifying potential publication bias, and the results were confirmed by Egger's test when bias was possible. We further visualised contour-enhanced funnel plots to assess whether the potential funnel asymmetry was likely to be due to a statistically significant publication bias. Sensitivity analysis was performed to test the robustness of the main and secondary outcomes.

## Results

### Selection of results

**Fig. 1:**
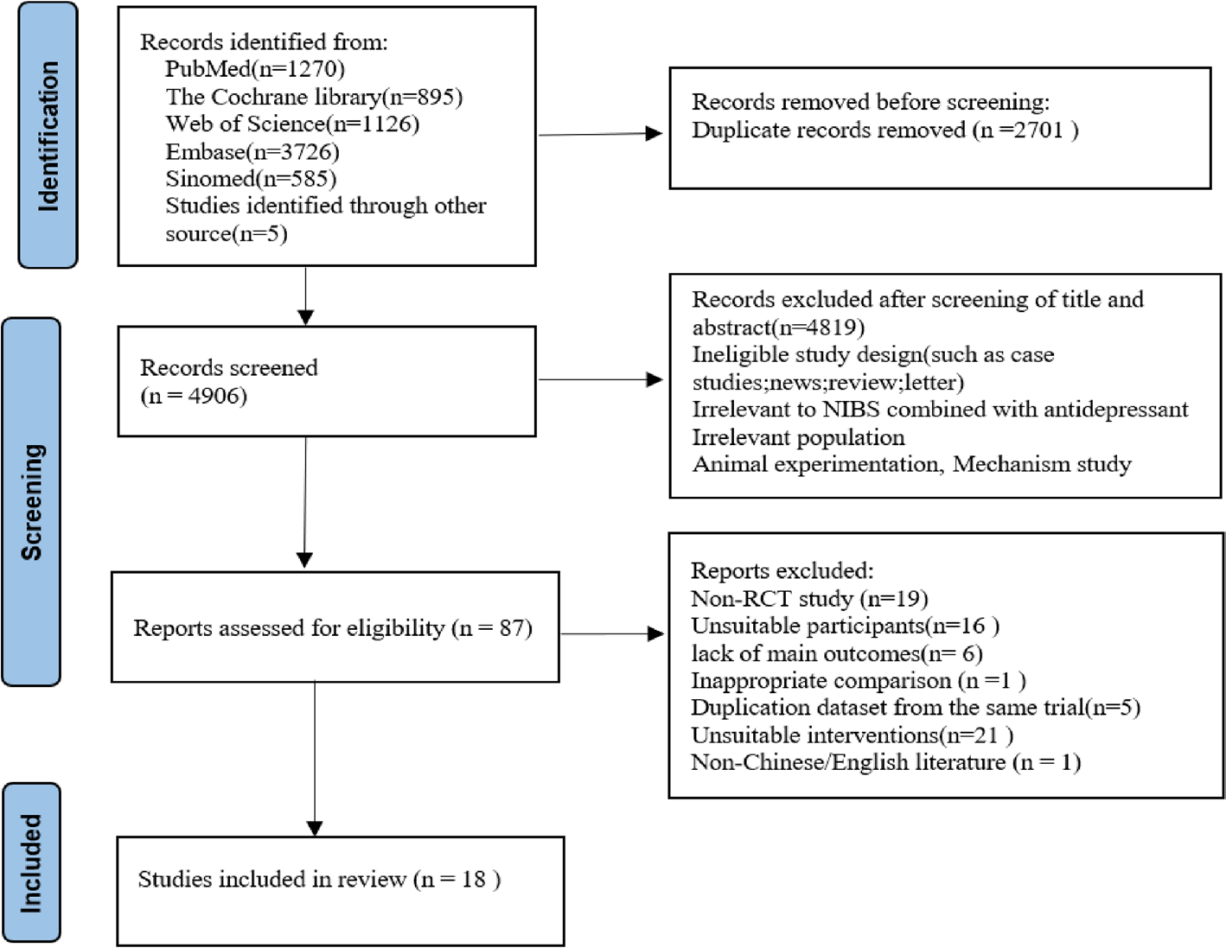
PRISMA flow chart for study selection

The PRISMA flow chart of the study selection is shown in Fig. 1. Based on the retrieval strategy, 7602 articles were obtained from the database, and five articles were supplemented by tracing references. Duplicates of 2701 articles were removed using Zotero software, and 4819 articles were excluded after screening titles and abstracts. Based on the eligibility criteria, 87 articles were potentially relevant to our systematic review. After the full-text evaluation, 69 articles were excluded (Additional file 2.list of the excluded full-text).

### Study characteristics

Overall, 18 RCTs with 1357 patients were included. The included studies were published between 2005 and 2023. The characteristics of the studies are presented in Table 1, and the characteristics of the NIBS specific parameters are presented in Table 2. These studies were conducted in Germany (*n* = 4); Brazil (*n* = 2); France (*n* = 1); China (*n* = 7); Russia (*n* = 1); Turkey (*n* = 1); India (*n* = 1); and Italy (*n* = 1). Seven studies used tDCS + medication, and eleven studies used rTMS + medication. The number of NIBS sessions varied, whereas the duration of the therapy ranged from a single application to 8 weeks. The most common treatment period was two weeks. The parameters of the neural stimulation settings varied considerably between studies. The frequencies employed ranged from 5 to 20 Hz. The intensity can be expressed as a Tesla (MT; 80–120%) motor threshold.

**Table 1:** Characteristics of the included studies

Authors, Years	Country	Multi-center	Funding	Registered	Type of Depression	Age (T)	Age (C)	Sample size		Intervention		Outcome Measure
								**T(n)**	**C(n)**	**T**	**C**	
**Burkhardt 2023** [16]	Germany	yes	yes	yes	MDD	40.2 ± 13.6	40.00 ± 13.30	77	73	tDCS + SSRI	sham tDCS + SSRI	①②③④⑤
**Kumari 2023** [15]	India	no	yes	yes	MDD	32.31 ± 11.57	29.08 ± 9.79	26	24	tDCS + escitalopram	sham tDCS + escitalopram	①②③④
**Li 2022** [29]	China	no	yes	yes	MDD	44.79 ± 15.25	43.61 ± 11.89	19	18	tDCS + escitalopram	sham tDCS + escitalopram	①
**Pavlova 2018a** [30]	Russia	no	NR	NR	mild and moderately depressed	36.00 ± 0.80	40.10 ± 2.20	22	20	tDCS + sertraline hydrochloride	sham tDCS + Sertraline hydrochloride	①②③④⑤
**Pavlova 2018b** [30]	Russia	no	NR	NR	mild and moderately depressed	37.0 ± 8.80	40.10 ± 12.20	27	20	tDCS + sertraline hydrochloride	sham tDCS + Sertraline hydrochloride	①②③④⑤
**Zhang 2020** [31]	China	no	yes	NR	MDD	43.94 ± 11.47	43.41 ± 10.76	35	35	tDCS + Vortioxetine	sham tDCS + fluoxetine	①②③
**Bennabi 2014** [32]	France	no	yes	yes	MDD	60.40 ± 12.00	59.90 ± 15.40	12	12	tDCS + sertraline hydrochloride	sham tDCS + sertraline hydrochloride	y①
**Brunoni 2013** [33]	Brazil	no	yes	yes	MDD	41.00 ± 13.00	41.00 ± 12.00	30	30	tDCS + sertraline hydrochloride	sham tDCS + sertraline hydrochloride	①②③④
**Pu 2023** [34]	China	no	yes	yes	mild and moderately depressed	35.24 ± 5.12	33.97 ± 4.74	50	50	high frequencyr TMS + agomelatine	sham rTMS + agomelatine	①④⑥
**Ma 2023** [35]	China	no	yes	NR	MDD	32.64 ± 9.48	31.28 ± 9.86	100	100	rTMS + duloxetine	duloxetine	①②⑥⑦⑧
**Akpinar 2022** [21]	Turkey	no	yes	yes	MDD	43.70 ± 14.20	45.60 ± 7.80	20	18	rTMS + Venlafaxine	sham rTMS + venlafaxine	①②
**Zhang 2019** [36]	China	no	yes	NR	moderate to severe depression	45.20 ± 8.70	43.70 ± 6.20	50	50	rTMS + duloxetine	sham rTMS + duloxetin	①⑥
**Wang 2017** [37]	China	no	yes	NR	MDD	28.82 ± 8.46	30.05 ± 9.47	22	21	rTMS + paroxetine	sham rTMS + paroxetine	①②③④⑦⑧
**Ullrich 2012** [38]	Germany	no	NR	NR	MDD	56.98 ± 10.20	54.18 ± 7.80	22	21	ultra-highfrequency rTMS + venlafaxine or mirtazapine	sham rTMS + venlafaxineor mirtazapine	①②③
**Huang 2012** [39]	China	no	yes	yes	MDD	32.77 ± 7.28	31.35 ± 7.39	28	28	rTMS + citalopram	sham rTMS + citalopram	①④
**Bretlau 2008** [40]	Germany	yes	no	yes	MDD	57.8 ± 10.0	53.10 ± 10.10	22	23	rTMS + citalopram	sham rTMS + citalopram	①③④
**Herwig 2007** [41]	Germany	no	yes	NR	mild and moderately depressed	50.00 ± 15.00	49.00 ± 13.00	62	65	rTMS + venfaraxine	sham rTMS + venfaraxin	①④
**Rossini 2005**[42]	Italy	no	NR	NR	MDD	48.40 ± 13.70	46.40 ± 12.10	50	49	rTMS + escitalopram	sham rTMS + escitalopram	①②③④
**Rumi 2005** [43]	Brazil	no	NR	NR	MDD	39.30 ± 12.80	38.90 ± 8.80	22	24	rTMS + amitriptyline	sham rtms + amitriptyline	①③

*NR* not report, *T* treatment group, *C* control group, *MDD* major depressive disorder, *rTMS* repetitive transcranial magnetic stimulation, *tDCS* transcranial direct currentstimulation

① depression score

② respond rate

③ remit rate

④ drop-out rate

⑤ state­trait anxiety inventory sore

⑥ 5-HT

⑦ NE

⑧ GABA

**Table 2:** Characteristics of NIBS treatment

Authors, Years	Type of NIBS	Cortical target	mA /Hz (%MT)	NIBS treatment protocol	N session (weeks)
**Burkhardt 2023** [16]	tDCS	Anode:LDLPFC Cathode: RDLPFC	2 mA	A current with an intensity 2 mA and a 30 s ramp­down phase delivered for 30 min	24 (6w)
**Kumari 2023** [15]	tDCS	Anode:LDLPFC Cathode: RDLPFC	2 mA	A current with an intensity of 2 mA and ramp time of 20 s was delivered for 20 min	10 (2w)
**Li 2022** [29]	tDCS	Anode:LDLPFC Cathode: RDLPFC	2 mA	A current with an intensity 2 mA and a 30 s ramp­down phase delivered for 30 min	10 (2w)
**Pavlova 2018a** [30]	tDCS	Anode:LDLPFC Cathode: RDLPFC	5 mA	A current with an intensity reduced 1 mA to 0.5 mA to compensate for smaller electrode size to keep current density constant delivered for 20 min	10 (2w)
**Pavlova 2018b** [30]	tDCS	Anode:LDLPFC Cathode: RDLPFC	5 mA	A current with an intensity reduced 1 mA to 0.5 mA to compensate for smaller electrode size to keep current density constant delivered for 30 min	10 (2w)
**Zhang 2020** [31]	tDCS	Anode:LDLPFC Cathode: RDLPFC	2 mA	A current with an intensity 2 mA delivered for 20 min	48 (8w)
**Bennabi 2014** [32]	tDCS	Anode:LDLPFC Cathode:contralateral supraorbital area	2 mA	A current with an intensity 2 mA delivered for 30 min	10 (1w)
**Brunoni 2013** [33]	tDCS	Anode:LDLPFC Cathode: RDLPFC	2 mA	A current with an intensity 2 mA delivered for 30 min	12 (6w)
**Pu 2023** [34]	rTMS	left DLPFC	10 Hz (120%)	Each train lastedasted 8 s with a 26s inter-train pause (800 pulses)	20 (8w)
**Ma 2023** [35]	rTMS	left DLPFC	20 Hz	Each train lastedasted 2 s with a 30s inter-train pause (800 pulses)	30 (6w)
**Akpinar 2022** [21]	rTMS	left DLPFC	10 Hz (110%)	Each train lastedasted 2.5 s with a 20s inter-train pause (2000 pulses)	10 (2w)
**Zhang 2019** [36]	rTMS	left DLPFC	10 Hz (80%)	NR	40 (8w)
**Wang 2017** [37]	rTMS	left DLPFC	10 Hz (80%)	Each train lasted 2 s with a 28-s inter-train pause (800 pulses)	20 (4w)
**Ullrich 2012** [38]	rTMS	left DLPFC	30 Hz (110%)	Each train lastedasted 3 s with a 57s inter-train pause (1800 pulses)	15 (3w)
**Huang 2012** [39]	rTMS	left DLPFC	10 Hz (90%)	Each train lastedasted 4 s with a 56s inter-train pause (800 pulses)	10 (2w)
**Bretlau 2008** [40]	rTMS	left DLPFC	10 Hz (90%)	Each train lastedasted 8 s with a 52s inter-train pause (1289 pulses)	15 (3w)
**Herwig 2007** [41]	rTMS	left DLPFC	10 Hz (110%)	Each train lastedasted 2s with a 8s inter-train pause (2000 pulses)	15 (3w)
**Rossini 2005** [42]	rTMS	left DLPFC	10 Hz (100%)	Each train lastedasted 2 s with a 28s inter-train pause (900 pulses)	10 (2w)
**Rumi 2005** [43]	rTMS	left DLPFC	10 Hz (120%)	Each train lasting 10 s, with 20-s interval (1250 pulses)	20 (4w)

*NR* Not report, *DLPFC* The dorsolateral prefrontal cortex, *MT* Motor threshold, *rTMS* Repetitive transcranial magnetic stimulation, *tDCS* transcranial direct currentstimulation

### Bias risk of included studies

**Fig. 2:**
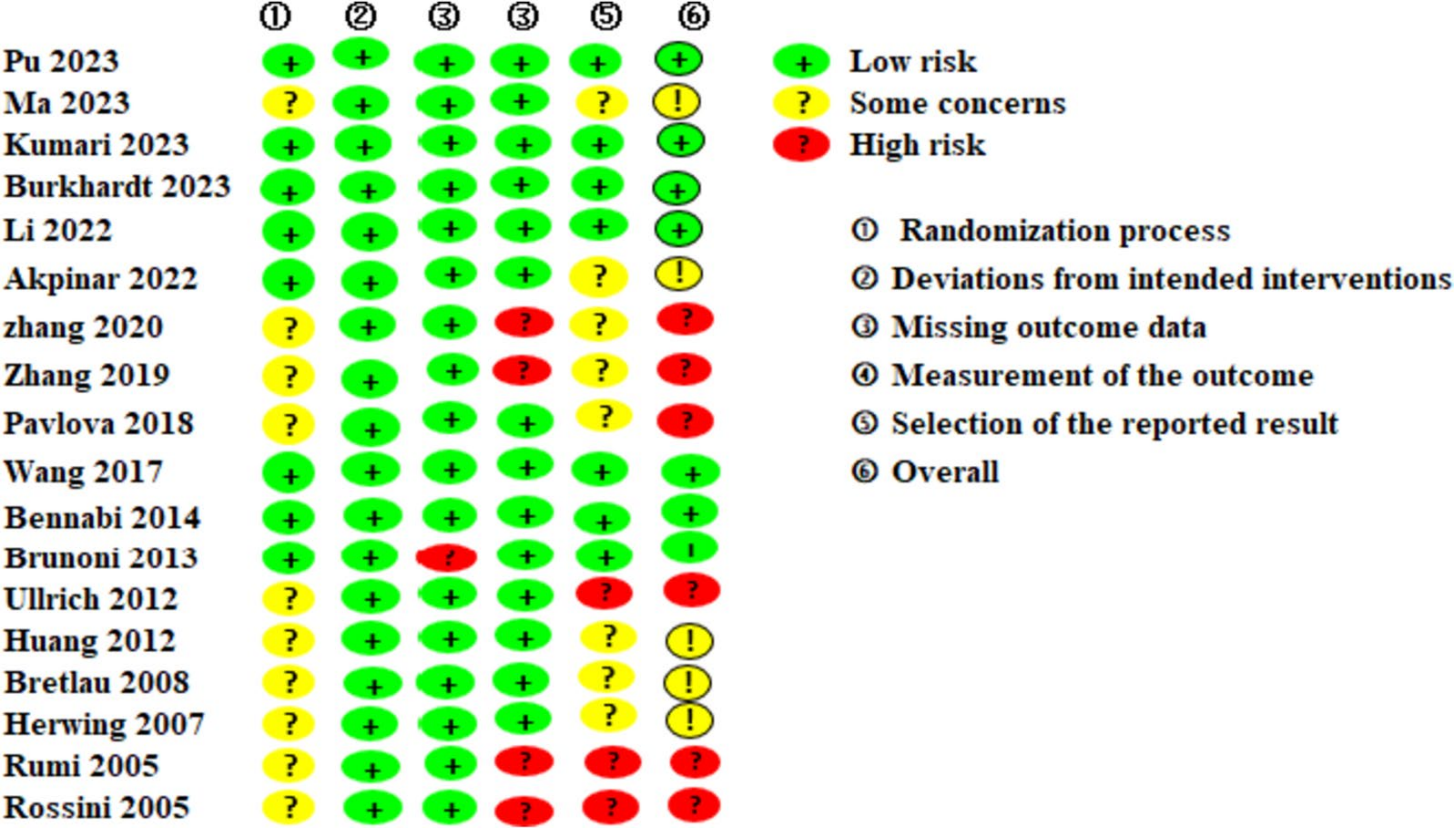
Results of bias risk evaluation of included studies

Of the studies, only eight [15, 16, 21, 29, 32–34, 37] provided clear descriptions of the methods employed for randomization and allocation concealment. Eleven studies [15, 16, 29, 30, 33, 34, 37, 39–42] reported loss of follow-up, with the rate of incomplete data ranging from 3.0% to 35%. The primary reasons for losses to follow-up typically included patient mortality, intolerable pain, and refusal to persist with the intervention. Seven studies [15, 16, 29, 32–34, 37] were at low risk of reporting bias, and the study protocol of these studies could be retrieved. A summary of this is shown in Fig. 2.

### Overall effects of NIBS treatments for depression

#### Depression score

**Fig. 3:**
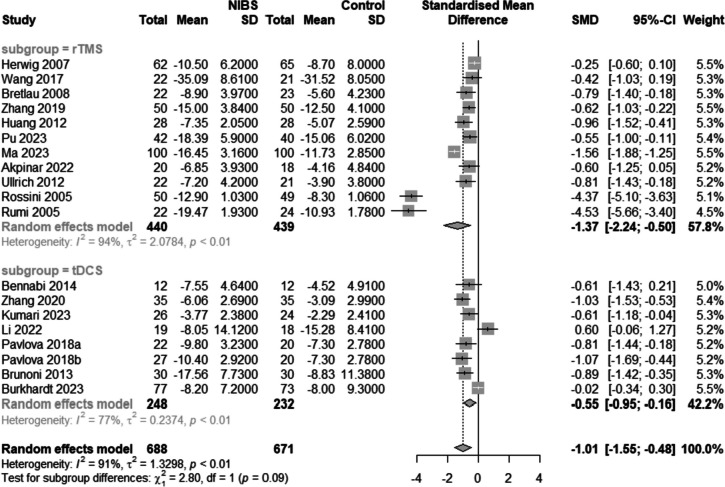
Forest plot of depression scores

All studies reported pre- and post-intervention depression scores (Fig. 3). Because there was significant heterogeneity among the included articles (I^2^ = 91.0%, *p* < 0.01), the results were pooled using a random effects model. The post-intervention reduction in depression levels was greater in the NIBS plus medication group than in the medication alone group [SMD = -1.01, 95%CI (-1.55,-0.48), I^2^ = 91.0%, *p* < 0.01]. Subgroup analyses were performed based on the type of intervention (i.e., tDCS + medication or rTMS + medication). The meta-analysis results showed that compared with medication alone, both rTMS combined with antidepressants [SMD = -1.37, 95%CI (-2.24,-0.50), I^2^ = 94.0%, *p* < 0.01] and tDCS combined with pharmacotherapy [SMD = -0.55, 95%CI (-0.95,-0.16), I^2^ = 77%, *p* = 0.01)] reduced depression scores, with statistically significant differences.

#### Anxiety score

**Fig. 4:**
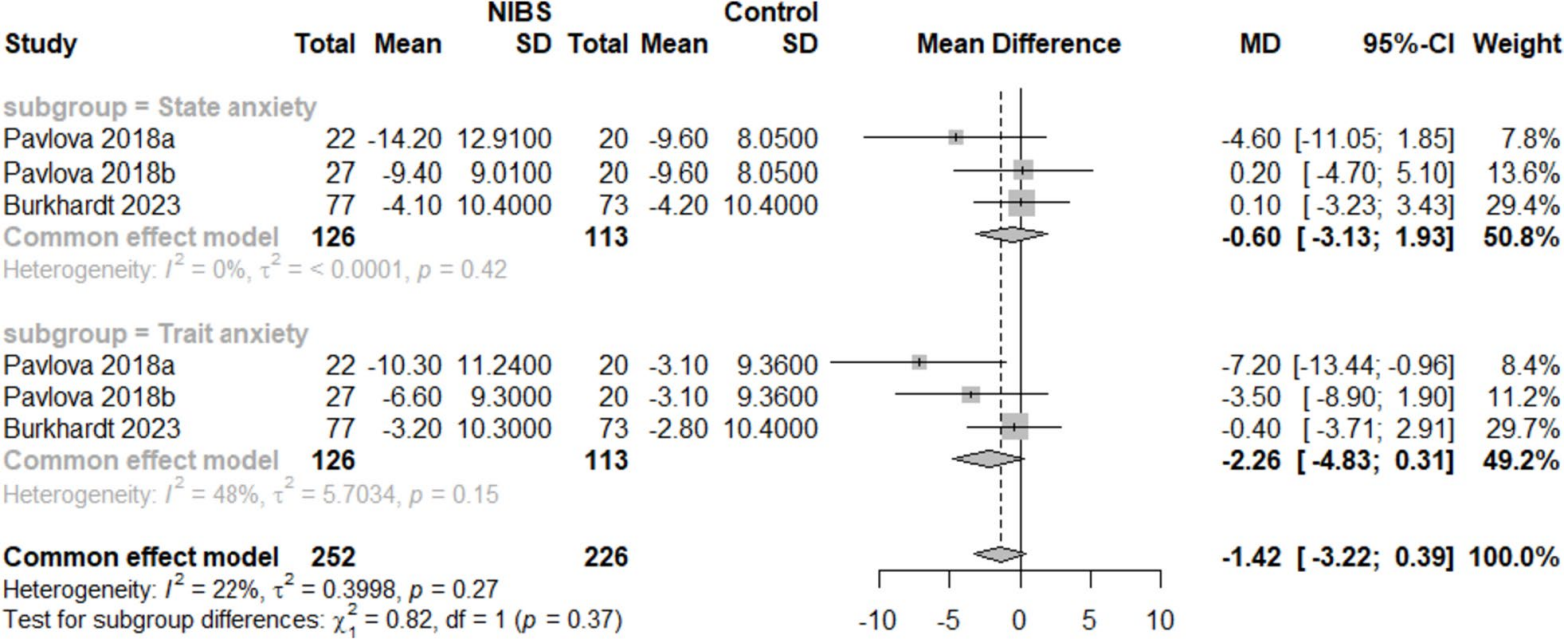
Forest plot of anxiety score

Two RCTs [16, 30] containing six useful datasets reported the effect of interventions on anxiety symptoms (Fig. 4). A fixed-effects model was adopted, considering I^2^ < 50%. There was no discernible difference in the anxiety symptoms between the intervention and control groups [MD = -1.42, 95% CI (-3.22, 0.39), I^2^ = 22%, *p* = 0.12].

#### The quantification of the neurotransmitters levels

**Fig. 5:**
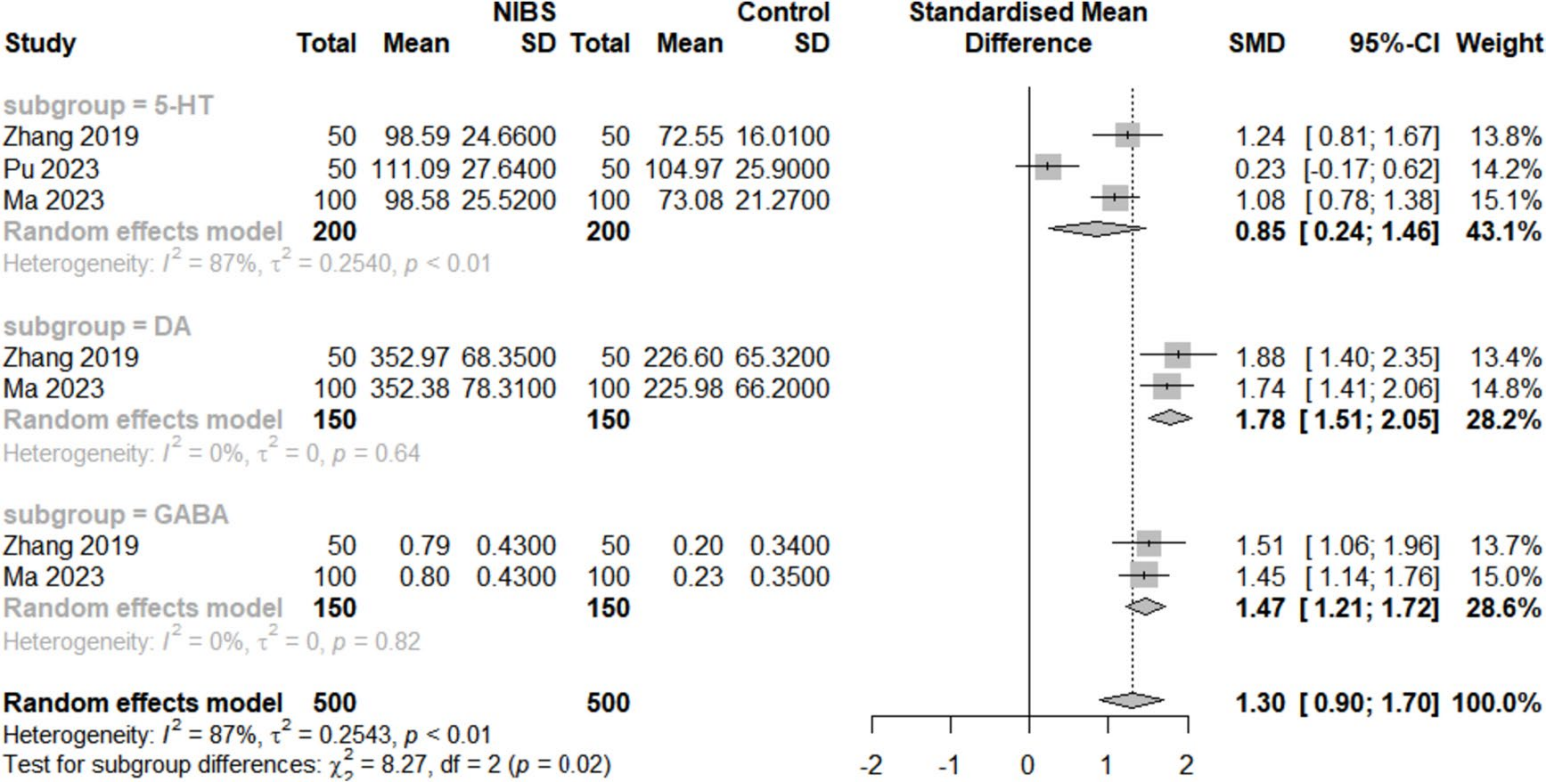
Forest plot of levels of neurotransmitters

The quantification of the neurotransmitter levels of 5-HT, DA, and GABA plays a regulatory role in the cognition and emotion of cells. Three studies [34–36] (all used rTMS) reported changes of 5-HT, and two studies [35, 36] reported changes of DA and GABA after intervention (Fig. 5), which showed a significant increase of the levels of 5-HT, DA, and GABA (SMD = 0.85, 95% CI (0.24, 1.64), I^2^ = 87%, *p* < 0.01), (SMD = 1.78, 95% CI (1.51, 2.05), I^2^ = 0%, *p* < 0.01), and (SMD = 1.47, 95% CI (1.21, 1.72), I^2^ = 0%, *p* < 0.01) separately.

#### Response rates of depression

**Fig. 6:**
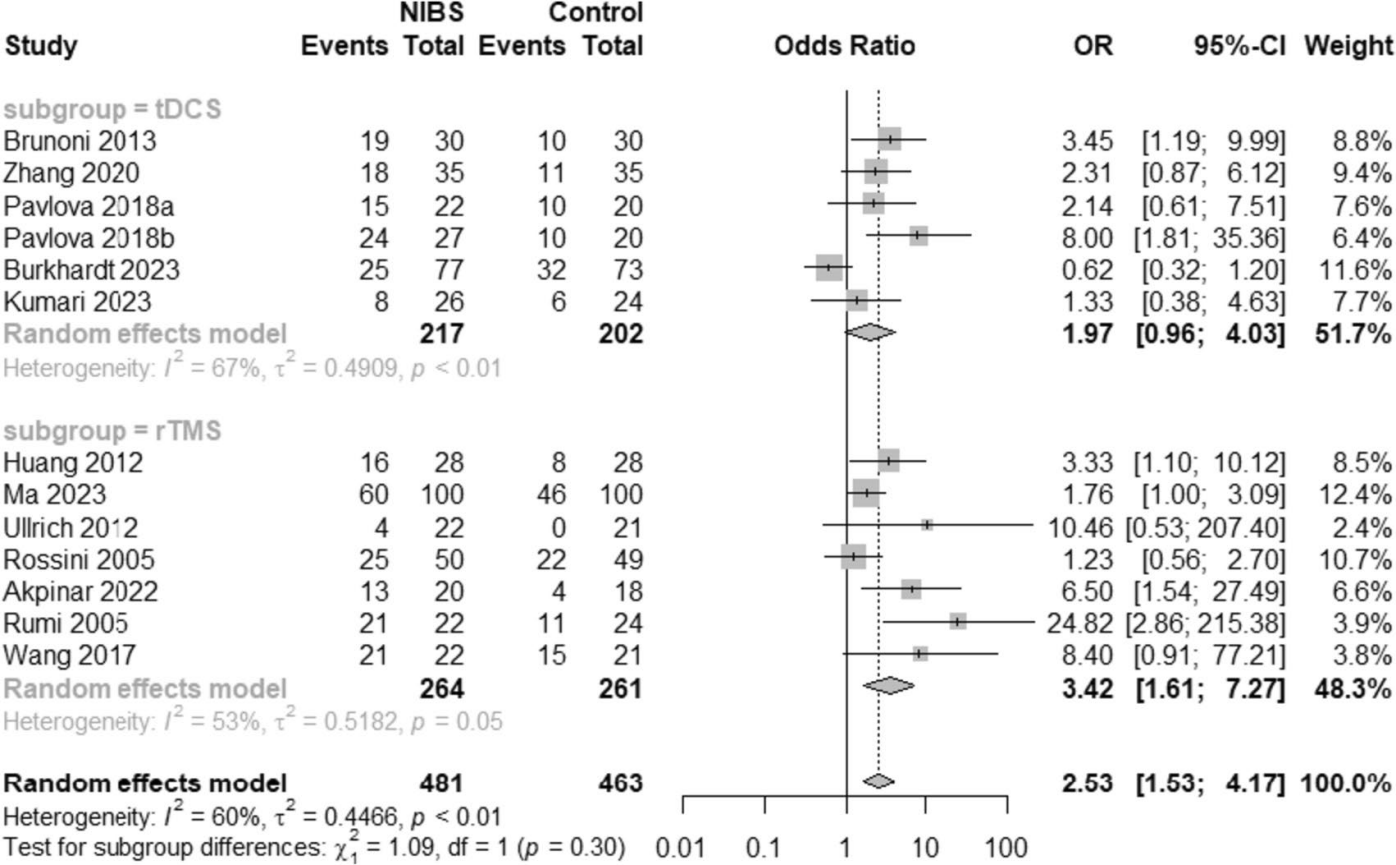
Forest plot of response rate

Twelve studies [15, 16, 21, 30, 31, 33, 35, 37, 39, 40, 42, 43] (seven rTMS and five tDCS) reported response rates (Fig. 6). Heterogeneity between these studies was significant (I^2^ = 60%, *p* < 0.01), therefore a random-effect model was used. Subgroup analysis results showed that antidepressants combined with rTMS improved the clinical response rate in patients with depression compared to controls [OR = 3.42, 95%CI (1.61, 7.27), I^2^ = 53%,* p* < 0.01]; however, no significant corresponding results were obtained for tDCS [1.97, 95%CI (0.96, 4.03), I^*2*^ = 67%, *p* > 0.05].

#### Remission rate of depression

**Fig. 7:**
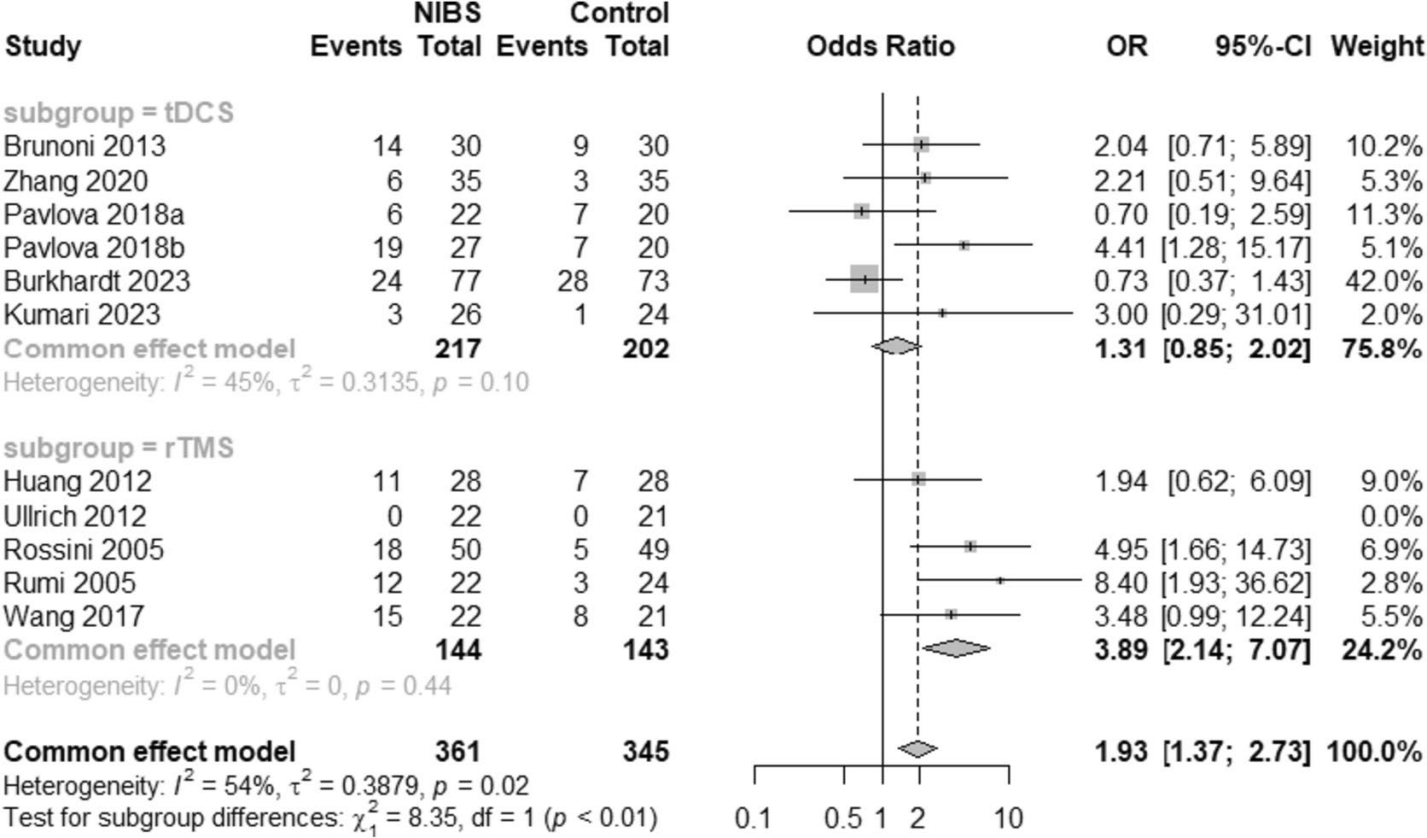
Forest plot of remission rate

Ten studies [15, 16, 30, 31, 33, 37–39, 42, 43] evaluated the response rates (Fig. 7). Significant heterogeneity was observed among the studies (I^2^ = 54%, *p* = 0.02). Among the five trials involving rTMS combined with medication therapy, substantial effect sizes were observed [OR = 3.89, 95% CI (2.14, 7.07), I^2^ = 0%, *p* < 0.01]. In contrast, the five trials exploring tDCS combined with medication therapy reported nonsignificant effect sizes [OR = 1.31, 95% CI (0.85, 2.02), I^2^ = 45%, *p* = 0.22].

#### Drop-out rate

**Fig. 8:**
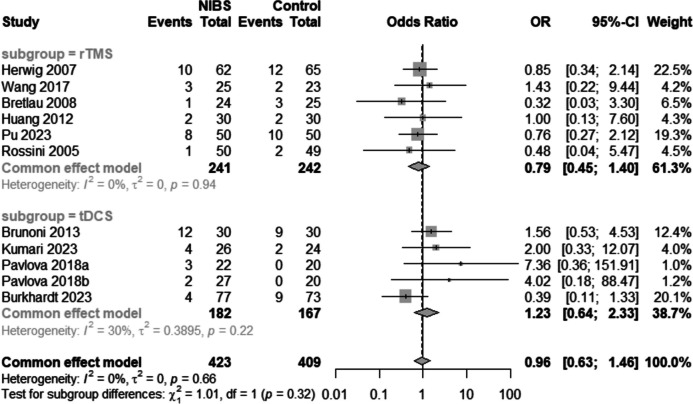
Forest plot of droup-out rate

Ten RCTs [15, 16, 30, 33, 34, 37, 39–42] reported dropout rates (Fig. 8). A fix-effect model was adapted because the heterogeneity was not significant (I^2^ = 0%, *p* = 0.53). The combined effect size [OR = 0.96, 95%CI (0.63,1.46), I^2^ = 0%, *p* = 0.53] indicated that the drop-out rates did not differ.

#### Follow-up time

**Fig. 9:**
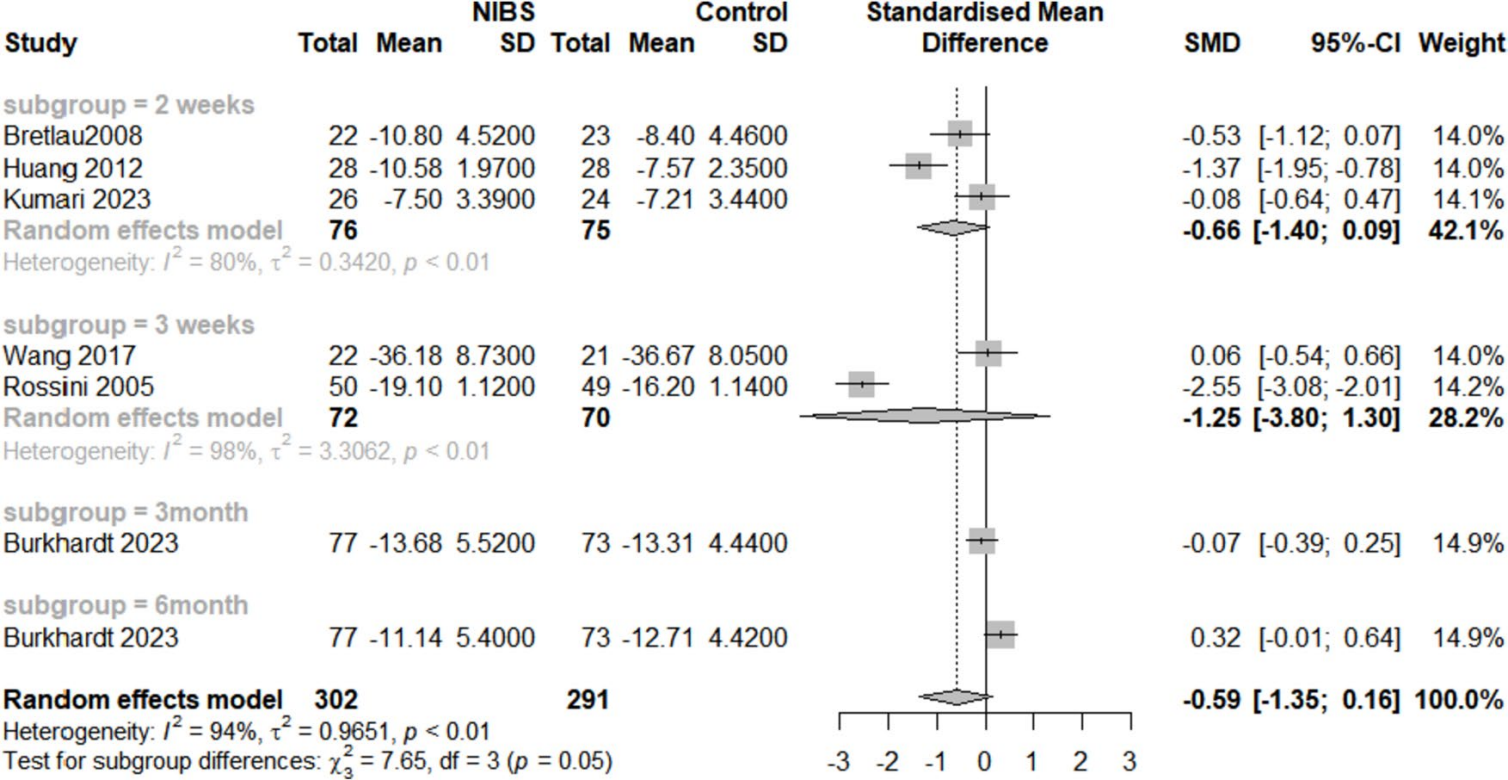
Forest plot of Follow-up time

As shown in Fig. 9, three RCTs [15, 36, 37] reported depression scores two weeks after intervention [SMD = -0.66, 95%CI (-1.40, 0.09), I^2^ = 80.0%, *p* = 0.05], and two RCTs [39, 41] reported depression scores three weeks after intervention [SMD = -1.25, 95%CI (-3.80, 1.30), I^2^ = 98.0%, *p* > 0.05]. One RCT [16] reported depression scores at three months and six months after intervention [SMD = -0.07, 95%CI (-0.39, 0.25), *p* > 0.05] and [SMD = -0.32, 95%CI (-0.01, 0.64), *p* > 0.05], respectively. These findings indicate that the combination has limited long-term efficacy in alleviating symptoms of depression.

#### Sensitivity analysis

Given the high heterogeneity of the included studies, we conducted sensitivity analyses of all results, and the results did not change after excluding each study (Additional file 4: Sensitivity Analysis). In summary, the outcomes obtained from the included trials were robust.

#### Publication bias

Publication bias was assessed using funnel plots and Egger's test (Additional file 5: Publication bias). Egger’s test of depression scores, remission rate, and dropout rate was not significant (*p* = 0.13, *p* = 0.16, and *p* = 0.24, respectively). However, Egger’s test of the response rate suggested potential publication bias (*p* < 0.01); five trials were missing after a "trim-and-fill" analysis. The funnel graph would have been more symmetrical if these five trials had been incorporated into the meta-analysis. The filled pooled estimate [OR = 0.81, 95% CI (1.51, 2.59), I^2^ = 59%, p < 0.01] based on the 18 trials was similar to the initial effect size. Biases in other outcomes were not considered due to the limited number of included studies.

### Meta-regression results

The meta-regression analysis indicated no significant association between the clinical parameters (such as the type of NIBS, the severity of depression, and the antidepressant class) and demographic factors (including the sample size, age, and percentage of females) with rates of remission or dropout. The details are shown in Additional file 6: Meta-regression analyses. A stepwise regression analysis was conducted to explore the relationship between the HAMD score and several independent variables (Table 3), revealing that the impact of the depression score was influenced by two factors: the use of tricyclic antidepressant medications (TCAs) and the sex of the participants (*p* = 0.01 and *p* = 0.03, respectively). Sample size demonstrated an influence on the response rate (*p* = 0.01). For other outcomes, the limited availability of studies precluded the application of meta-regression models.

**Table 3:** Meta-regression results

Variable	depression score		
	Coef (B)	95% CL	*p*
Clinical characteristics			
Type of NIBS	0.79	-0.26 to 1.84	0.14
Total session	0.00	-0.05 to 0.05	0.92
Severity of depression			
Major depression	-0.63	-2.55 to 1.27	0.51
Mild to moderate depressive	-0.15	-2.32 to 2.01	0.89
Moderate to severe depression	0.36	-2.67 to 3.38	0.82
Baseline score ^a^	-0.03	-0.12 to 0.06	0.50
Class of antidepressant			
SNRIs	-0.35	-2.60 to 189	0.76
SNRIs and NaSSAs	0.30	-2.51 to 312	0.83
SSRIs	-0.24	-2.36 to 1.89	0.83
SSRIs and SNRIs	-1.30	-3.78 to 1.18	0.31
TCAs	-3.97	-6.99 to -0.96	0.01*
Demographics			
Sample size	0.00	-1.01 to 0.01	0.51
Age	0.00	-0.06 to 0.06	0.97
Female rate	-4.45	-8.37 to -0.6	0.02*

*SNRIs* Serotonin-norepinephrine reuptake inhibitors, *NaSSAs* Noradrenergic and specific serotonergic antidepressants *TCAs* Tricyclic Anti-depressive Agents

^a^Baseline score was calculated by the weighted arithmetic mean of depression scores of NIBS and control groups. Each variable was analyzed separately in a meta-regression model, Coef (B) represents the regression coefficient of each linear regression, representing the slope of each model, 95% CI is the 95% confidence interval of the beta coefficient values

^*^p < 0.05

## Discussion

Antidepressant medication commonly takes at least 6–8 weeks to unfold its action entirely [44]. Delayed onset of treatment for depression is associated with a variety of difficulties, including cognitive impairment, decreased therapeutic compliance, patient and family suffering, economic impact, and increased rates of suicide [45–48]. A newly published RCT [16] has demonstrated the limited effectiveness of combination treatments, which may limit their potential in clinical practice. The implementation of combination therapy in the early stages of treatment has the potential to effectively manage depressive symptoms at the earliest feasible stage and shorten the onset of the action of antidepressants.

This meta-analysis, which included 18 RCTs with 1,375 participants, showed that both rTMS and tDCS combined with medications could effectively reduce depression in patients after treatment. However, there was no similar efficacy in reducing the anxiety symptoms. In addition, the long-term effectiveness of this combined treatment strategy seems to be insufficient because the difference in the treatment effect between the two groups was not statistically significant during the follow-up observation period. Meta-regression analysis showed that the current type of antidepressant and the sex of the participants were significantly associated with the depression score. Sample size was a factor that influenced the response rate.

Specifically, the results of the subgroup meta-analysis revealed that, compared with the medication group, rTMS treatment exhibited significantly higher efficacy in terms of response rate [OR = 3.42, 95%CI (1.61, 7.27), I^2^ = 53%, *p* < 0.01] and remission rate [OR = 3.89, 95%CI (2.14, 7.07), I^2^ = 0%, *p* < 0.01]. In contrast, outcomes involving tDCS yielded non-significant results for both the response rate [OR = 1.97, 95%CI (0.96, 4.03), I^2^ = 67%, *p* > 0.05] and the remission rate [OR = 1.31, 95%CI (0.85, 2.02), I^2^ = 45%, *p* = 0.22], which is consistent with a meta-analysis of depressed patients with traumatic brain injury performed by Tsai and Chang [49, 50]. Another meta-analysis reported that the tDCS group had a greater response rate than the sham tDCS group [OR = 2.70, 95%CI (1.33, 5.47), *p* < 0.01] [12], which may be due to the limited number of studies analyzed. In comparison to relevant published systematic reviews, our search covered an extended timeframe, employed a more refined search strategy, and incorporated a larger body of literature. To fortify the credibility of our findings, we conducted meta-regression, subgroup analyses, sensitivity analyses, and publication bias tests. Beyond assessing changes in depression scale scores before and after the intervention, our study delved into remission rates, clinical response rates, and alterations in specific neurotransmitter levels post-intervention, offering a more comprehensive understanding of the combined treatment.

The discrepancies in the efficacy of rTMS and tDCS may be attributed to differences in the fundamental principles and mechanisms of the two technologies. In rTMS, coil-generated magnetic fields on the skull create an electric current in the target brain area [51, 52]. In contrast, in tDCS, an electric current (usually 1–2 mA) flows directly to the patient's scalp via two or more electrodes [53]. Several studies [54–56] have suggested that the combination of tDCS and medication for depression may lead to negative efficacy or non-sham efficacy. A mixed experimental outcome showed that the efficacy of tDCS treatment depends on the type of medicine used [57]. The combination of tDCS with benzodiazepines, mood stabilisers (e.g., carbamazepine), antipsychotics, or other medications (e.g., L-dopa, rivastigmine, dextromethorphan, and flunarizine) may reduce the positive tDCS effects in both local and distant regions [56, 57]. Given the limited number of included studies, an in-depth analysis of the influence of various medications on the treatment results was not feasible. Nonetheless, further research is required to assess the effect of tDCS treatments on depression.

### Limitations

The primary limitations of the current study are as follows: First, we only examined mean treatment effects and were unable to investigate potentially crucial clinical and demographic variables of response to therapy at the individual level (e.g., age, sex, degree of severity of symptoms, or the period of illness). In randomised trials, patients are typically rigorously screened, and patients with bipolar disorder and other comorbidities are excluded. Psychological disorders are often highly comorbid, which may limit the applicability of the findings to these clinical subgroups; however, this was a methodological advantage to ensure the study's transitivity. In addition, our studies were highly heterogeneous, perhaps because the NIBS parameters and antidepressant medications used varied widely across the studies; however, the limited number of eligible studies prevented us from assessing how these potential factors affected heterogeneity. Moreover, five studies included in the analysis displayed an uncertain risk of bias, and in six of them, the overall quality of the included studies was not high.

Notwithstanding these limitations, the findings of this meta-analysis represent the most comprehensive evidence base currently available that may guide clinical guidelines and aid in a shared decision-making process involving patients, caregivers, and physicians when selecting the most appropriate treatment for adult patients with depressive disorder in their daily practice.

### Future directions

Future research should strive to expand the scope of meta-analyses by including both aggregate and individual patient data from clinical trials, thereby doing what is commonly referred to as an individual-patient data meta-analysis. Additional high-quality RCT trials with larger sample sizes are needed to further validate the efficacy of NIBS.

## Conclusion

In summary, this study demonstrated that NIBS combined with medication is more effective in treating depression. It significantly reduces depressive symptoms and enhances both remission and response rates among patients. This conclusion is valuable for clinical practice, as it implies that patients undergoing NIBS treatment concurrently with antidepressant medications can attain a more favourable treatment outcome. More high-quality, large-scale, multicenter RCTs are needed to further validate the effects of NIBS in combination with various antidepressants. Additionally, the findings of this study indicate the efficacy of combined therapy in adult patients with depression. Future research should extend its focus to other demographic groups with depression, including children, the elderly, and perinatal women.

### Supplementary Information

**Supplementary Materials file 1. **

**Supplementary Materials file 2. **

**Supplementary Materials file 3. **

**Supplementary Materials file 4. **

**Supplementary Materials file 5. **

**Supplementary Materials file 6. **

## Abbreviations

NIBS: Non-invasive Brain Stimulation
RCT: Randomized Controlled Trial
rTMS: Repetitive Transcranial Magnetic Stimulation
tDCS: Transcranial Direct Current Stimulation
SSRIs: Selective Serotonin Reuptake Inhibitors
DSM: The Diagnostic and Statistical Manual of Mental Disorders
ICD-10: International Classification of Diseases
HAMD: Hamilton Depression Rating Scale
MADRS: Montgomery-Asberg Depression Rating Scale
BDI: Beck Depression Inventory Rating Scale
STAI: State­Trait Anxiety Inventory
SMD: Standardized Mean Difference
MD: Mean difference
OR: Odds Ratio
MDD: Major Depressive Disorder
SNRI: Serotonin-Norepinephrine Reuptake Inhibitor
ITT: Intention-to-Treat
mITT: Modified Intention-to-Treat
DLPFC: Left Dorsolateral Prefrontal Cortex
5-HT: 5-Hydroxytryptamine
DA: Dopamine
GABA: Gamma Aminobutyric Acid
