# Supplementary material for: Efficacy of non-invasive brain stimulation combined with antidepressant medications for depression: a systematic review and meta-analysis of randomized controlled trials
Source: Syst Rev. 2024 Mar 20;13:92. doi: 10.1186/s13643-024-02480-w (PMC10953221; doi:10.1186/s13643-024-02480-w)
Supplement: Supplementary file 1 — Supplementary Materials file 1. [file 13643_2024_2480_MOESM1_ESM.docx]

Search strategy for Pubmed:

#1"Antidepressive Agents"[Mesh] OR "Antidepressive Agents, Second-Generation"[Mesh] OR "Antidepressive Agents, Tricyclic"[Mesh] 64,507

#2: "antidepress*"[Title/Abstract] OR Antidepressive Agents[Title/Abstract] OR Selective serotonin reuptake inhibitors[Title/Abstract] OR SSRIs[Title/Abstract] OR Tricyclic antidepressant[Title/Abstract] OR Serotonin[Title/Abstract] OR noradrenaline reuptake inhibitors[Title/Abstract] OR Norepinephrine[Title/Abstract] OR dopamine reuptake inhibitors[Title/Abstract] OR Monoamine oxidase inhibitors[Title/Abstract] OR amitriptyline[Title/Abstract] OR bupropion[Title/Abstract] OR citalopram[Title/Abstract] OR desvenlafaxine[Title/Abstract] OR duloxetine[Title/Abstract] OR escitalopram[Title/Abstract] OR fluoxetine[Title/Abstract] OR fluvoxamine[Title/Abstract] OR levomilnacipran[Title/Abstract] OR milnacipran[Title/Abstract] OR mirtazapine[Title/Abstract] OR nefazodone[Title/Abstract] OR paroxetine[Title/Abstract] OR reboxetine[Title/Abstract] OR sertraline[Title/Abstract] OR tianeptine[Title/Abstract] OR venlafaxine[Title/Abstract] OR vilazodone[Title/Abstract] OR vortioxetine[Title/Abstract]

242,340

#3 #1 OR #2 259,574

#4 "Transcranial Magnetic Stimulation"[Mesh] OR "Transcranial Direct Current Stimulation"[Mesh] 19,277

#5 noninvasive Brain Stimulation[Title/Abstract] OR NIBS[Title/Abstract] OR Transcranial Magnetic Stimulation[Title/Abstract] OR Transcranial Magnetic Stimulations[Title/Abstract] OR Repetitive Transcranial Electrical Stimulation[Title/Abstract] OR rTMS[Title/Abstract] OR Cathodal Stimulation[Title/Abstract] OR Transcranial Direct Current Stimulation[Title/Abstract] OR Cathodal Stimulation tDCS[Title/Abstract] OR Cathodal Stimulation tDCSs[Title/Abstract] OR Transcranial Random Noise Stimulation[Title/Abstract] OR Transcranial Alternating Current Stimulation[Title/Abstract] OR Transcranial Electrical Stimulation[Title/Abstract] OR Anodal Stimulation tDCS[Title/Abstract] OR Anodal Stimulation tDCSs[Title/Abstract] 27,091

#6 #4 OR#5 29302

#7 "Depression"[Mesh] OR "Depressive Disorder"[Mesh] 258,473

#8 "Depress*" OR "dysthymi*" OR "mood disorder*” OR "affective disorder*" 686,160

#9 #7 OR #8 686,309

#10 #3 AND #6 AND #9 1270

Search strategy for Cochrane library

#1 MeSH descriptor: [Antidepressive Agents] explode all trees7,028

#2 MeSH descriptor: [Antidepressive Agents, Second-Generation] explode all trees 1,511

#4 (Antidepressive Agents) :ti,ab,kw OR (antidepress*):ti,ab,kw OR (Selective serotonin reuptake inhibitors) :ti,ab,kw OR (SSRIs) :ti,ab,kw OR (Tricyclic antidepressant) :ti,ab,kw OR (Serotonin) :ti,ab,kw OR (noradrenaline reuptake inhibitors) :ti,ab,kw OR (dopamine reuptake inhibitors) :ti,ab,kw OR (Monoamine oxidase inhibitors) :ti,ab,kw OR (amitriptyline) :ti,ab,kw OR (bupropion) :ti,ab,kw OR (citalopram) :ti,ab,kw OR (desvenlafaxine) :ti,ab,kw OR (duloxetine) :ti,ab,kw OR (escitalopram) :ti,ab,kw OR (fluoxetine) :ti,ab,kw OR (fluvoxamine) :ti,ab,kw OR (levomilnacipran) :ti,ab,kw OR (milnacipran) :ti,ab,kw OR (mirtazapine) :ti,ab,kw OR (nefazodone) :ti,ab,kw OR (paroxetine) :ti,ab,kw OR (reboxetine) :ti,ab,kw OR (sertraline) :ti,ab,kw OR (tianeptine) :ti,ab,kw OR (venlafaxine) :ti,ab,kw OR (vilazodone) :ti,ab,kw OR (vortioxetine) :ti,ab,kw 44,649

#5 #1 OR #2 OR #3 OR #4 44,649

#6 MeSH descriptor:[Transcranial Magnetic Stimulation] explode all trees 2,358

#7 MeSH descriptor:[Transcranial Direct Current Stimulation] explode all trees 1,778

#8 (noninvasive Brain Stimulation) :ti,ab,kw OR (NIBS) :ti,ab,kw OR (Transcranial Magnetic Stimulation) :ti,ab,kw OR (Transcranial Magnetic Stimulations) :ti,ab,kw OR (Repetitive Transcranial Electrical Stimulation) :ti,ab,kw OR (rTMS) :ti,ab,kw OR (Cathodal Stimulation OR Transcranial Direct Current Stimulation) :ti,ab,kw OR (Cathodal Stimulation tDCS) :ti,ab,kw OR (Cathodal Stimulation tDCSs ) :ti,ab,kw OR (Transcranial Random Noise Stimulation) :ti,ab,kw OR (Transcranial Alternating Current Stimulation) :ti,ab,kw OR (Transcranial Electrical Stimulation) :ti,ab,kw OR (Anodal Stimulation tDCS0 :ti,ab,kw OR （Anodal Stimulation tDCSs) :ti,ab,kw 13,699

#9 #6 OR #7 OR #8 13,699

#10 MeSH descriptor:[Depression] explode all trees 18,679

#11 MeSH descriptor: [Depressive Disorder] explode all trees 15,284

#12 (Depress*):ti,ab,kw OR (dysthymi*):ti,ab,kw OR (mood disorder*):ti,ab,kw OR (affective disorder*):ti,ab,kw 136,801

#13 #10 OR #12 OR #11 136,818

#14 #5 AND #9 AND #13 895

Search strategy for Web of Science

((TS=(Antidepressive Agents OR "antidepress*" OR Selective serotonin reuptake inhibitors OR SSRIs OR Tricyclic antidepressant OR Serotonin OR noradrenaline reuptake inhibitors OR Norepinephrine OR dopamine reuptake inhibitors OR Monoamine oxidase inhibitors OR amitriptyline OR bupropion OR citalopram OR desvenlafaxine OR duloxetine OR escitalopram OR fluoxetine OR fluvoxamine OR levomilnacipran OR milnacipran OR mirtazapine OR nefazodone OR paroxetine OR reboxetine OR sertraline OR tianeptine OR venlafaxine OR vilazodone OR vortioxetine)) AND TS=(noninvasive Brain Stimulation OR NIBS OR Transcranial Magnetic Stimulation OR Transcranial Magnetic Stimulations OR Repetitive Transcranial Electrical stimulationof rTMS OR Cathodal Stimulation OR Transcranial Direct Current Stimulation OR Cathodal Stimulation tDCS OR Cathodal Stimulation tdcs OR Transcranial Random Noise Stimulation OR Transcranial Alternating Current Stimulation OR Transcranial Electrical Stimulation OR Anodal Stimulation tDCS OR Anodal Stimulation tdc)) AND TS=("Depress*" OR "dysthymi*" OR "mood disorder*" OR "affective disorder*") 1126

Search strategy for EMbase

#1 'antidepressant agent'/exp 592,688

#2 'antidepressant agent'/exp 592,688

#3 'antidepressive agents, tricyclic'/exp 123,477

#4 'antidepressive agents':ab,kw,ti 1,440

#5 'antidepress*':ab,kw,ti 118,997

#6 'selective serotonin reuptake inhibitors':ab,kw,ti 13,250

#7 'ssris':ab,kw,ti 12,977

#8 'tricyclic antidepressant':ab,kw,ti 4,767

#9 'serotonin':ab,kw,ti 133,411

#10 'noradrenaline reuptake inhibitors':ab,kw,ti 598

#11 'dopamine reuptake inhibitors':ab,kw,ti 147

#12 'monoamine oxidase inhibitors':ab,kw,ti 3,146

#13 'amitriptyline':ab,kw,ti 10,270

#14 'citalopram':ab,kw,ti 7,974

#15 'desvenlafaxine':ab,kw,ti 644

#16 'duloxetine':ab,kw,ti 5,273

#17 'escitalopram':ab,kw,ti 5,765

#18 'fluoxetine':ab,kw,ti 18,890

#19 'fluvoxamine':ab,kw,ti 3,996

#20 'levomilnacipran':ab,kw,ti 157

#21 'milnacipran':ab,kw,ti 1,143

#22 'mirtazapine':ab,kw,ti 4,090

#23 'nefazodone':ab,kw,ti 974

#24 'paroxetine':ab,kw,ti 8,608

#25 'reboxetine':ab,kw,ti 1,269

#26 'sertraline':ab,kw,ti 8,233

#27 'tianeptine':ab,kw,ti 859
#28 'venlafaxine':ab,kw,ti 7,320

#29 'vilazodone':ab,kw,ti 367

#30 'vortioxetine':ab,kw,ti 1,076

#31 #1 OR #2 OR #3 OR #4 OR #5 OR #6 OR #7 OR #8 OR #9 OR #10 OR #11 OR #12 OR #13 OR #14 OR #15 OR #16 OR #17 OR #18 OR #19 OR #20 OR #21 OR #22 OR #23 OR #24 OR #25 OR #26 OR #27 OR #28 OR #29 OR #30 695,443
#32 'transcranial magnetic stimulation'/exp 31,980

#33 'transcranial direct current stimulation'/exp 11,152

#34 'noninvasive brain stimulation':ab,ti,kw 1,491
#35 'nibs':ab,ti,kw 1,201

#36 'transcranial magnetic stimulation':ab,ti,kw 27,289

#37 'transcranial magnetic stimulations':ab,ti,kw 100

#38 'repetitive transcranial electrical stimulation':ab,ti,kw 20

#39 'rtms':ab,ti,kw 9,948

#40 'cathodal stimulation':ab,ti,kw 792

#41 'transcranial direct current stimulation':ab,ti,kw 9,460

#42 'cathodal stimulation tdcs':ab,ti,kw 2

#43 'cathodal stimulation tdcss':ab,ti,kw 0

#44 'transcranial alternating current stimulation':ab,ti,k 1,140

#45 'transcranial electrical stimulation':ab,ti,kw 1,203

#46 'anodal stimulation tdcs':ab,ti,kw 6

#47 'anodal stimulation tdcss':ab,ti,kw 0

#48 #32 OR #33 OR #34 OR #35 OR #36 OR #37 OR #38 OR #39 OR #40 OR #41 OR #42 OR #43 OR #44 OR #45 OR #46 OR #47 46,200

#49 'depression'/exp 650,371

#50 'depressive disorder'/exp 650,371

#51 'depressive disorder':ab,kw,ti 55,122

#52 'depress*':ab,kw,ti 780,228

#53 'dysthymi*':ab,kw,ti 4,486

#54 'mood disorder*':ab,kw,ti 35,554

#55 'affective disorder*':ab,kw,t 26,761

#56 #49 OR #50 OR #51 OR #52 OR #53 OR #54 OR #55 1,010,215

#57 #31 AND #48 AND #56 3726

Search strategy for Sinomed

#1 "抑郁症"[不加权:扩展] 168,257

#2 "抑郁症"[常用字段:智能] OR "抑郁综合征"[常用字段:智能] OR "抑郁"[常用字段:智能] OR "情感障碍"[常用字段:智能] OR "情绪病"[常用字段:智能] OR "情绪低落"[常用字段:智能] OR "郁病"[常用字段:智能] 749,431

#3 #1 OR #2 195,605

#4 "抗抑郁药"[不加权:扩展] OR "中枢神经系统抑制药"[不加权:扩展] OR "抗抑郁药"[不加权:扩展] OR "抗抑郁药, 三环"[不加权:扩展] OR "抗抑郁药, 三环"[不加权:扩展] OR "抗抑郁药, 第二代"[不加权:扩展] OR "抗抑郁药, 第二代"[不加权:扩展] OR "抗抑郁药, 第二代"[不加权:扩展] 66,459

#5 "抗抑郁药"[常用字段:智能] OR "抗抑郁剂"[常用字段:智能] OR "胸腺兴奋剂"[常用字段:智能] OR "选择性5-羟色胺再摄取抑制剂"[常用字段:智能] OR "SSRIs"[常用字段:智能] OR "三环抗抑郁剂"[常用字段:智能] OR "5-羟色胺"[常用字段:智能] OR "去甲肾上腺素再摄取抑制剂"[常用字段:智能] OR "多巴胺再摄取抑制剂"[常用字段:智能] 263,037

#6 "单胺氧化酶抑制剂"[常用字段:智能] OR "阿米替林"[常用字段:智能] OR "安非他酮"[常用字段:智能] OR "西酞普兰"[常用字段:智能] OR "文拉法辛"[常用字段:智能] OR "度洛西汀"[常用字段:智能] OR "氟西汀"[常用字段:智能] OR "氟伏沙明"[常用字段:智能] OR "艾司西酞普兰"[常用字段:智能] 700,007

#7 "米氮平"[常用字段:智能] OR "奈法唑酮"[常用字段:智能] OR "帕罗西汀"[常用字段:智能] OR "瑞波西汀"[常用字段:智能] OR "舍曲林"[常用字段:智能] OR "噻奈普汀"[常用字段:智能] OR "文拉法辛"[常用字段:智能] OR "维拉唑酮"[常用字段:智能] OR "伏噻西汀"[常用字段:智能] 28,542

#8 #4 OR #5 OR #6 OR #7

#9 ("经颅磁刺激"[不加权:扩展] OR "经颅磁刺激"[不加权:扩展] OR "经颅磁刺激"[不加权:扩展]) OR "经颅直流电刺激"[不加权:扩展] 5580

#10 "经颅磁刺激"[常用字段:智能] OR "经颅直流电刺激"[常用字段:智能] OR "经脑磁刺激"[常用字段:智能] OR "rTMS"[常用字段:智能] OR "tDCS"[常用字段:智能] OR "爆发式刺激"[常用字段:智能] OR "TBS"[常用字段:智能] OR "短阵快速脉冲经颅磁刺激"[常用字段:智能] OR "无创脑刺激"[常用字段:智能] 37,327

#11 #9 OR #10 37,327

#12 3 AND #8 AND #11 585
