# Supplementary material for: Efficacy of non-invasive brain stimulation combined with antidepressant medications for depression: a systematic review and meta-analysis of randomized controlled trials
Source: Syst Rev. 2024 Mar 20;13:92. doi: 10.1186/s13643-024-02480-w (PMC10953221; doi:10.1186/s13643-024-02480-w)
Supplement: Supplementary file 2 — Supplementary Materials file 2. [file 13643_2024_2480_MOESM2_ESM.docx]

**Included and excluded publications**

**S1. The list of excluded studies by full-text assessment and the reasons**

| Publication | Exclusion Reason | Note |
| --- | --- | --- |
| 1.Hausmann A, Pascual-Leone A, Kemmler G, Rupp CI, Lechner-Schoner T, Kramer-Reinstadler K, Walpoth M, Mechtcheriakov S, Conca A, Weiss EM. No deterioration of cognitive performance in an aggressive unilateral and bilateral antidepressant rTMS add-on trial. J Clin Psychiatry. 2004 Jun;65(6):772-82. doi: 10.4088/jcp.v65n0608. PMID: 15291654. | ② | Patients with bipolar disorder |
| 2.Padberg F, Zwanzger P, Thoma H, Kathmann N, Haag C, Greenberg BD, Hampel H, Möller HJ. Repetitive transcranial magnetic stimulation (rTMS) in pharmacotherapy-refractory major depression: comparative study of fast, slow and sham rTMS. Psychiatry Res. 1999 Nov 29;88(3):163-71. doi: 10.1016/s0165-1781(99)00092-x. PMID: 10622338. | ⑥ | Not all patients take antidepressants |
| 3.Little JT, Kimbrell TA, Wassermann EM, Grafman J, Figueras S, Dunn RT, Danielson A, Repella J, Huggins T, George MS, Post RM. Cognitive effects of 1- and 20-hertz repetitive transcranial magnetic stimulation in depression: preliminary report. Neuropsychiatry Neuropsychol Behav Neurol. 2000 Apr;13(2):119-24. PMID: 10780630. | ⑥ | Not all patients take antidepressants |
| 4.Loo CK, Mitchell PB, Croker VM, Malhi GS, Wen W, Gandevia SC, Sachdev PS. Double-blind controlled investigation of bilateral prefrontal transcranial magnetic stimulation for the treatment of resistant major depression. Psychol Med. 2003 Jan;33(1):33-40. doi: 10.1017/s0033291702006839. PMID: 12537034. | ⑥ | Not all patients take antidepressants |
| 5.Prasser J, Schecklmann M, Poeppl TB, Frank E, Kreuzer PM, Hajak G, Rupprecht R, Landgrebe M, Langguth B. Bilateral prefrontal rTMS and theta burst TMS as an add-on treatment for depression: a randomized placebo controlled trial. World J Biol Psychiatry. 2015 Jan;16(1):57-65. doi: 10.3109/15622975.2014.964768. Epub 2014 Nov 28. PMID: 25430687. | ⑥ | Not combined with antidepressants |
| 6.Jovicić M, Radovanović S, Marić NP, Kostić V. [Repetitive transcranial magnetic stimulation as an adjuvant method in the treatment of depression: preliminary results]. Srp Arh Celok Lek. 2014 May-Jun;142(5-6):280-5. Serbian. doi: 10.2298/sarh1406280j. PMID: 25033581. | ⑦ | Article in Serbian |
| 7.Meena, A. K. , Sharma, G. , Meena, S. , & Deshpande, S. N. . (2021). Transcranial direct current stimulation as an augmenting intervention in depression. | ① | non-RCT |
| 8. Martín JL. Adding repetitive transcranial magnetic stimulation to antidepressants does not improve response in people with depression. Evid Based Ment Health. 2008 Aug;11(3):78. doi: 10.1136/ebmh.11.3.78. PMID: 18669680. | ① | non-RCT |
| 9. Maneeton B, Maneeton N, Woottiluk P, Likhitsathian S. Repetitive Transcranial Magnetic Stimulation Combined with Antidepressants for the First Episode of Major Depressive Disorder. Curr Neuropharmacol. 2020;18(9):852-860. doi: 10.2174/1570159X18666200221113134. PMID: 32091338; PMCID: PMC7569318. | ① | non-RCT |
| 10. Jhanwar VG, Bishnoi RJ, Jhanwar MR. Utility of repetitive transcranial stimulation as an augmenting treatment method in treatment-resistant depression. Indian J Psychol Med. 2011 Jan;33(1):92-6. doi: 10.4103/0253-7176.85406. PMID: 22021964; PMCID: PMC3195166. | ① | Single arm study |
| 11. Jhanwar, Venu Gopal; Bishnoi, Ram Jeevan; Singh, Lakshman; Jhanwar, M. R.. Utility of repetitive transcranial magnetic stimulation as an augmenting treatment method in treatment-resistant depression. Indian Journal of Psychiatry 53(2):p 145-148, Apr–Jun 2011. \| DOI: 10.4103/0019-5545.82543 | ① | Single arm study |
| 12. Caulfield KA, Stern AP. Therapeutic High-Frequency Repetitive Transcranial Magnetic Stimulation Concurrently Improves Mood and Anxiety in Patients Using Benzodiazepines. Neuromodulation. 2020 Apr;23(3):380-383. doi: 10.1111/ner.13024. Epub 2019 Aug 1. PMID: 31368628. | ① | A retrospective study |
| 13. Brunoni AR, Ferrucci R, Bortolomasi M, Scelzo E, Boggio PS, Fregni F, Dell'Osso B, Giacopuzzi M, Altamura AC, Priori A. Interactions between transcranial direct current stimulation (tDCS) and pharmacological interventions in the Major Depressive Episode: findings from a naturalistic study. Eur Psychiatry. 2013 Aug;28(6):356-61. doi: 10.1016/j.eurpsy.2012.09.001. Epub 2012 Nov 23. PMID: 23182847. | ① | Single arm study |
| 14. Brunoni AR, Schestatsky P, Lotufo PA, Benseñor IM, Fregni F. Comparison of blinding effectiveness between sham tDCS and placebo sertraline in a 6-week major depression randomized clinical trial. Clin Neurophysiol. 2014 Feb;125(2):298-305. doi: 10.1016/j.clinph.2013.07.020. Epub 2013 Aug 30. PMID: 23994192. | ⑥ | Study blinding integrity and associated factors for transcranial direct current |
| 15. Chail A, Bhat PS, Singh H, Saini RK. Comparative efficacy of high-frequency repetitive transcranial magnetic stimulation to left dorsolateral prefrontal cortex as an augmentation strategy versus pharmacological augmentation in non-psychotic, unipolar, treatment-resistant depression: A randomized controlled trial. Ind Psychiatry J. 2023 Jan-Jun;32(1):93-99. doi: 10.4103/ipj.ipj_16_22. Epub 2022 Sep 14. PMID: 37274586; PMCID: PMC10236667. | ⑥ | The experimental group used only rTMS |
| 16. Chan HN, Alonzo A, Martin DM, Mitchell PB, Sachdev P, Loo CK. Augmenting transcranial direct current stimulation with (D)-cycloserine for depression: a pilot study. J ECT. 2013 Sep;29(3):196-200. doi: 10.1097/YCT.0b013e3182801b09. PMID: 23792778. | ① | Single arm study |
| 17. Hausmann A, Kemmler G, Walpoth M, Mechtcheriakov S, Kramer-Reinstadler K, Lechner T, Walch T, Deisenhammer EA, Kofler M, Rupp CI, Hinterhuber H, Conca A. No benefit derived from repetitive transcranial magnetic stimulation in depression: a prospective, single centre, randomised, double blind, sham controlled "add on" trial. J Neurol Neurosurg Psychiatry. 2004 Feb;75(2):320-2. PMID: 14742619; PMCID: PMC1738930. | ⑥ | The experimental group used only rTMS |
| 18. Kan RLD, Mak ADP, Chan SKW, Zhang BBB, Fong KNK, Kranz GS. Protocol for a prospective open-label clinical trial to investigate the utility of concurrent TBS/fNIRS for antidepressant treatment optimisation. BMJ Open. 2022 Feb 10;12(2):e053896. doi: 10.1136/bmjopen-2021-053896. PMID: 35144953; PMCID: PMC8845219. | ⑥ | The experimental group used only rTMS |
| 19. Moreno ML, Goerigk SA, Bertola L, Suemoto CK, Razza LB, Moffa AH, Veronezi BP, Tort L, Nogueira BS, Gattaz WF, Fraguas R, Padberg F, Lotufo PA, Benseñor IM, Brunoni AR. Cognitive changes after tDCS and escitalopram treatment in major depressive disorder: Results from the placebo-controlled ELECT-TDCS trial. J Affect Disord. 2020 Feb 15;263:344-352. doi: 10.1016/j.jad.2019.12.009. Epub 2019 Dec 5. PMID: 31969264. | ⑥ | tDCS vs escitalopram, |
| 20. Ray S, Nizamie SH, Akhtar S, Praharaj SK, Mishra BR, Zia-ul-Haq M. Efficacy of adjunctive high frequency repetitive transcranial magnetic stimulation of left prefrontal cortex in depression: a randomized sham controlled study. J Affect Disord. 2011 Jan;128(1-2):153-9. doi: 10.1016/j.jad.2010.06.027. Epub 2010 Jul 10. PMID: 20621361. | ⑥ | The experimental group used only rTMS |
| 21. Al-Sabbagh, A., Solly, A., Bending, N., Bowarshi, M.K., 2022. Esketamine following transcranial magnetic stimulation for treatment resistant depression. Brain Stimul. 15, 1327–1328. https://doi.org/10.1016/j.brs.2022.07.025 | ① | non-RCT study |
| 22. Best S , Pavel D G , Haustrup N .Long-Term Efficacy of Combination Therapy of Transcranial Magnetic Stimulation with Ketamine for Patients with Treatment-Resistant Depression.[J].CNS spectrums, 2021(2).DOI:10.1017/S1092852920002783. | ② | Patients with bipolar disorder |
| 23. Del Casale A, Rapinesi C, Kotzalidis GD, Sorice S, Padovano A, Gentile G, Angeletti G, Ferracuti S, Sani G, Pompili M, Simmaco M, Girardi P. Stable Remission of Multiple Chemical Sensitivity Syndrome and Major Depression With Citalopram and 1-Month Deep Transcranial Magnetic Stimulation: A Case Report. J ECT. 2017 Sep;33(3):e27-e29. doi: 10.1097/YCT.0000000000000420. PMID: 28570499. | ① | Case Reports |
| 24. Palm U, Goerigk S, Kirsch B, Bäumler L, Sarubin N, Hasan A, Brunoni AR, Padberg F. Treatment of major depression with a two-step tDCS protocol add-on to SSRI: Results from a naturalistic study. Brain Stimul. 2019 Jan-Feb;12(1):195-197. doi: 10.1016/j.brs.2018.10.003. Epub 2018 Oct 3. PMID: 30314900. | ① | Letter |
| 25. Poulet E, Brunelin J, Boeuve C, et al. Repetitive transcranial magnetic stimulation does not potentiate antidepressant treatment. Eur Psychiatry. 2004;19(6):382-383. doi:10.1016/j.eurpsy.2004.06.021 | ③ | lack of main outcomes |
| 26. Chen SJ, Chang CH, Tsai HC, Chen ST, Lin CCh. Superior antidepressant effect occurring 1 month after rTMS: add-on rTMS for subjects with medication-resistant depression. Neuropsychiatr Dis Treat. 2013;9:397-401. doi:10.2147/NDT.S40466 | ⑥ | The experimental group used only rTMS |
| 27. Kambeitz J, Goerigk S, Gattaz W, et al. Clinical patterns differentially predict response to transcranial direct current stimulation (tDCS) and escitalopram in major depression: A machine learning analysis of the ELECT-TDCS study. J Affect Disord. 2020;265:460-467. doi:10.1016/j.jad.2020.01.118 | ③ | lack of main outcomes |
| 28. Chen H, Hu X, Gao J, Han H, Wang X, Xue C. Early Effects of Repetitive Transcranial Magnetic Stimulation Combined With Sertraline in Adolescents With First-Episode Major Depressive Disorder. Front Psychiatry. 2022;13:853961. Published 2022 Jul 19. doi:10.3389/fpsyt.2022.853961 | ② | Adolescents |
| 29. Cole J, Selby B, Ismail Z, McGirr A. D-cycloserine normalizes long-term motor plasticity after transcranial magnetic intermittent theta-burst stimulation in major depressive disorder. Clin Neurophysiol. 2021;132(8):1770-1776. doi:10.1016/j.clinph.2021.04.002 | ③ | lack of main outcomes |
| 30.Robinson RG, Tenev V, Jorge RE. Citalopram for continuation therapy after repetitive transcranial magnetic stimulation in vascular depression. Am J Geriatr Psychiatry. 2009;17(8):682-687. doi:10.1097/JGP.0b013e3181a88423 | ② | Vascular Depression |
| 31. Yu F, He R. The effect of fluoxetine combined with repetitive transcranial magnetic stimulation on the psychological emotions and cognitive and neurological functions of acute post-stroke depression patients. Am J Transl Res. 2021;13(10):11883-11889. Published 2021 Oct 15. | ② | post-stroke depression |
| 32. Ustohal, L. , H. Přikrylová Kučerová, R. Přikryl, I. Stehnová, V. Hublová, & M. Mayerová, et al. (2014). Repetitive transcranial magnetic stimulation in the treatment of depressive disorder - a randomized, single-blind, antidepressants-controlled study. Ceska A Slovenska Neurologie A Neurochirurgie, 77(5), 602-607. | ⑥ | Antidepressants vs Repetitive transcranial magnetic stimulation |
| 33. Park S, Choi WJ, Kim S, et al. Effects of transcranial direct current stimulation using miniaturized devices vs sertraline for depression in Korea: A 6 week, multicenter, randomized, double blind, active-controlled study. J Psychiatr Res. 2020;127:42-47. doi:10.1016/j.jpsychires.2020.04.012 | ⑥ | Antidepressants vs transcranial direct current stimulation |
| 34. Mikellides G, Michael P, Psalta L, Schuhmann T, Sack AT. A Retrospective Naturalistic Study Comparing the Efficacy of Ketamine and Repetitive Transcranial Magnetic Stimulation for Treatment-Resistant Depression. Front Psychiatry. 2022;12:784830. Published 2022 Jan 13. doi:10.3389/fpsyt.2021.784830 | ⑥ | Antidepressants vs Repetitive transcranial magnetic stimulation |
| 35. Li S, Rong P, Wang Y, et al. Comparative Effectiveness of Transcutaneous Auricular Vagus Nerve Stimulation vs Citalopram for Major Depressive Disorder: A Randomized Trial. Neuromodulation. 2022;25(3):450-460. doi:10.1016/j.neurom.2021.10.021 | ⑥ | Unsuitable interventions |
| 36. Chen J, Xu P, Guo X, Zou T. Comparative Analysis of the Effects of Escitalopram, Pramipexole, and Transcranial Magnetic Stimulation on Depression in Patients With Parkinson Disease: An Open-Label Randomized Controlled Trial. Clin Neuropharmacol. 2022;45(4):84-88. doi:10.1097/WNF.0000000000000507 | ② | Depression in Patients With Parkinson Disease |
| 37. Schüle C, Zwanzger P, Baghai T, et al. Effects of antidepressant pharmacotherapy after repetitive transcranial magnetic stimulation in major depression: an open follow-up study. J Psychiatr Res. 2003;37(2):145-153. doi:10.1016/s0022-3956(02)00101-2 | ① | non-RCT study |
| 38. Tendler A, Gersner R, Roth Y, Zangen A. Alternate day dTMS combined with SSRIs for chronic treatment resistant depression: A prospective multicenter study. J Affect Disord. 2018 Nov;240:130-136. doi: 10.1016/j.jad.2018.07.058. Epub 2018 Jul 23. PMID: 30071416. | ① | non-RCT study |
| 39. Ngan STJ, Chan LK, Chan WC, Lam LCW, Li WK, Lim K, Or E, Pang PF, Poon TK, Wong MCM, Wu YKA, Cheng PWC. High-definition transcranial direct current stimulation (HD-tDCS) as augmentation therapy in late-life depression (LLD) with suboptimal response to treatment-a study protocol for a double-blinded randomized sham-controlled trial. Trials. 2022 Oct 28;23(1):914. doi: 10.1186/s13063-022-06855-z. PMID: 36307858; PMCID: PMC9617316. | ① | Experimental protocols |
| 40. Cole J, Sohn MN, Harris AD, Bray SL, Patten SB, McGirr A. Efficacy of Adjunctive D-Cycloserine to Intermittent Theta-Burst Stimulation for Major Depressive Disorder: A Randomized Clinical Trial. JAMA Psychiatry. 2022 Dec 1;79(12):1153-1161. doi: 10.1001/jamapsychiatry.2022.3255. Erratum in: JAMA Psychiatry. 2022 Dec 1;79(12):1241. PMID: 36223114; PMCID: PMC9557938. | ④ | Unsuitable controls(stimulation with a placebo) |
| 41. Park S, Choi WJ, Kim S, Kim B, Son SJ, Roh D, Kim WJ, Park JY. Effects of transcranial direct current stimulation using miniaturized devices vs sertraline for depression in Korea: A 6 week, multicenter, randomized, double blind, active-controlled study. J Psychiatr Res. 2020 Aug;127:42-47. doi: 10.1016/j.jpsychires.2020.04.012. Epub 2020 May 8. PMID: 32464489. | ⑥ | Unsuitable interventions Antidepressants vs transcranial direct current stimulation |
| 42. Brunoni AR, Carracedo A, Amigo OM, et al. Association of BDNF, HTR2A, TPH1, SLC6A4, and COMT polymorphisms with tDCS and escitalopram efficacy: ancillary analysis of a double-blind, placebo-controlled trial. Braz J Psychiatry. 2020;42(2):128-135. doi:10.1590/1516-4446-2019-0620 | ⑥ | Unsuitable interventions Antidepressants vs transcranial direct current stimulation |
| 43. Tendler A, Gersner R, Roth Y, Zangen A. Alternate day dTMS combined with SSRIs for chronic treatment resistant depression: A prospective multicenter study. J Affect Disord. 2018 Nov;240:130-136. doi: 10.1016/j.jad.2018.07.058. Epub 2018 Jul 23. PMID: 30071416. | ① | Single arm study |
| 44. Brunoni AR, Machado-Vieira R, Sampaio-Junior B, Vieira EL, Valiengo L, Benseñor IM, Lotufo PA, Carvalho AF, Cho HJ, Gattaz WF, Teixeira AL. Plasma levels of soluble TNF receptors 1 and 2 after Tdcs and sertraline treatment in major depression: Results from the SELECT-TDCS trial. J Affect Disord. 2015 Oct 1;185:209-13. Doi: 10.1016/j.jad.2015.07.006. Epub 2015 Jul 29. PMID: 26241865. | ③ | lack of main outcomes |
| 45. Brunoni AR, Machado-Vieira R, Zarate CA Jr, Vieira EL, Valiengo L, Benseñor IM, Lotufo PA, Gattaz WF, Teixeira AL. Assessment of non-BDNF neurotrophins and GDNF levels after depression treatment with sertraline and transcranial direct current stimulation in a factorial, randomized, sham-controlled trial (SELECT-TDCS): an exploratory analysis. Prog Neuropsychopharmacol Biol Psychiatry. 2015 Jan 2;56:91-6. doi: 10.1016/j.pnpbp.2014.08.009. Epub 2014 Aug 27. PMID: 25172025; PMCID: PMC4258544. | ③ | lack of main outcomes |
| 46. Conca A, Swoboda E, König P, Koppi S, Beraus W, Künz A, Fritzsche H, Weiss P. Clinical impacts of single transcranial magnetic stimulation (sTMS) as an add-on therapy in severely depressed patients under SSRI treatment. Hum Psychopharmacol. 2000 Aug;15(6):429-438. doi: 10.1002/1099-1077(200008)15:6<429::AID-HUP227>3.0.CO;2-3. PMID: 12404305. | ① | Single arm study |
| 47. Valiengo L, Benseñor IM, Goulart AC, de Oliveira JF, Zanao TA, Boggio PS, Lotufo PA, Fregni F, Brunoni AR. The sertraline versus electrical current therapy for treating depression clinical study (select-TDCS): results of the crossover and follow-up phases. Depress Anxiety. 2013 Jul;30(7):646-53. doi: 10.1002/da.22079. Epub 2013 Apr 26. PMID: 23625554. | ③ | lack of main outcomes |
| 48. Yu F, He R. The effect of fluoxetine combined with repetitive transcranial magnetic stimulation on the psychological emotions and cognitive and neurological functions of acute post-stroke depression patients. Am J Transl Res. 2021 Oct 15;13(10):11883-11889. PMID: 34786118; PMCID: PMC8581883. | ② | post-stroke depression |
| 49. Bonin Pinto C, Morales-Quezada L, de Toledo Piza PV, Zeng D, Saleh Vélez FG, Ferreira IS, Lucena PH, Duarte D, Lopes F, El-Hagrassy MM, Rizzo LV, Camargo EC, Lin DJ, Mazwi N, Wang QM, Black-Schaffer R, Fregni F. Combining Fluoxetine and rTMS in Poststroke Motor Recovery: A Placebo-Controlled Double-Blind Randomized Phase 2 Clinical Trial. Neurorehabil Neural Repair. 2019 Aug;33(8):643-655. doi: 10.1177/1545968319860483. Epub 2019 Jul 9. PMID: 31286828; PMCID: PMC6688938. | ② | post-stroke depression |
| 50 Wall CA, Croarkin PE, Sim LA, Husain MM, Janicak PG, Kozel FA, Emslie GJ, Dowd SM, Sampson SM. Adjunctive use of repetitive transcranial magnetic stimulation in depressed adolescents: a prospective, open pilot study. J Clin Psychiatry. 2011 Sep;72(9):1263-9. doi: 10.4088/JCP.11m07003. PMID: 21951987. | ② | depressed adolescents |
| 51. Pal E, Nagy F, Aschermann Z, Balazs E, Kovacs N. The impact of left prefrontal repetitive transcranial magnetic stimulation on depression in Parkinson's disease: a randomized, double-blind, placebo-controlled study. Mov Disord. 2010 Oct 30;25(14):2311-7. doi: 10.1002/mds.23270. PMID: 20740485. | ② | Depression in Patients With Parkinson Disease |
| 52. Janicak PG, Nahas Z, Lisanby SH, et al. Durability of clinical benefit with transcranial magnetic stimulation (TMS) in the treatment of pharmacoresistant major depression: assessment of relapse during a 6-month, multisite, open-label study. Brain Stimul. 2010;3(4):187-199. doi:10.1016/j.brs.2010.07.003 | ① | non-RCT study |
| 53. Boggio PS, Fregni F, Bermpohl F, et al. Effect of repetitive TMS and fluoxetine on cognitive function in patients with Parkinson's disease and concurrent depression. Mov Disord. 2005;20(9):1178-1184. doi:10.1002/mds.20508 | ② | post-stroke depression |
| 54. Su T P , Huang C C , Wei I H .Add-on rTMS for medication-resistant depression: a randomized, double-blind, sham-controlled trial in Chinese patients.[J].Journal of Clinical Psychiatry, 2005, 66(7):930.DOI:10.4088/JCP.v66n0718. | ② | Patients with bipolar disorder |
| 55 Schüle C, Zwanzger P, Baghai T, Mikhaiel P, Thoma H, Möller HJ, Rupprecht R, Padberg F. Effects of antidepressant pharmacotherapy after repetitive transcranial magnetic stimulation in major depression: an open follow-up study. J Psychiatr Res. 2003 Mar-Apr;37(2):145-53. doi: 10.1016/s0022-3956(02)00101-2. PMID: 12842168. | ① | Single arm study |
| 56. Wang Y, Liu X, Peng D, Wu Y, Su Y, Xu J, Ma X, Li Y, Shi J, Cheng X, Rong H, Fang Y. A Preliminary Study of Different Treatment Strategies for Anxious Depression. Neuropsychiatr Dis Treat. 2022 Jan 4;18:11-18. doi: 10.2147/NDT.S320091. PMID: 35018097; PMCID: PMC8742615. | ⑥ | Unsuitable interventions |
| 57. Brunoni AR, Júnior RF, Kemp AH, Lotufo PA, Benseñor IM, Fregni F. Differential improvement in depressive symptoms for tDCS alone and combined with pharmacotherapy: an exploratory analysis from the Sertraline vs. Electrical Current Therapy for Treating Depression Clinical Study. Int J Neuropsychopharmacol. 2014 Jan;17(1):53-61. doi: 10.1017/S1461145713001065. Epub 2013 Sep 23. PMID: 24060107. | ⑤ | duplicative dataset from the same trial |
| 58. Ustohal L ,H. Přikrylová Kučerová,R. Přikryl,et al.Repetitive Transcranial Magnetic Stimulation in the Treatment of Depressive Disorder - a Randomized, Single-blind, Antidepressants-controlled Study[J].Ceska A Slovenska Neurologie A Neurochirurgie, 2014, 77(5):602-607. | ⑥ | Unsuitable interventions |
| 59. Deppe M, Abdelnaim M, Hebel T, Kreuzer PM, Poeppl TB, Langguth B, Schecklmann M. Concomitant lorazepam use and antidepressive efficacy of repetitive transcranial magnetic stimulation in a naturalistic setting. Eur Arch Psychiatry Clin Neurosci. 2021 Feb;271(1):61-67. doi: 10.1007/s00406-020-01160-9. | ① | non-RCT study |
| 60. Qiao Y, Wang J, Zhu J, Cui H, Tang Y, Xu L, Tang X, Wei Y, Hui L, Li C, Jia Q, Zhu H, Wen H, Wang J, Zhang T. Antidepressant Effect of Adjunct Repetitive Transcranial Magnetic Stimulation in Inpatients 60 Years and Older. J ECT. 2020 Sep;36(3):216-221. doi: 10.1097/YCT.0000000000000648. PMID: 31972667. | ② | Some patients with bipolar disorder |
| 61. Brunoni AR, Sampaio-Junior B, Moffa AH, et al. The Escitalopram versus Electric Current Therapy for Treating Depression Clinical Study (ELECT-TDCS): rationale and study design of a non-inferiority, triple-arm, placebo-controlled clinical trial. *Sao Paulo Med J*. 2015;133(3):252-263. doi:10.1590/1516-3180.2014.00351712 | ⑤ | duplicative dataset from the same trial |
| 62 Brunoni AR, Machado-Vieira R, Zarate CA Jr, et al. BDNF plasma levels after antidepressant treatment with sertraline and transcranial direct current stimulation: results from a factorial, randomized, sham-controlled trial. *Eur Neuropsychopharmacol*. 2014;24(7):1144-1151. doi:10.1016/j.euroneuro.2014.03.006 | ⑤ | duplicative dataset from the same trial |
| 63. Mosimann UP, Schmitt W, Greenberg BD, et al. Repetitive transcranial magnetic stimulation: a putative add-on treatment for major depression in elderly patients. *Psychiatry Res*. 2004;126(2):123-133. doi:10.1016/j.psychres.2003.10.006 | ② | Some patients with bipolar disorder |
| 64. Wang Y, Liu X, Peng D, et al. A Preliminary Randomized Controlled Trial of Different Treatment Regimens for Melancholic Depression. *Neuropsychiatr Dis Treat*. 2021;17:2441-2449. Published 2021 Jul 23. doi:10.2147/NDT.S303938 | ⑥ | Unsuitable interventions (MECT/rTMS) |
| 65. Mogg A, Pluck G, Eranti SV, et al. A randomized controlled trial with 4-month follow-up of adjunctive repetitive transcranial magnetic stimulation of the left prefrontal cortex for depression. *Psychol Med*. 2008;38(3):323-333. doi:10.1017/S0033291707001663 | ⑥ | Unsuitable interventions(Not all patients took the medication) |
| 66. Höppner J, Schulz M, Irmisch G, Mau R, Schläfke D, Richter J. Antidepressant efficacy of two different rTMS procedures. High frequency over left versus low frequency over right prefrontal cortex compared with sham stimulation. Eur Arch Psychiatry Clin Neurosci. 2003;253(2):103-109. doi:10.1007/s00406-003-0416-7 | ② | Some patients with bipolar disorder |
| 67. Brunoni AR, Machado-Vieira R, Zarate CA, et al. Cytokines plasma levels during antidepressant treatment with sertraline and transcranial direct current stimulation (tDCS): results from a factorial, randomized, controlled trial. Psychopharmacology (Berl). 2014;231(7):1315-1323. doi:10.1007/s00213-013-3322-3 | ⑤ | duplicative dataset from the same trial |
| 68. Chen H, Hu X, Gao J, Han H, Wang X, Xue C. Early Effects of Repetitive Transcranial Magnetic Stimulation Combined With Sertraline in Adolescents With First-Episode Major Depressive Disorder. Front Psychiatry. 2022;13:853961. Published 2022 Jul 19. doi:10.3389/fpsyt.2022.853961 | ② | patients with adolescent |
| 69. Huang M-L, Luo B-Y. Repetitive transcranial magnetic stimulation in combination with citalopram in young patients with first-episode major depressive disorder: A double-blind, randomized, sham-controlled trial. Journal of ZheJiang University (Medical Science)2011;40:286–90. | ⑤ | duplicative dataset from the same trial |

Note:①Unsuitable study design(non-RCT study)18 ;②Unsuitable participants; ③lack of main outcomes; ④Unsuitable controls; ⑤duplicative dataset from the same trial; ⑥Unsuitable interventions;⑦Non-Chinese/English literature

**S2. The list of included studies(n=18)**

1. Ullrich H, Kranaster L, Sigges E, Andrich J, Sartorius A. Ultra-high-frequency left prefrontal transcranial magnetic stimulation as augmentation in severely ill patients with depression: a naturalistic sham-controlled, double-blind, randomized trial. Neuropsychobiology. 2012;66:141–8.

2. Rumi DO, Gattaz WF, Rigonatti SP, Rosa MA, Fregni F, Rosa MO, et al. Transcranial magnetic stimulation accelerates the antidepressant effect of amitriptyline in severe depression: A double-blind placebo-controlled study. BIOLOGICAL PSYCHIATRY. 2005;57:162–6.

3. Pavlova EL, Menshikova AA, Semenov RV, Bocharnikova EN, Gotovtseva GN, Druzhkova TA, et al. Transcranial direct current stimulation of 20- and 30-minutes combined with sertraline for the treatment of depression. Prog Neuro-Psychopharmacol Biol Psychiatry. 2018;82:31–8.

4. Burkhardt G, Kumpf U, Crispin A, Goerigk S, Andre E, Plewnia C, et al. Transcranial direct current stimulation as an additional treatment to selective serotonin reuptake inhibitors in adults with major depressive disorder in Germany (DepressionDC): a triple-blind, randomised, sham-controlled, multicentre trial. Lancet (london, england) [Internet]. 2023; Available from: https://www.cochranelibrary.com/central/doi/10.1002/central/CN-02580385/full

5. Brunoni A, Valiengo L, Baccaro A, Zanao T, De Oliveira J, Goulart A, et al. The sertraline vs electrical current therapy for treating depression clinical study. JAMA psychiatry. 2013;70:383‐391.

6. Huang M-L, Luo B-Y, Hu J-B, Wang S-S, Zhou W-H, Wei N, et al. Repetitive transcranial magnetic stimulation in combination with citalopram in young patients with first-episode major depressive disorder: A double-blind, randomized, sham-controlled trial. Aust New Zealand J Psychiatry. 2012;46:257–64.

7. Bretlau LG, Lunde M, Lindberg L, Unden M, Dissing S, Bech P. Repetitive transcranial magnetic stimulation (rTMS) in combination with escitalopram in patients with treatment-resistant major depression. A double-blind, randomised, sham-controlled trial. PHARMACOPSYCHIATRY. 2008;41:41–7.

8. Wang Y, Li N, Yang L, Song M, Shi L, Chen W, et al. Randomized controlled trial of repetitive transcranial magnetic stimulation combined with paroxetine for the treatment of patients with first-episode major depressive disorder. Psychiatry research. 2017;254:18‐23.

9. Bennabi D, Nicolier M, Monnin J, Tio G, Pazart L, Vandel P, et al. Pilot study of feasibility of the effect of treatment with tDCS in patients suffering from treatment-resistant depression treated with escitalopram. Clin Neurophysiol. 2015;126:1185–9.

10. Akpinar K, Kalkan Oğuzhanoğlu N, Toker Uğurlu T. Efficacy of transcranial magnetic stimulation in treatment-resistant depression. Turk J Med Sci. 2022;52:1344–54.

11. Zhang, FAN L, ZHOU Y, Li M. Efficacy of transcranial direct current stimulation combined with vothioxetine in the treatment of first-onset depression and its effect on neurofunction-related factors. Journal of Chinese Physician. 2021;23:1069-1073(Article in Chinese).

12. Pu Z, Hou Q, Yan H, Lin Y, Guo Z. Efficacy of repetitive transcranial magnetic stimulation and agomelatine on sleep quality and biomarkers of adult patients with mild to moderate depressive disorder. JOURNAL OF AFFECTIVE DISORDERS. 2023;323:55–61.

13. Zhang X. Effects of repeated transcranial magnetic stimulation combined with duloxetine on cognitive function and neurotransmitter levels in depression patients. Journal of International Psychiatry. 2019;46:1013-1015( Article in Chinese).

14. Rossini D, Magri L, Lucca A, Giordani S, Smeraldi E, Zanardi R. Does rTMS hasten the response to escitalopram, sertraline, or venlafaxine in patients with major depressive disorder? A double-blind, randomized, sham-controlled trial. J Clin Psychiatry. 2005;66:1569–75.

15. Li Z, Zhao X, Feng L, Zhao Y, Pan W, Liu Y, et al. Can Daytime Transcranial Direct Current Stimulation Treatment Change the Sleep Electroencephalogram Complexity of REM Sleep in Depressed Patients? A Double-Blinded, Randomized, Placebo-Controlled Trial. Front Psychiatry [Internet]. 2022;13. Available from: https://www.embase.com/search/results?subaction=viewrecord&id=L2017530499&from=export

16. Kumari B, Singh A, Kar SK, Tripathi A, Agarwal V. Bifrontal-transcranial direct current stimulation as an early augmentation strategy in major depressive disorder: A single-blind randomised controlled trial. Asian J Psychiatry [Internet]. 2023;86. Available from: https://www.embase.com/search/results?subaction=viewrecord&id=L2024830581&from=export

17. Herwig U, Fallgatter AJ, Höppner J, Eschweiler GW, Kron M, Hajak G, et al. Antidepressant effects of augmentative transcranial magnetic stimulation: Randomised multicentre trial. Br J Psychiatry. 2007;191:441–8.

18. Ma LJ,Yang ZQ,Zhang J, et al. lmpacts of duloxetine combined with repetitive transcranial magnetic stimulation onneurotransmitters and cognitive function in patients with severe depression Zhong Guo Kang FU. 2023;38:296–9. (Article in Chinese
