# Supplementary material for: Efficacy of non-invasive brain stimulation combined with antidepressant medications for depression: a systematic review and meta-analysis of randomized controlled trials
Source: Syst Rev. 2024 Mar 20;13:92. doi: 10.1186/s13643-024-02480-w (PMC10953221; doi:10.1186/s13643-024-02480-w)
Supplement: Supplementary file 3 — Supplementary Materials file 3. [file 13643_2024_2480_MOESM3_ESM.doc]

**algorithms and scripts.**

①depression

library(meta)

dat=read.csv("D:/depression.csv")

dat

study subgroup n1 mean1 sd1 n2 mean2 sd2

1 Herwig 2007 rTMS 62 -10.50 6.20 65 -8.70 8.00

2 Wang 2017 rTMS 22 -35.09 8.61 21 -31.52 8.05

3 Bretlau 2008 rTMS 22 -8.90 3.97 23 -5.60 4.23

4 Zhang 2019 rTMS 50 -15.00 3.84 50 -12.50 4.10

5 Huang 2012 rTMS 28 -7.35 2.05 28 -5.07 2.59

6 Pu 2023 rTMS 42 -18.39 5.90 40 -15.06 6.02

7 Ma 2023 rTMS 100 -16.45 3.16 100 -11.73 2.85

8 Akpinar 2022 rTMS 20 -6.85 3.93 18 -4.16 4.84

9 Ullrich 2012 rTMS 22 -7.20 4.20 21 -3.90 3.80

10 Rossini 2005 rTMS 50 -12.90 1.03 49 -8.30 1.06

11 Rumi 2005 rTMS 22 -19.47 1.93 24 -10.93 1.78

12 Bennabi 2014 tDCS 12 -7.55 4.64 12 -4.52 4.91

13 Zhang 2020 tDCS 35 -6.06 2.69 35 -3.09 2.99

14 Kumari 2023 tDCS 26 -3.77 2.38 24 -2.29 2.41

15 Li 2022 tDCS 19 -8.05 14.12 18 -15.28 8.41

16 Pavlova 2018a tDCS 22 -9.80 3.23 20 -7.30 2.78

17 Pavlova 2018b tDCS 27 -10.40 2.92 20 -7.30 2.78

18 Brunoni 2013 tDCS 30 -17.56 7.73 30 -8.83 11.38

19 Burkhardt 2023 tDCS 77 -8.20 7.20 73 -8.00 9.30

> meta1=metacont(n1,mean1,sd1,n2,mean2,sd2,data=dat,sm="SMD",studlab=study)

>meta1=metacont(n1,mean1,sd1,n2,mean2,sd2,data=dat,sm="SMD",studlab=study,comb.random=TRUE,comb.fixed=FALSE,label.e="NIBS",label.c="Control")

>meta1=metacont(n1,mean1,sd1,n2,mean2,sd2,data=dat,sm="SMD",byvar=subgroup,studlab=study,comb.random=TRUE,comb.fixed=FALSE,label.e="NIBS",label.c="Control")

> meta1

Number of studies combined: k = 19

Number of observations: o = 1359

SMD 95%-CI z p-value

Random effects model -1.0136 [-1.5502; -0.4770] -3.70 0.0002

Quantifying heterogeneity:

tau^2 = 1.3298 [0.7381; 3.3736]; tau = 1.1532 [0.8591; 1.8367]

I^2 = 91.5% [88.1%; 93.9%]; H = 3.42 [2.90; 4.04]

Test of heterogeneity:

Q d.f. p-value

210.77 18 < 0.0001

Results for subgroups (random effects model):

k SMD 95%-CI tau^2 tau Q I^2

subgroup = rTMS 11 -1.3673 [-2.2391; -0.4956] 2.0784 1.4417 161.36 93.8%

subgroup = tDCS 8 -0.5507 [-0.9461; -0.1553] 0.2374 0.4873 29.81 76.5%

Test for subgroup differences (random effects model):

Q d.f. p-value

Between groups 2.80 1 0.0945

Details on meta-analytical method:

- Inverse variance method

- Restricted maximum-likelihood estimator for tau^2

- Q-Profile method for confidence interval of tau^2 and tau

- Hedges' g (bias corrected standardised mean difference; using exact formulae)>

> forest(meta1)

> metainf(meta1)

Influential analysis (random effects model)

SMD 95%-CI p-value tau^2

Omitting Herwig 2007 -1.0593 [-1.6224; -0.4962] 0.0002 1.3876

Omitting Wang 2017 -1.0481 [-1.6135; -0.4828] 0.0003 1.4029

Omitting Bretlau 2008 -1.0280 [-1.5970; -0.4590] 0.0004 1.4227

Omitting Zhang 2019 -1.0380 [-1.6069; -0.4690] 0.0003 1.4189

Omitting Huang 2012 -1.0185 [-1.5884; -0.4486] 0.0005 1.4264

Omitting Pu 2023 -1.0418 [-1.6097; -0.4738] 0.0003 1.4143

Omitting Ma 2023 -0.9832 [-1.5495; -0.4168] 0.0007 1.4043

Omitting Akpinar 2022 -1.0381 [-1.6054; -0.4708] 0.0003 1.4143

Omitting Ullrich 2012 -1.0270 [-1.5960; -0.4580] 0.0004 1.4229

Omitting Rossini 2005 -0.8103 [-1.1960; -0.4246] < 0.0001 0.6076

Omitting Rumi 2005 -0.8435 [-1.2811; -0.4060] 0.0002 0.8144

Omitting Bennabi 2014 -1.0365 [-1.6027; -0.4702] 0.0003 1.4119

Omitting Zhang 2020 -1.0146 [-1.5848; -0.4444] 0.0005 1.4273

Omitting Kumari 2023 -1.0381 [-1.6060; -0.4702] 0.0003 1.4160

Omitting Li 2022 -1.1003 [-1.6358; -0.5648] < 0.0001 1.2504

Omitting Pavlova 2018a -1.0268 [-1.5957; -0.4578] 0.0004 1.4228

Omitting Pavlova 2018b -1.0128 [-1.5822; -0.4434] 0.0005 1.4247

Omitting Brunoni 2013 -1.0229 [-1.5928; -0.4529] 0.0004 1.4261

Omitting Burkhardt 2023 -1.0721 [-1.6295; -0.5146] 0.0002 1.3576

Pooled estimate -1.0136 [-1.5502; -0.4770] 0.0002 1.3298

tau I^2

Omitting Herwig 2007 1.1780 91.5%

Omitting Wang 2017 1.1845 91.9%

Omitting Bretlau 2008 1.1928 91.9%

Omitting Zhang 2019 1.1912 91.9%

Omitting Huang 2012 1.1943 91.9%

Omitting Pu 2023 1.1892 91.9%

Omitting Ma 2023 1.1850 90.8%

Omitting Akpinar 2022 1.1892 91.9%

Omitting Ullrich 2012 1.1928 91.9%

Omitting Rossini 2005 0.7795 85.5%

Omitting Rumi 2005 0.9025 89.9%

Omitting Bennabi 2014 1.1882 91.9%

Omitting Zhang 2020 1.1947 91.9%

Omitting Kumari 2023 1.1899 91.9%

Omitting Li 2022 1.1182 91.2%

Omitting Pavlova 2018a 1.1928 91.9%

Omitting Pavlova 2018b 1.1936 91.9%

Omitting Brunoni 2013 1.1942 91.9%

Omitting Burkhardt 2023 1.1652 90.8%

Pooled estimate 1.1532 91.5%

Details on meta-analytical method:

- Inverse variance method

- Restricted maximum-likelihood estimator for tau^2

metabias(meta1)

Linear regression test of funnel plot asymmetry

Test result: t = -1.58, df = 17, p-value = 0.1330

Sample estimates:

bias se.bias intercept se.intercept

-3.9029 2.4732 0.1608 0.6355

Details:

- multiplicative residual heterogeneity variance (tau^2 = 10.8143)

- predictor: standard error

- weight: inverse variance

- reference: Egger et al. (1997), BMJ

② anxiety

dat=read.csv("D:/anxiety.csv")

dat

study subgroup n1 mean1

1 Pavlova 2018a State anxiety 22 -14.2

2 Pavlova 2018b State anxiety 27 -9.4

3 Burkhardt 2023 State anxiety 77 -4.1

4 Pavlova 2018a Trait anxiety 22 -10.3

5 Pavlova 2018b Trait anxiety 27 -6.6

6 Burkhardt 2023 Trait anxiety 77 -3.2

sd1 n2 mean2 sd2

1 12.91 20 -9.6 8.05

2 9.01 20 -9.6 8.05

3 10.40 73 -4.2 10.40

4 11.24 20 -3.1 9.36

5 9.30 20 -3.1 9.36

6 10.30 73 -2.8 10.40

meta1=metacont(n1,mean1,sd1,n2,mean2,sd2,data=dat,sm="MD",studlab=study)

meta1

MD 95%-CI z p-value

Common effect model -1.4171 [-3.2219; 0.3877] -1.54 0.1238

Random effects model -1.4892 [-3.3830; 0.4047] -1.54 0.1233

Quantifying heterogeneity:

tau^2 = 0.3998 [0.0000; 47.6813]; tau = 0.6323 [0.0000; 6.9052]

I^2 = 21.8% [0.0%; 66.1%]; H = 1.13 [1.00; 1.72]

Test of heterogeneity:

Q d.f. p-value

6.39 5 0.2701

> meta1=metacont(n1,mean1,sd1,n2,mean2,sd2,data=dat,sm="MD",studlab=study)

> meta1=metacont(n1,mean1,sd1,n2,mean2,sd2,data=dat,sm="MD",byvar=subgroup,studlab=study,comb.random=TRUE,comb.fixed=FALSE,label.e="NIBS",label.c="Control")

>

> forest(meta1)

> metainf(meta1)

Influential analysis (random effects model)

MD 95%-CI p-value tau^2 tau I^2

Omitting Pavlova 2018a -1.1464 [-3.0263; 0.7336] 0.2320 0.0000 0.0028 25.6%

Omitting Pavlova 2018b -2.1225 [-4.6221; 0.3771] 0.0961 2.6220 1.6193 32.3%

Omitting Burkhardt 2023 -2.3315 [-4.8908; 0.2277] 0.0742 2.0675 1.4379 24.0%

Omitting Pavlova 2018a -0.8884 [-2.7739; 0.9970] 0.3557 0.0000 0.0000 0.0%

Omitting Pavlova 2018b -1.1549 [-3.0699; 0.7601] 0.2372 0.0000 0.0011 30.4%

Omitting Burkhardt 2023 -2.2711 [-5.0217; 0.4794] 0.1056 3.2268 1.7963 31.9%

Pooled estimate -1.4892 [-3.3830; 0.4047] 0.1233 0.3998 0.6323 21.8%

③ neurotransmitter

> dat=read.csv("D:/neurotransmitter.csv")

> dat

study subgroup n1 mean1 sd1

1 Zhang 2019 5-HT 50 98.59 24.66

2 Pu 2023 5-HT 50 111.09 27.64

3 Ma 2023 5-HT 100 98.58 25.52

4 Zhang 2019 DA 50 352.97 68.35

5 Ma 2023 DA 100 352.38 78.31

6 Zhang 2019 GABA 50 0.79 0.43

7 Ma 2023 GABA 100 0.80 0.43

n2 mean2 sd2

1 50 72.55 16.01

2 50 104.97 25.90

3 100 73.08 21.27

4 50 226.60 65.32

5 100 225.98 66.20

6 50 0.20 0.34

7 100 0.23 0.35

> meta1=metacont(n1,mean1,sd1,n2,mean2,sd2,data=dat,sm="SMD",studlab=study)

> meta1

Number of studies combined: k = 7

Number of observations: o = 1000

SMD

Common effect model 1.2911

Random effects model 1.2995

95%-CI

Common effect model [1.1529; 1.4293]

Random effects model [0.8985; 1.7004]

z p-value

Common effect model 18.31 < 0.0001

Random effects model 6.35 < 0.0001

Quantifying heterogeneity:

tau^2 = 0.2543 [0.0827; 1.4059]; tau = 0.5043 [0.2877; 1.1857]

I^2 = 86.7% [74.7%; 93.0%]; H = 2.74 [1.99; 3.77]

Test of heterogeneity:

Q d.f. p-value

45.04 6 < 0.0001

Details on meta-analytical method:

- Inverse variance method

- Restricted maximum-likelihood estimator for tau^2

- Q-Profile method for confidence interval of tau^2 and tau

- Hedges' g (bias corrected standardised mean difference; using exact formulae)

> meta1=metacont(n1,mean1,sd1,n2,mean2,sd2,data=dat,sm="SMD",studlab=study,comb.random=TRUE,comb.fixed=FALSE,label.e="NIBS",label.c="Control")

> meta1=metacont(n1,mean1,sd1,n2,mean2,sd2,data=dat,sm="SMD",byvar=subgroup,studlab=study,comb.random=TRUE,comb.fixed=FALSE,label.e="NIBS",label.c="Control")

> forest(meta1)

> metainf(meta1)

Influential analysis (random effects model)

SMD 95%-CI p-value tau^2 tau I^2

Omitting Zhang 2019 1.3093 [0.8366; 1.7820] < 0.0001 0.3118 0.5584 88.9%

Omitting Pu 2023 1.4655 [1.2253; 1.7058] < 0.0001 0.0533 0.2308 61.4%

Omitting Ma 2023 1.3385 [0.8679; 1.8091] < 0.0001 0.3044 0.5518 88.3%

Omitting Zhang 2019 1.2105 [0.7886; 1.6324] < 0.0001 0.2424 0.4924 87.1%

Omitting Ma 2023 1.2236 [0.7807; 1.6665] < 0.0001 0.2658 0.5156 86.2%

Omitting Zhang 2019 1.2664 [0.8008; 1.7320] < 0.0001 0.3019 0.5495 88.6%

Omitting Ma 2023 1.2741 [0.7998; 1.7485] < 0.0001 0.3104 0.5571 88.6%

Pooled estimate 1.2995 [0.8985; 1.7004] < 0.0001 0.2543 0.5043 86.7%

④ respond

dat=read.csv("D:/respond.csv")

dat

study subgroup event1 n1 event2 n2

1 Brunoni 2013 tDCS 19 30 10 30

2 Zhang 2020 tDCS 18 35 11 35

3 Pavlova 2018a tDCS 15 22 10 20

4 Pavlova 2018b tDCS 24 27 10 20

5 Burkhardt 2023 tDCS 25 77 32 73

6 Kumari 2023 tDCS 8 26 6 24

7 Huang 2012 rTMS 16 28 8 28

8 Ma 2023 rTMS 60 100 46 100

9 Ullrich 2012 rTMS 4 22 0 21

10 Rossini 2005 rTMS 25 50 22 49

11 Akpinar 2022 rTMS 13 20 4 18

12 Rumi 2005 rTMS 21 22 11 24

13 Wang 2017 rTMS 21 22 15 21

> level= 0.95

> meta1=metabin(event1,n1, event2, n2, studlab=study, data=dat, sm="OR",level= 0.95, leve1.comb=level)

> meta1

Number of studies combined: k = 13

Number of observations: o = 944

Number of events: e = 454

OR 95%-CI z p-value

Common effect model 1.9818 [1.5144; 2.5934] 4.98 < 0.0001

Random effects model 2.5258 [1.5300; 4.1696] 3.62 0.0003

Quantifying heterogeneity:

tau^2 = 0.4466 [0.0705; 2.1986]; tau = 0.6683 [0.2655; 1.4828]

I^2 = 59.7% [25.9%; 78.1%]; H = 1.58 [1.16; 2.14]

Test of heterogeneity:

Q d.f. p-value

29.79 12 0.0030

Details on meta-analytical method:

- Mantel-Haenszel method

- Restricted maximum-likelihood estimator for tau^2

- Q-Profile method for confidence interval of tau^2 and tau

- Continuity correction of 0.5 in studies with

zero cell frequencies

> meta1=metabin(event1,n1, event2, n2, studlab=study, data=dat, sm="OR", level= 0.95, leve1.comb=level,comb.fixed=FALSE, comb.random=TRUE,label.e="NIBS",label.c="Control")> meta1=metabin(event1,n1, event2, n2, data=dat,sm="OR",byvar=subgroup,studlab=study,comb.fixed=FALSE, comb.random=TRUE,label.e="NIBS",label.c="Control")> meta1=metabin(event1,n1, event2, n2, data=dat,sm="OR",byvar=subgroup,studlab=study,comb.fixed=FALSE,comb.random=TRUE,label.e="NIBS",label.c="Control")> forest(meta1)> metainf(meta1)

Influential analysis (random effects model)

OR 95%-CI

Omitting Brunoni 2013 2.4949 [1.4458; 4.3054]

Omitting Zhang 2020 2.6224 [1.4987; 4.5884]

Omitting Pavlova 2018a 2.6223 [1.5137; 4.5427]

Omitting Pavlova 2018b 2.2890 [1.3962; 3.7527]

Omitting Burkhardt 2023 2.7261 [1.8113; 4.1027]

Omitting Kumari 2023 2.7173 [1.5770; 4.6824]

Omitting Huang 2012 2.5079 [1.4532; 4.3282]

Omitting Ma 2023 2.7356 [1.5467; 4.8386]

Omitting Ullrich 2012 2.4337 [1.4707; 4.0274]

Omitting Rossini 2005 2.7922 [1.6163; 4.8235]

Omitting Akpinar 2022 2.3383 [1.4090; 3.8804]

Omitting Rumi 2005 2.2340 [1.3952; 3.5769]

Omitting Wang 2017 2.3977 [1.4473; 3.9723]

Pooled estimate 2.5258 [1.5300; 4.1696]

p-value tau^2 tau

Omitting Brunoni 2013 0.0010 0.5064 0.7116

Omitting Zhang 2020 0.0007 0.5435 0.7372

Omitting Pavlova 2018a 0.0006 0.5279 0.7266

Omitting Pavlova 2018b 0.0010 0.3819 0.6179

Omitting Burkhardt 2023 < 0.0001 0.1444 0.3800

Omitting Kumari 2023 0.0003 0.5115 0.7152

Omitting Huang 2012 0.0010 0.5094 0.7137

Omitting Ma 2023 0.0005 0.5532 0.7437

Omitting Ullrich 2012 0.0005 0.4363 0.6606

Omitting Rossini 2005 0.0002 0.4939 0.7028

Omitting Akpinar 2022 0.0010 0.4129 0.6426

Omitting Rumi 2005 0.0008 0.3371 0.5806

Omitting Wang 2017 0.0007 0.4291 0.6551

Pooled estimate 0.0003 0.4466 0.6683

I^2

Omitting Brunoni 2013 61.3%

Omitting Zhang 2020 62.8%

Omitting Pavlova 2018a 63.0%

Omitting Pavlova 2018b 57.7%

Omitting Burkhardt 2023 33.8%

Omitting Kumari 2023 62.7%

Omitting Huang 2012 61.6%

Omitting Ma 2023 63.0%

Omitting Ullrich 2012 61.4%

Omitting Rossini 2005 61.4%

Omitting Akpinar 2022 59.0%

Omitting Rumi 2005 54.5%

Omitting Wang 2017 60.7%

Pooled estimate 59.7%

Details on meta-analytical method:

- Mantel-Haenszel method

- Restricted maximum-likelihood estimator for tau^2

> meta1=metabin(event1,n1, event2, n2, studlab=study, data=dat, sm="OR", level= 0.95, leve1.comb=level,comb.fixed=FALSE, comb.random=TRUE,label.e="NIBS",label.c="Control")

metabias(meta1)

Linear regression test of funnel plot asymmetry

Test result: t = 3.34, df = 11, p-value = 0.0066

Sample estimates:

bias se.bias intercept se.intercept

3.2946 0.9867 -0.8302 0.4838

Details:

- multiplicative residual heterogeneity variance (tau^2 = 1.4294)

- predictor: standard error of score

- weight: inverse variance of score

- reference: Harbord et al. (2006), Stat Med

summary(mytrimfill)

OR 95%-CI %W(random)

Brunoni 2013 3.4545 [1.1946; 9.9900] 6.8

Zhang 2020 2.3102 [0.8723; 6.1185] 7.1

Pavlova 2018a 2.1429 [0.6113; 7.5112] 6.2

Pavlova 2018b 8.0000 [1.8098; 35.3627] 5.6

Burkhardt 2023 0.6160 [0.3170; 1.1969] 7.9

Kumari 2023 1.3333 [0.3843; 4.6254] 6.3

Huang 2012 3.3333 [1.0983; 10.1162] 6.7

Ma 2023 1.7609 [1.0048; 3.0858] 8.2

Ullrich 2012 10.4595 [0.5275; 207.3956] 2.6

Rossini 2005 1.2273 [0.5570; 2.7043] 7.6

Akpinar 2022 6.5000 [1.5371; 27.4863] 5.7

Rumi 2005 24.8182 [2.8598; 215.3766] 3.9

Wang 2017 8.4000 [0.9139; 77.2080] 3.8

Filled: Akpinar 2022 0.3566 [0.0843; 1.5079] 5.7

Filled: Pavlova 2018b 0.2897 [0.0655; 1.2807] 5.6

Filled: Wang 2017 0.2759 [0.0300; 2.5363] 3.8

Filled: Ullrich 2012 0.2216 [0.0112; 4.3941] 2.6

Filled: Rumi 2005 0.0934 [0.0108; 0.8105] 3.9

Number of studies combined: k = 18 (with 5 added studies)

Number of observations: o = 1161

Number of events: e = 577

OR 95%-CI z p-value

Random effects model 1.6398 [0.9222; 2.9160] 1.68 0.0922

Quantifying heterogeneity:

tau^2 = 0.9738 [0.3872; 4.2678]; tau = 0.9868 [0.6222; 2.0659]

I^2 = 66.6% [45.2%; 79.6%]; H = 1.73 [1.35; 2.21]

Test of heterogeneity:

Q d.f. p-value

50.84 17 < 0.0001

OR 95%-CI %W(common) %W(random)

Brunoni 2013 3.4545 [1.1946; 9.9900] 4.8 8.8

Zhang 2020 2.3102 [0.8723; 6.1185] 7.0 9.4

Pavlova 2018a 2.1429 [0.6113; 7.5112] 4.4 7.6

Pavlova 2018b 8.0000 [1.8098; 35.3627] 1.7 6.4

Burkhardt 2023 0.6160 [0.3170; 1.1969] 29.1 11.6

Kumari 2023 1.3333 [0.3843; 4.6254] 5.7 7.7

Huang 2012 3.3333 [1.0983; 10.1162] 4.5 8.5

Ma 2023 1.7609 [1.0048; 3.0858] 24.2 12.4

Ullrich 2012 10.4595 [0.5275; 207.3956] 0.5 2.4

Rossini 2005 1.2273 [0.5570; 2.7043] 14.6 10.7

Akpinar 2022 6.5000 [1.5371; 27.4863] 1.9 6.6

Rumi 2005 24.8182 [2.8598; 215.3766] 0.6 3.9

Wang 2017 8.4000 [0.9139; 77.2080] 0.9 3.8

Number of studies combined: k = 13

Number of observations: o = 944

Number of events: e = 454

OR 95%-CI z p-value

Common effect model 1.9818 [1.5144; 2.5934] 4.98 < 0.0001

Random effects model 2.5258 [1.5300; 4.1696] 3.62 0.0003

Quantifying heterogeneity:

tau^2 = 0.4466 [0.0705; 2.1986]; tau = 0.6683 [0.2655; 1.4828]

I^2 = 59.7% [25.9%; 78.1%]; H = 1.58 [1.16; 2.14]

Test of heterogeneity:

Q d.f. p-value

29.79 12 0.0030

⑤ remit

> dat=read.csv("D:/remit.csv")> dat study subgroup event1 n1

1 Brunoni 2013 tDCS 14 30

2 Zhang 2020 tDCS 6 35

3 Pavlova 2018a tDCS 6 22

4 Pavlova 2018b tDCS 19 27

5 Burkhardt 2023 tDCS 24 77

6 Kumari 2023 tDCS 3 26

7 Huang 2012 rTMS 11 28

8 Ullrich 2012 rTMS 0 22

9 Rossini 2005 rTMS 18 50

10 Rumi 2005 rTMS 12 22

11 Wang 2017 rTMS 15 22

event2 n2

1 9 30

2 3 35

3 7 20

4 7 20

5 28 73

6 1 24

7 7 28

8 0 21

9 5 49

10 3 24

11 8 21

> level= 0.95> meta1=metabin(event1,n1, event2, n2, studlab=study, data=dat, sm="OR",level= 0.95, leve1.comb=level)> meta1Number of studies combined: k = 10

Number of observations: o = 706

Number of events: e = 206

OR

Common effect model 1.9309

Random effects model 2.2644

95%-CI z

Common effect model [1.3676; 2.7263] 3.74

Random effects model [1.3043; 3.9313] 2.90

p-value

Common effect model 0.0002

Random effects model 0.0037

Quantifying heterogeneity:

tau^2 = 0.3879 [0.0084; 1.7345]; tau = 0.6228 [0.0919; 1.3170]

I^2 = 54.5% [7.2%; 77.7%]; H = 1.48 [1.04; 2.12]

Test of heterogeneity:

Q d.f. p-value

19.78 9 0.0193

Details on meta-analytical method:

- Mantel-Haenszel method

- Restricted maximum-likelihood estimator for tau^2

- Q-Profile method for confidence interval of tau^2 and tau> meta1=metabin(event1,n1, event2, n2, studlab=study, data=dat, sm="OR", level= 0.95, leve1.comb=level,comb.fixed=TRUE, comb.random=FALSE,label.e="NIBS",label.c="Control")> meta1=metabin(event1,n1, event2, n2, data=dat,sm="OR",byvar=subgroup,studlab=study, comb.fixed=TRUE, comb.random=FALSE,label.e="NIBS",label.c="Control")> meta1=metabin(event1,n1, event2, n2, data=dat,sm="OR",byvar=subgroup,studlab=study, comb.fixed=TRUE, comb.random=FALSE,label.e="NIBS",label.c="Control")> forest(meta1)> metainf(meta1)Influential analysis (common effect model)

OR

Omitting Brunoni 2013 1.9184

Omitting Zhang 2020 1.9156

Omitting Pavlova 2018a 2.0886

Omitting Pavlova 2018b 1.7988

Omitting Burkhardt 2023 2.8025

Omitting Kumari 2023 1.9096

Omitting Huang 2012 1.9299

Omitting Ullrich 2012 1.9309

Omitting Rossini 2005 1.7085

Omitting Rumi 2005 1.7467

Omitting Wang 2017 1.8401

Pooled estimate 1.9309

95%-CI

Omitting Brunoni 2013 [1.3320; 2.7628]

Omitting Zhang 2020 [1.3433; 2.7315]

Omitting Pavlova 2018a [1.4576; 2.9928]

Omitting Pavlova 2018b [1.2547; 2.5787]

Omitting Burkhardt 2023 [1.8488; 4.2483]

Omitting Kumari 2023 [1.3470; 2.7072]

Omitting Huang 2012 [1.3440; 2.7712]

Omitting Ullrich 2012 [1.3676; 2.7263]

Omitting Rossini 2005 [1.1833; 2.4667]

Omitting Rumi 2005 [1.2208; 2.4991]

Omitting Wang 2017 [1.2850; 2.6352]

Pooled estimate [1.3676; 2.7263]

p-value tau^2

Omitting Brunoni 2013 0.0005 0.4746

Omitting Zhang 2020 0.0003 0.4512

Omitting Pavlova 2018a < 0.0001 0.3622

Omitting Pavlova 2018b 0.0014 0.4013

Omitting Burkhardt 2023 < 0.0001 0.0587

Omitting Kumari 2023 0.0003 0.4171

Omitting Huang 2012 0.0004 0.4690

Omitting Ullrich 2012 0.0002 0.3879

Omitting Rossini 2005 0.0043 0.3659

Omitting Rumi 2005 0.0023 0.3151

Omitting Wang 2017 0.0009 0.4343

Pooled estimate 0.0002 0.3879

tau I^2

Omitting Brunoni 2013 0.6889 59.5%

Omitting Zhang 2020 0.6717 59.5%

Omitting Pavlova 2018a 0.6018 53.9%

Omitting Pavlova 2018b 0.6335 55.1%

Omitting Burkhardt 2023 0.2423 10.8%

Omitting Kumari 2023 0.6458 59.2%

Omitting Huang 2012 0.6848 59.5%

Omitting Ullrich 2012 0.6228 54.5%

Omitting Rossini 2005 0.6049 51.3%

Omitting Rumi 2005 0.5613 48.7%

Omitting Wang 2017 0.6590 57.4%

Pooled estimate 0.6228 54.5%

Details on meta-analytical method:

- Mantel-Haenszel method

- Restricted maximum-likelihood estimator for tau^2

> forest(metainf(meta1))> meta1=metabin(event1,n1, event2, n2, studlab=study, data=dat, sm="OR", level= 0.95, leve1.comb=level,comb.fixed=FALSE, comb.random=TRUE,label.e="NIBS",label.c="Control")

> metabias(meta1)

Linear regression test of funnel plot asymmetry

Sample estimates:Test result: t = 1.54, df = 8, p-value = 0.1619

bias se.bias intercept se.intercept

2.5048 1.6256 -0.6649 0.8928

Details:

- multiplicative residual heterogeneity variance (tau^2 = 1.8676)

- predictor: standard error of score

- weight: inverse variance of score

- reference: Harbord et al. (2006), Stat Med

⑥ droup out rate

> dat=read.csv("D:/dropout rate.csv")

> dat

study subgroup event1 n1 event2

1 Herwig 2007 rTMS 10 62 12

2 Wang 2017 rTMS 3 25 2

3 Bretlau 2008 rTMS 1 24 3

4 Huang 2012 rTMS 2 30 2

5 Pu 2023 rTMS 8 50 10

6 Rossini 2005 rTMS 1 50 2

7 Brunoni 2013 tDCS 12 30 9

8 Kumari 2023 tDCS 4 26 2

9 Pavlova 2018a tDCS 3 22 0

10 Pavlova 2018b tDCS 2 27 0

11 Burkhardt 2023 tDCS 4 77 9

n2

1 65

2 23

3 25

4 30

5 50

6 49

7 30

8 24

9 20

10 20

11 73

> library(meta)

> level= 0.95

> meta1=metabin(event1,n1, event2, n2, studlab=study, data=dat, sm="OR",level= 0.95, leve1.comb=level)

> meta1

Number of studies combined: k = 11

Number of observations: o = 832

Number of events: e = 101

OR 95%-CI

Common effect model 0.9579 [0.6267; 1.4642]

Random effects model 0.9338 [0.5989; 1.4561]

z p-value

Common effect model -0.20 0.8426

Random effects model -0.30 0.7626

Quantifying heterogeneity:

tau^2 = 0 [0.0000; 1.3037]; tau = 0 [0.0000; 1.1418]

I^2 = 0.0% [0.0%; 60.2%]; H = 1.00 [1.00; 1.59]

Test of heterogeneity:

Q d.f. p-value

7.66 10 0.6623

Details on meta-analytical method:

- Mantel-Haenszel method

- Restricted maximum-likelihood estimator for tau^2

- Q-Profile method for confidence interval of tau^2 and tau

- Continuity correction of 0.5 in studies with

zero cell frequencies

> meta1=metabin(event1,n1, event2, n2, studlab=study, data=dat, sm="OR", level= 0.95, leve1.comb=level,comb.fixed=TRUE, comb.random=FALSE,label.e="NIBS",label.c="Control")

> meta1=metabin(event1,n1, event2, n2, data=dat,sm="OR",byvar=subgroup,studlab=study, comb.fixed=TRUE, comb.random=FALSE,label.e="NIBS",label.c="Control")

> meta1=metabin(event1,n1, event2, n2, data=dat,sm="OR",byvar=subgroup,studlab=study, comb.fixed=TRUE, comb.random=FALSE,label.e="NIBS",label.c="Control")

> forest(meta1)

> metainf(meta1)

Influential analysis (common effect model)

OR

Omitting Herwig 2007 0.9895

Omitting Wang 2017 0.9371

Omitting Bretlau 2008 1.0021

Omitting Huang 2012 0.9561

Omitting Pu 2023 1.0047

Omitting Rossini 2005 0.9807

Omitting Brunoni 2013 0.8735

Omitting Kumari 2023 0.9141

Omitting Pavlova 2018a 0.8922

Omitting Pavlova 2018b 0.9210

Omitting Burkhardt 2023 1.1008

Pooled estimate 0.9579

95%-CI

Omitting Herwig 2007 [0.6134; 1.5962]

Omitting Wang 2017 [0.6060; 1.4492]

Omitting Bretlau 2008 [0.6494; 1.5462]

Omitting Huang 2012 [0.6195; 1.4755]

Omitting Pu 2023 [0.6301; 1.6021]

Omitting Rossini 2005 [0.6369; 1.5102]

Omitting Brunoni 2013 [0.5492; 1.3892]

Omitting Kumari 2023 [0.5897; 1.4170]

Omitting Pavlova 2018a [0.5779; 1.3775]

Omitting Pavlova 2018b [0.5984; 1.4175]

Omitting Burkhardt 2023 [0.6959; 1.7413]

Pooled estimate [0.6267; 1.4642]

p-value tau^2

Omitting Herwig 2007 0.9656 0.0000

Omitting Wang 2017 0.7704 0.0000

Omitting Bretlau 2008 0.9926 0.0000

Omitting Huang 2012 0.8391 0.0000

Omitting Pu 2023 0.9843 0.0000

Omitting Rossini 2005 0.9294 0.0000

Omitting Brunoni 2013 0.5677 0.0000

Omitting Kumari 2023 0.6880 0.0000

Omitting Pavlova 2018a 0.6068 0.0000

Omitting Pavlova 2018b 0.7082 0.0000

Omitting Burkhardt 2023 0.6815 0.0000

Pooled estimate 0.8426 0.0000

tau I^2

Omitting Herwig 2007 0.0000 0.0%

Omitting Wang 2017 0.0000 0.0%

Omitting Bretlau 2008 0.0000 0.0%

Omitting Huang 2012 0.0000 0.0%

Omitting Pu 2023 0.0000 0.0%

Omitting Rossini 2005 0.0000 0.0%

Omitting Brunoni 2013 0.0000 0.0%

Omitting Kumari 2023 0.0000 0.0%

Omitting Pavlova 2018a 0.0000 0.0%

Omitting Pavlova 2018b 0.0000 0.0%

Omitting Burkhardt 2023 0.0000 0.0%

Pooled estimate 0.0000 0.0%

metabias(meta1)

Linear regression test of funnel plot asymmetry

Test result: t = 1.24, df = 9, p-value = 0.2449

Sample estimates:

bias se.bias intercept se.intercept

1.0177 0.8181 -0.7269 0.5912

⑦ follow-up

> dat=read.csv("D:/follow up.csv")

> dat

study subgroup n1 mean1 sd1 n2 mean2 sd2

1 Bretlau2008 2 weeks 22 -10.80 4.52 23 -8.40 4.46

2 Huang 2012 2 weeks 28 -10.58 1.97 28 -7.57 2.35

3 Kumari 2023 2 weeks 26 -7.50 3.39 24 -7.21 3.44

4 Wang 2017 3 weeks 22 -36.18 8.73 21 -36.67 8.05

5 Rossini 2005 3 weeks 50 -19.10 1.12 49 -16.20 1.14

6 Burkhardt 2023 3month 77 -13.68 5.52 73 -13.31 4.44

7 Burkhardt 2023 6month 77 -11.14 5.40 73 -12.71 4.42

> meta1=metacont(n1,mean1,sd1,n2,mean2,sd2,data=dat,sm="SMD",studlab=study)

> meta1

Number of studies combined: k = 7

Number of observations: o = 593

SMD

Common effect model -0.3509

Random effects model -0.5944

95%-CI z

Common effect model [-0.5207; -0.1810] -4.05

Random effects model [-1.3474; 0.1587] -1.55

p-value

Common effect model < 0.0001

Random effects model 0.1219

Quantifying heterogeneity:

tau^2 = 0.9651 [0.3611; 4.9423]; tau = 0.9824 [0.6009; 2.2231]

I^2 = 93.9% [89.9%; 96.4%]; H = 4.06 [3.14; 5.24]

Test of heterogeneity:

Q d.f. p-value

98.69 6 < 0.0001

Details on meta-analytical method:

- Inverse variance method

- Restricted maximum-likelihood estimator for tau^2

- Q-Profile method for confidence interval of tau^2 and tau

- Hedges' g (bias corrected standardised mean difference; using exact formulae)

> meta1=metacont(n1,mean1,sd1,n2,mean2,sd2,data=dat,sm="SMD",studlab=study,comb.random=TRUE,comb.fixed=FALSE,label.e="NIBS",label.c="Control")

> meta1=metacont(n1,mean1,sd1,n2,mean2,sd2,data=dat,sm="SMD",byvar=subgroup,studlab=study,comb.random=TRUE,comb.fixed=FALSE,label.e="NIBS",label.c="Control")

> forest(meta1)

> metainf(meta1)

Influential analysis (random effects model)

SMD 95%-CI p-value tau^2 tau I^2

Omitting Bretlau2008 -0.6074 [-1.4954; 0.2805] 0.1800 1.1670 1.0803 94.9%

Omitting Huang 2012 -0.4688 [-1.3066; 0.3691] 0.2728 1.0316 1.0157 94.2%

Omitting Kumari 2023 -0.6799 [-1.5485; 0.1888] 0.1250 1.1121 1.0546 94.9%

Omitting Wang 2017 -0.7013 [-1.5557; 0.1530] 0.1076 1.0758 1.0372 94.8%

Omitting Rossini 2005 -0.2535 [-0.7221; 0.2151] 0.2890 0.2775 0.5268 81.2%

Omitting Burkhardt 2023 -0.6863 [-1.5582; 0.1856] 0.1229 1.1118 1.0544 94.7%

Omitting Burkhardt 2023 -0.7535 [-1.5719; 0.0650] 0.0712 0.9707 0.9852 93.4%

Pooled estimate -0.5944 [-1.3474; 0.1587] 0.1219 0.9651 0.9824 93.9%

Meta-regression

regression data remit

data<-read_xlsx("D:\\regression data depression.xlsx")

> colnames(data)

[1] "study" "Class of antidepressant" "Sample size" "Age"

[5] "Female rate" "Baseline score" "Type of NIBS" "Severity of depression"

[9] "Country" "Total Session" "Publication Year" "n1"

[13] "mean1" "sd1" "n2" "mean2"

[17] "sd2"

> table(data$`Type of NIBS`)

rTMS tDCS

11 8

> table(data$`Total Session`)

10 15 20 24 30 40 48

8 3 4 1 1 1 1

> table(data$`Severity of depression`)

Major depression Major depression Mild to moderate depressive

2 12 4

Moderate to severe depression

1

> table(data$`Baseline score`)

16.9 17.1 19.39 20.35 20.9 21.7 22.13 22.18 22.65 23.45 23.72 25 25.1 27 27.43 29.32 30.34 30.6

1 1 1 1 1 1 1 1 1 1 1 1 1 1 1 1 1 1

43.16

1

> table(data$`Class of antidepressant`)

New type of antidepressant SNRIs SNRIs or NaSSAs SSRI

1 4 1 2

SSRIs SSRIs or SNRIs TCAs

8 2 1

> table(data$`Sample size`)

24 30 37 38 42 43 45 46 47 50 56 70 77 99 100 127 200

1 1 1 1 1 2 1 1 1 1 1 1 1 1 2 1 1

> table(data$`Age`)

29.42 30.75 32.06 32.64 34.06 37.6 38.31 39.09 40.1 41 43.67 44.21 44.5 44.7 47.7 49.5 55.5 61.8

1 1 1 1 1 1 1 1 1 1 1 1 1 1 1 1 2 1

> table(data$`Female rate`)

0.4 0.46 0.5 0.57 0.59 0.6 0.62 0.68 0.7 0.75 0.78 0.8 0.84 0.85

1 2 1 1 3 1 1 3 1 1 1 1 1 1

> data$`Publication Year`<-as.character(data$`Type of NIBS`)

> data$`Publication Year`<-as.character(data$`Total Session`)

> data$`Publication Year`<-as.character(data$`Severity of depression`)

> data$`Publication Year`<-as.character(data$`Baseline score`)

> data$`Publication Year`<-as.character(data$`Class of antidepressant`)

> data$`Publication Year`<-as.character(data$`Sample size`)

> data$`Publication Year`<-as.character(data$`Age`)

> data$`Publication Year`<-as.character(data$`Female rate`)

> library(meta)

> m1 = metacont(n1,mean1,sd1,n2,mean2,sd2,sm='SMD',

+ studlab = study,data,common=FALSE)

> metareg(m1,~`Type of NIBS`)

Mixed-Effects Model (k = 19; tau^2 estimator: REML)

tau^2 (estimated amount of residual heterogeneity): 1.2551 (SE = 0.4625)

tau (square root of estimated tau^2 value): 1.1203

I^2 (residual heterogeneity / unaccounted variability): 94.76%

H^2 (unaccounted variability / sampling variability): 19.07

R^2 (amount of heterogeneity accounted for): 5.62%

Test for Residual Heterogeneity:

QE(df = 17) = 191.1749, p-val < .0001

Test of Moderators (coefficient 2):

QM(df = 1) = 2.1548, p-val = 0.1421

Model Results:

estimate se zval pval ci.lb ci.ub

intrcpt -1.3461 0.3505 -3.8406 0.0001 -2.0331 -0.6592 ***

`Type of NIBS`tDCS 0.7921 0.5396 1.4679 0.1421 -0.2655 1.8498

---

Signif. codes: 0 ‘***’ 0.001 ‘**’ 0.01 ‘*’ 0.05 ‘.’ 0.1 ‘ ’ 1

> metareg(m1,~`Total Session`)

Mixed-Effects Model (k = 19; tau^2 estimator: REML)

tau^2 (estimated amount of residual heterogeneity): 1.4264 (SE = 0.5219)

tau (square root of estimated tau^2 value): 1.1943

I^2 (residual heterogeneity / unaccounted variability): 95.41%

H^2 (unaccounted variability / sampling variability): 21.77

R^2 (amount of heterogeneity accounted for): 0.00%

Test for Residual Heterogeneity:

QE(df = 17) = 210.3307, p-val < .0001

Test of Moderators (coefficient 2):

QM(df = 1) = 0.0079, p-val = 0.9291

Model Results:

estimate se zval pval ci.lb ci.ub

intrcpt -1.0590 0.5646 -1.8756 0.0607 -2.1657 0.0476 .

`Total Session` 0.0024 0.0266 0.0890 0.9291 -0.0497 0.0544

---

Signif. codes: 0 ‘***’ 0.001 ‘**’ 0.01 ‘*’ 0.05 ‘.’ 0.1 ‘ ’ 1

> metareg(m1,~`Severity of depression`)

Mixed-Effects Model (k = 19; tau^2 estimator: REML)

tau^2 (estimated amount of residual heterogeneity): 1.5371 (SE = 0.5974)

tau (square root of estimated tau^2 value): 1.2398

I^2 (residual heterogeneity / unaccounted variability): 95.46%

H^2 (unaccounted variability / sampling variability): 22.00

R^2 (amount of heterogeneity accounted for): 0.00%

Test for Residual Heterogeneity:

QE(df = 15) = 195.8741, p-val < .0001

Test of Moderators (coefficients 2:4):

QM(df = 3) = 1.1140, p-val = 0.7737

Model Results:

estimate se zval pval ci.lb ci.ub

intrcpt -0.6049 0.9036 -0.6694 0.5033 -2.3759 1.1662

`Severity of depression`Major depression -0.6364 0.9765 -0.6517 0.5146 -2.5503 1.2775

`Severity of depression`Mild to moderate depressive -0.1558 1.1042 -0.1411 0.8878 -2.3200 2.0084

`Severity of depression`Moderate to severe depression 0.3556 1.5445 0.2302 0.8179 -2.6715 3.3827

---

Signif. codes: 0 ‘***’ 0.001 ‘**’ 0.01 ‘*’ 0.05 ‘.’ 0.1 ‘ ’ 1

> metareg(m1,~`Baseline score`)

Mixed-Effects Model (k = 19; tau^2 estimator: REML)

tau^2 (estimated amount of residual heterogeneity): 1.3889 (SE = 0.5083)

tau (square root of estimated tau^2 value): 1.1785

I^2 (residual heterogeneity / unaccounted variability): 95.38%

H^2 (unaccounted variability / sampling variability): 21.63

R^2 (amount of heterogeneity accounted for): 0.00%

Test for Residual Heterogeneity:

QE(df = 17) = 204.8757, p-val < .0001

Test of Moderators (coefficient 2):

QM(df = 1) = 0.4616, p-val = 0.4969

Model Results:

estimate se zval pval ci.lb ci.ub

intrcpt -0.2140 1.2113 -0.1767 0.8597 -2.5882 2.1601

`Baseline score` -0.0325 0.0479 -0.6794 0.4969 -0.1264 0.0613

---

Signif. codes: 0 ‘***’ 0.001 ‘**’ 0.01 ‘*’ 0.05 ‘.’ 0.1 ‘ ’ 1

> metareg(m1,~`Class of antidepressant`)

Mixed-Effects Model (k = 19; tau^2 estimator: REML)

tau^2 (estimated amount of residual heterogeneity): 0.9924 (SE = 0.4416)

tau (square root of estimated tau^2 value): 0.9962

I^2 (residual heterogeneity / unaccounted variability): 92.80%

H^2 (unaccounted variability / sampling variability): 13.89

R^2 (amount of heterogeneity accounted for): 25.37%

Test for Residual Heterogeneity:

QE(df = 12) = 125.2821, p-val < .0001

Test of Moderators (coefficients 2:7):

QM(df = 6) = 12.4037, p-val = 0.0535

Model Results:

estimate se zval pval ci.lb ci.ub

intrcpt -0.5536 1.0214 -0.5420 0.5878 -2.5554 1.4483

`Class of antidepressant`SNRIs -0.3541 1.1439 -0.3095 0.7569 -2.5961 1.8880

`Class of antidepressant`SNRIs or NaSSAs 0.3043 1.4379 0.2117 0.8324 -2.5138 3.1225

`Class of antidepressant`SSRI 0.1084 1.2507 0.0867 0.9309 -2.3429 2.5597

`Class of antidepressant`SSRIs -0.2365 1.0861 -0.2177 0.8277 -2.3652 1.8923

`Class of antidepressant`SSRIs or SNRIs -1.2983 1.2661 -1.0254 0.3052 -3.7797 1.1832

`Class of antidepressant`TCAs -3.9758 1.5380 -2.5850 0.0097 -6.9903 -0.9613 **

---

Signif. codes: 0 ‘***’ 0.001 ‘**’ 0.01 ‘*’ 0.05 ‘.’ 0.1 ‘ ’ 1

> metareg(m1,~`Sample size`)

Mixed-Effects Model (k = 19; tau^2 estimator: REML)

tau^2 (estimated amount of residual heterogeneity): 1.3845 (SE = 0.5078)

tau (square root of estimated tau^2 value): 1.1766

I^2 (residual heterogeneity / unaccounted variability): 95.08%

H^2 (unaccounted variability / sampling variability): 20.34

R^2 (amount of heterogeneity accounted for): 0.00%

Test for Residual Heterogeneity:

QE(df = 17) = 196.6211, p-val < .0001

Test of Moderators (coefficient 2):

QM(df = 1) = 0.4261, p-val = 0.5139

Model Results:

estimate se zval pval ci.lb ci.ub

intrcpt -0.7221 0.5280 -1.3678 0.1714 -1.7569 0.3126

`Sample size` -0.0043 0.0066 -0.6528 0.5139 -0.0172 0.0086

---

Signif. codes: 0 ‘***’ 0.001 ‘**’ 0.01 ‘*’ 0.05 ‘.’ 0.1 ‘ ’ 1

> metareg(m1,~`Age`)

Mixed-Effects Model (k = 19; tau^2 estimator: REML)

tau^2 (estimated amount of residual heterogeneity): 1.4247 (SE = 0.5206)

tau (square root of estimated tau^2 value): 1.1936

I^2 (residual heterogeneity / unaccounted variability): 95.42%

H^2 (unaccounted variability / sampling variability): 21.81

R^2 (amount of heterogeneity accounted for): 0.00%

Test for Residual Heterogeneity:

QE(df = 17) = 208.1860, p-val < .0001

Test of Moderators (coefficient 2):

QM(df = 1) = 0.0010, p-val = 0.9747

Model Results:

estimate se zval pval ci.lb ci.ub

intrcpt -1.0590 1.4008 -0.7560 0.4496 -3.8045 1.6864

Age 0.0010 0.0326 0.0317 0.9747 -0.0628 0.0648

---

Signif. codes: 0 ‘***’ 0.001 ‘**’ 0.01 ‘*’ 0.05 ‘.’ 0.1 ‘ ’ 1

> metareg(m1,~`Female rate`)

Mixed-Effects Model (k = 19; tau^2 estimator: REML)

tau^2 (estimated amount of residual heterogeneity): 1.0705 (SE = 0.3986)

tau (square root of estimated tau^2 value): 1.0346

I^2 (residual heterogeneity / unaccounted variability): 94.10%

H^2 (unaccounted variability / sampling variability): 16.95

R^2 (amount of heterogeneity accounted for): 19.50%

Test for Residual Heterogeneity:

QE(df = 17) = 190.4554, p-val < .0001

Test of Moderators (coefficient 2):

QM(df = 1) = 5.2149, p-val = 0.0224

Model Results:

estimate se zval pval ci.lb ci.ub

intrcpt 1.8516 1.2760 1.4510 0.1468 -0.6494 4.3525

`Female rate` -4.5028 1.9718 -2.2836 0.0224 -8.3675 -0.6382 *

---

Signif. codes: 0 ‘***’ 0.001 ‘**’ 0.01 ‘*’ 0.05 ‘.’ 0.1 ‘ ’ 1

> data<-read_xlsx("D:\\regression data remit .xlsx")

> colnames(data)

[1] "study" "Country" "Baseline score"

[4] "Publication Year" "Class of antidepressant" "Type of NIBS"

[7] "Severity of depression" "Total Session" "Sample size"

[10] "Age" "Female rate" "event1"

[13] "n1" "event2" "n2"

> table(data$`Baseline score`)

16.9 17.1 19.39 20.9 21.7 22.65 25.1 29.32 30.34 30.75 43.16

1 1 1 1 1 1 1 1 1 1 1

> table(data$`Class of antidepressant`)

SNRIs SSRIs SSRIs or SNRIs TCAs

1 8 1 1

> table(data$`Type of NIBS`)

rTMS tDCS

5 6

> table(data$`Severity of depression`)

Major depression Major depression Mild to moderate depressive

1 8 2

> table(data$`Total Session`)

10 12 15 20 24 48

4 1 1 3 1 1

> table(data$`Sample size`)

42 43 46 47 50 56 60 70 99 150

1 2 1 1 1 1 1 1 1 1

> table(data$`Age`)

29.42 30.75 32.06 37.6 38.31 39.09 40.1 41 43.67 47.7 55.5

1 1 1 1 1 1 1 1 1 1 1

> table(data$`Female rate`)

0.4 0.46 0.5 0.59 0.63 0.68 0.7 0.78 0.8 0.85

1 1 1 1 1 2 1 1 1 1

> m1 = metabin( event1,n1,event2,n2,sm='OR',

+ studlab = study,data,common=FALSE)

> metareg(m1,~`Baseline score`)

Mixed-Effects Model (k = 10; tau^2 estimator: REML)

tau^2 (estimated amount of residual heterogeneity): 0.3661 (SE = 0.3741)

tau (square root of estimated tau^2 value): 0.6051

I^2 (residual heterogeneity / unaccounted variability): 50.63%

H^2 (unaccounted variability / sampling variability): 2.03

R^2 (amount of heterogeneity accounted for): 5.62%

Test for Residual Heterogeneity:

QE(df = 8) = 16.6577, p-val = 0.0339

Test of Moderators (coefficient 2):

QM(df = 1) = 1.1163, p-val = 0.2907

Model Results:

estimate se zval pval ci.lb ci.ub

intrcpt -0.1612 0.9638 -0.1672 0.8672 -2.0503 1.7279

`Baseline score` 0.0390 0.0369 1.0566 0.2907 -0.0334 0.1114

---

Signif. codes: 0 ‘***’ 0.001 ‘**’ 0.01 ‘*’ 0.05 ‘.’ 0.1 ‘ ’ 1

Warning message:

1 study with NAs omitted from model fitting.

> metareg(m1,~`Class of antidepressant`)

Mixed-Effects Model (k = 10; tau^2 estimator: REML)

tau^2 (estimated amount of residual heterogeneity): 0.2481 (SE = 0.3280)

tau (square root of estimated tau^2 value): 0.4981

I^2 (residual heterogeneity / unaccounted variability): 41.29%

H^2 (unaccounted variability / sampling variability): 1.70

R^2 (amount of heterogeneity accounted for): 36.05%

Test for Residual Heterogeneity:

QE(df = 7) = 11.4994, p-val = 0.1183

Test of Moderators (coefficients 2:3):

QM(df = 2) = 4.1294, p-val = 0.1269

Model Results:

estimate se zval pval ci.lb

intrcpt 2.1282 0.9013 2.3613 0.0182 0.3617

`Class of antidepressant`SSRIs -1.5822 0.9443 -1.6755 0.0938 -3.4329

`Class of antidepressant`SSRIs or SNRIs -0.5288 1.1705 -0.4518 0.6514 -2.8229

ci.ub

intrcpt 3.8948 *

`Class of antidepressant`SSRIs 0.2686 .

`Class of antidepressant`SSRIs or SNRIs 1.7652

---

Signif. codes: 0 ‘***’ 0.001 ‘**’ 0.01 ‘*’ 0.05 ‘.’ 0.1 ‘ ’ 1

Warning messages:

1: 1 study with NAs omitted from model fitting.

2: Redundant predictors dropped from the model.

> metareg(m1,~`Type of NIBS`)

Mixed-Effects Model (k = 10; tau^2 estimator: REML)

tau^2 (estimated amount of residual heterogeneity): 0.2073 (SE = 0.2908)

tau (square root of estimated tau^2 value): 0.4553

I^2 (residual heterogeneity / unaccounted variability): 35.89%

H^2 (unaccounted variability / sampling variability): 1.56

R^2 (amount of heterogeneity accounted for): 46.56%

Test for Residual Heterogeneity:

QE(df = 8) = 11.8319, p-val = 0.1589

Test of Moderators (coefficient 2):

QM(df = 1) = 3.7229, p-val = 0.0537

Model Results:

estimate se zval pval ci.lb ci.ub

intrcpt 1.3607 0.3871 3.5151 0.0004 0.6020 2.1194 ***

`Type of NIBS`tDCS -0.9615 0.4983 -1.9295 0.0537 -1.9382 0.0152 .

---

Signif. codes: 0 ‘***’ 0.001 ‘**’ 0.01 ‘*’ 0.05 ‘.’ 0.1 ‘ ’ 1

Warning message:

1 study with NAs omitted from model fitting.

> metareg(m1,~`Severity of depression`)

Mixed-Effects Model (k = 10; tau^2 estimator: REML)

tau^2 (estimated amount of residual heterogeneity): 0.5319 (SE = 0.4961)

tau (square root of estimated tau^2 value): 0.7293

I^2 (residual heterogeneity / unaccounted variability): 60.01%

H^2 (unaccounted variability / sampling variability): 2.50

R^2 (amount of heterogeneity accounted for): 0.00%

Test for Residual Heterogeneity:

QE(df = 7) = 18.7836, p-val = 0.0089

Test of Moderators (coefficients 2:3):

QM(df = 2) = 0.3110, p-val = 0.8560

Model Results:

estimate se zval

intrcpt 1.2476 0.9713 1.2846

`Severity of depression`Major depression -0.4036 1.0383 -0.3887

`Severity of depression`Mild to moderate depressive -0.6616 1.1919 -0.5551

pval ci.lb ci.ub

intrcpt 0.1989 -0.6560 3.1513

`Severity of depression`Major depression 0.6975 -2.4386 1.6315

`Severity of depression`Mild to moderate depressive 0.5788 -2.9976 1.6744

---

Signif. codes: 0 ‘***’ 0.001 ‘**’ 0.01 ‘*’ 0.05 ‘.’ 0.1 ‘ ’ 1

Warning message:

1 study with NAs omitted from model fitting.

> metareg(m1,~`Total Session`)

Mixed-Effects Model (k = 10; tau^2 estimator: REML)

tau^2 (estimated amount of residual heterogeneity): 0.4531 (SE = 0.4179)

tau (square root of estimated tau^2 value): 0.6731

I^2 (residual heterogeneity / unaccounted variability): 56.11%

H^2 (unaccounted variability / sampling variability): 2.28

R^2 (amount of heterogeneity accounted for): 0.00%

Test for Residual Heterogeneity:

QE(df = 8) = 19.4290, p-val = 0.0127

Test of Moderators (coefficient 2):

QM(df = 1) = 0.0024, p-val = 0.9612

Model Results:

estimate se zval pval ci.lb ci.ub

intrcpt 0.8508 0.5979 1.4230 0.1547 -0.3210 2.0225

`Total Session` -0.0014 0.0281 -0.0486 0.9612 -0.0564 0.0537

---

Signif. codes: 0 ‘***’ 0.001 ‘**’ 0.01 ‘*’ 0.05 ‘.’ 0.1 ‘ ’ 1

Warning message:

1 study with NAs omitted from model fitting.

> metareg(m1,~`Sample size`)

Mixed-Effects Model (k = 10; tau^2 estimator: REML)

tau^2 (estimated amount of residual heterogeneity): 0.2480 (SE = 0.3347)

tau (square root of estimated tau^2 value): 0.4980

I^2 (residual heterogeneity / unaccounted variability): 37.08%

H^2 (unaccounted variability / sampling variability): 1.59

R^2 (amount of heterogeneity accounted for): 36.07%

Test for Residual Heterogeneity:

QE(df = 8) = 12.2278, p-val = 0.1413

Test of Moderators (coefficient 2):

QM(df = 1) = 2.3998, p-val = 0.1214

Model Results:

estimate se zval pval ci.lb ci.ub

intrcpt 1.5492 0.5506 2.8135 0.0049 0.4700 2.6283 **

`Sample size` -0.0102 0.0066 -1.5491 0.1214 -0.0230 0.0027

---

Signif. codes: 0 ‘***’ 0.001 ‘**’ 0.01 ‘*’ 0.05 ‘.’ 0.1 ‘ ’ 1

Warning message:

1 study with NAs omitted from model fitting.

> metareg(m1,~`Age`)

Mixed-Effects Model (k = 10; tau^2 estimator: REML)

tau^2 (estimated amount of residual heterogeneity): 0.4593 (SE = 0.4260)

tau (square root of estimated tau^2 value): 0.6777

I^2 (residual heterogeneity / unaccounted variability): 56.26%

H^2 (unaccounted variability / sampling variability): 2.29

R^2 (amount of heterogeneity accounted for): 0.00%

Test for Residual Heterogeneity:

QE(df = 8) = 19.7488, p-val = 0.0113

Test of Moderators (coefficient 2):

QM(df = 1) = 0.0295, p-val = 0.8637

Model Results:

estimate se zval pval ci.lb ci.ub

intrcpt 0.4618 2.1433 0.2155 0.8294 -3.7389 4.6625

Age 0.0095 0.0552 0.1716 0.8637 -0.0986 0.1176

---

Signif. codes: 0 ‘***’ 0.001 ‘**’ 0.01 ‘*’ 0.05 ‘.’ 0.1 ‘ ’ 1

Warning message:

1 study with NAs omitted from model fitting.

> metareg(m1,~`Female rate`)

Mixed-Effects Model (k = 10; tau^2 estimator: REML)

tau^2 (estimated amount of residual heterogeneity): 0.4137 (SE = 0.3975)

tau (square root of estimated tau^2 value): 0.6432

I^2 (residual heterogeneity / unaccounted variability): 53.90%

H^2 (unaccounted variability / sampling variability): 2.17

R^2 (amount of heterogeneity accounted for): 0.00%

Test for Residual Heterogeneity:

QE(df = 8) = 18.1028, p-val = 0.0205

Test of Moderators (coefficient 2):

QM(df = 1) = 0.3580, p-val = 0.5496

Model Results:

estimate se zval pval ci.lb ci.ub

intrcpt 0.0017 1.3986 0.0012 0.9991 -2.7395 2.7428

`Female rate` 1.2669 2.1174 0.5983 0.5496 -2.8831 5.4169

---

Signif. codes: 0 ‘***’ 0.001 ‘**’ 0.01 ‘*’ 0.05 ‘.’ 0.1 ‘ ’ 1

Drop-out rate

data<-read_xlsx("D:\\regression data drop-out.xlsx")

> colnames(data)

[1] "study" "Baseline score" "Class of antidepressant"

[4] "Type of NIBS" "Severity of depression" "Total Session"

[7] "Sample size" "Age" "Female rate"

[10] "event1" "n1" "event2"

[13] "n2"

> table(data$`Baseline score`)

16.9 17.1 19.39 21.7 22.13 22.65 23.72 25 25.1 43.16

1 1 1 1 1 1 1 1 1 1

> table(data$`Class of antidepressant`)

New type of antidepressant SNRIs or NaSSAs SSRIs

1 1 7

SSRIs or SNRIs

1

> table(data$`Type of NIBS`)

rTMS tDCS

6 5

> table(data$`Severity of depression`)

Major depression Major depression

1 5

Mild to moderate depressive Moderate to severe depression

3 1

> table(data$`Total Session`)

10 20 24

4 2 1

> table(data$`Sample size`)

42 43 45 47 50 56 99 100 127 150

1 1 1 1 1 1 1 1 1 1

> table(data$`Age`)

29.42 30.75 32.06 34.06 37.6 38.31 40.1 47.7 49.5

1 1 1 1 1 1 1 2 1

> table(data$`Female rate`)

0.46 0.5 0.59 0.6 0.62 0.68 0.7 0.78 0.8

1 1 2 1 1 1 1 1 1

> m1 = metabin( event1,n1,event2,n2,sm='OR',

+ studlab = study,data,common=FALSE)

> metareg(m1,~`Baseline score`)

Mixed-Effects Model (k = 10; tau^2 estimator: REML)

tau^2 (estimated amount of residual heterogeneity): 0 (SE = 0.2535)

tau (square root of estimated tau^2 value): 0

I^2 (residual heterogeneity / unaccounted variability): 0.00%

H^2 (unaccounted variability / sampling variability): 1.00

R^2 (amount of heterogeneity accounted for): 0.00%

Test for Residual Heterogeneity:

QE(df = 8) = 6.5991, p-val = 0.5804

Test of Moderators (coefficient 2):

QM(df = 1) = 0.0007, p-val = 0.9785

Model Results:

estimate se zval pval ci.lb ci.ub

intrcpt -0.1461 1.1001 -0.1328 0.8943 -2.3022 2.0100

`Baseline score` -0.0012 0.0452 -0.0269 0.9785 -0.0897 0.0873

---

Signif. codes: 0 ‘***’ 0.001 ‘**’ 0.01 ‘*’ 0.05 ‘.’ 0.1 ‘ ’ 1

Warning message:

1 study with NAs omitted from model fitting.

> metareg(m1,~`Class of antidepressant`)

Mixed-Effects Model (k = 10; tau^2 estimator: REML)

tau^2 (estimated amount of residual heterogeneity): 0.1609 (SE = 0.6475)

tau (square root of estimated tau^2 value): 0.4011

I^2 (residual heterogeneity / unaccounted variability): 13.46%

H^2 (unaccounted variability / sampling variability): 1.16

R^2 (amount of heterogeneity accounted for): 0.00%

Test for Residual Heterogeneity:

QE(df = 6) = 6.3009, p-val = 0.3903

Test of Moderators (coefficients 2:4):

QM(df = 3) = 0.3393, p-val = 0.9525

Model Results:

estimate se zval pval ci.lb

intrcpt -0.2719 0.6593 -0.4125 0.6800 -1.5641

`Class of antidepressant`SNRIs or NaSSAs 0.1087 0.9039 0.1202 0.9043 -1.6629

`Class of antidepressant`SSRIs 0.2550 0.7765 0.3284 0.7426 -1.2668

`Class of antidepressant`SSRIs or SNRIs -0.4629 1.4619 -0.3166 0.7515 -3.3282

ci.ub

intrcpt 1.0203

`Class of antidepressant`SNRIs or NaSSAs 1.8802

`Class of antidepressant`SSRIs 1.7768

`Class of antidepressant`SSRIs or SNRIs 2.4024

---

Signif. codes: 0 ‘***’ 0.001 ‘**’ 0.01 ‘*’ 0.05 ‘.’ 0.1 ‘ ’ 1

Warning message:

1 study with NAs omitted from model fitting.

> metareg(m1,~`Type of NIBS`)

Mixed-Effects Model (k = 11; tau^2 estimator: REML)

tau^2 (estimated amount of residual heterogeneity): 0 (SE = 0.2448)

tau (square root of estimated tau^2 value): 0

I^2 (residual heterogeneity / unaccounted variability): 0.00%

H^2 (unaccounted variability / sampling variability): 1.00

R^2 (amount of heterogeneity accounted for): 0.00%

Test for Residual Heterogeneity:

QE(df = 9) = 6.9418, p-val = 0.6432

Test of Moderators (coefficient 2):

QM(df = 1) = 0.7155, p-val = 0.3976

Model Results:

estimate se zval pval ci.lb ci.ub

intrcpt -0.2275 0.2945 -0.7725 0.4398 -0.8046 0.3497

`Type of NIBS`tDCS 0.3902 0.4613 0.8459 0.3976 -0.5139 1.2943

---

Signif. codes: 0 ‘***’ 0.001 ‘**’ 0.01 ‘*’ 0.05 ‘.’ 0.1 ‘ ’ 1

> metareg(m1,~`Severity of depression`)

Mixed-Effects Model (k = 10; tau^2 estimator: REML)

tau^2 (estimated amount of residual heterogeneity): 0.0744 (SE = 0.5915)

tau (square root of estimated tau^2 value): 0.2728

I^2 (residual heterogeneity / unaccounted variability): 6.75%

H^2 (unaccounted variability / sampling variability): 1.07

R^2 (amount of heterogeneity accounted for): 0.00%

Test for Residual Heterogeneity:

QE(df = 6) = 5.4322, p-val = 0.4897

Test of Moderators (coefficients 2:4):

QM(df = 3) = 1.1241, p-val = 0.7713

Model Results:

estimate se zval

intrcpt 0.3589 1.0004 0.3588

`Severity of depression`Major depression -0.8195 1.0890 -0.7526

`Severity of depression`Mild to moderate depressive -0.2027 1.1285 -0.1796

`Severity of depression`Moderate to severe depression -0.5222 1.1387 -0.4586

pval ci.lb ci.ub

intrcpt 0.7197 -1.6018 2.3197

`Severity of depression`Major depression 0.4517 -2.9539 1.3148

`Severity of depression`Mild to moderate depressive 0.8575 -2.4144 2.0091

`Severity of depression`Moderate to severe depression 0.6465 -2.7541 1.7096

---

Signif. codes: 0 ‘***’ 0.001 ‘**’ 0.01 ‘*’ 0.05 ‘.’ 0.1 ‘ ’ 1

Warning message:

1 study with NAs omitted from model fitting.

> metareg(m1,~`Total Session`)

Mixed-Effects Model (k = 7; tau^2 estimator: REML)

tau^2 (estimated amount of residual heterogeneity): 0 (SE = 0.6981)

tau (square root of estimated tau^2 value): 0

I^2 (residual heterogeneity / unaccounted variability): 0.00%

H^2 (unaccounted variability / sampling variability): 1.00

R^2 (amount of heterogeneity accounted for): 100.00%

Test for Residual Heterogeneity:

QE(df = 5) = 2.0341, p-val = 0.8444

Test of Moderators (coefficient 2):

QM(df = 1) = 3.7383, p-val = 0.0532

Model Results:

estimate se zval pval ci.lb ci.ub

intrcpt 1.9496 1.0937 1.7825 0.0747 -0.1941 4.0932 .

`Total Session` -0.1146 0.0593 -1.9335 0.0532 -0.2309 0.0016 .

---

Signif. codes: 0 ‘***’ 0.001 ‘**’ 0.01 ‘*’ 0.05 ‘.’ 0.1 ‘ ’ 1

Warning message:

4 studies with NAs omitted from model fitting.

> metareg(m1,~`Sample size`)

Mixed-Effects Model (k = 10; tau^2 estimator: REML)

tau^2 (estimated amount of residual heterogeneity): 0 (SE = 0.2674)

tau (square root of estimated tau^2 value): 0

I^2 (residual heterogeneity / unaccounted variability): 0.00%

H^2 (unaccounted variability / sampling variability): 1.00

R^2 (amount of heterogeneity accounted for): 0.00%

Test for Residual Heterogeneity:

QE(df = 8) = 4.1227, p-val = 0.8459

Test of Moderators (coefficient 2):

QM(df = 1) = 2.4771, p-val = 0.1155

Model Results:

estimate se zval pval ci.lb ci.ub

intrcpt 0.8728 0.7108 1.2279 0.2195 -0.5204 2.2660

`Sample size` -0.0105 0.0066 -1.5739 0.1155 -0.0235 0.0026

---

Signif. codes: 0 ‘***’ 0.001 ‘**’ 0.01 ‘*’ 0.05 ‘.’ 0.1 ‘ ’ 1

Warning message:

1 study with NAs omitted from model fitting.

> metareg(m1,~`Age`)

Mixed-Effects Model (k = 10; tau^2 estimator: REML)

tau^2 (estimated amount of residual heterogeneity): 0 (SE = 0.3204)

tau (square root of estimated tau^2 value): 0

I^2 (residual heterogeneity / unaccounted variability): 0.00%

H^2 (unaccounted variability / sampling variability): 1.00

R^2 (amount of heterogeneity accounted for): 0.00%

Test for Residual Heterogeneity:

QE(df = 8) = 5.8604, p-val = 0.6629

Test of Moderators (coefficient 2):

QM(df = 1) = 0.7394, p-val = 0.3898

Model Results:

estimate se zval pval ci.lb ci.ub

intrcpt 0.9712 1.3560 0.7162 0.4738 -1.6865 3.6289

Age -0.0286 0.0333 -0.8599 0.3898 -0.0939 0.0366

---

Signif. codes: 0 ‘***’ 0.001 ‘**’ 0.01 ‘*’ 0.05 ‘.’ 0.1 ‘ ’ 1

Warning message:

1 study with NAs omitted from model fitting.

> metareg(m1,~`Female rate`)

Mixed-Effects Model (k = 10; tau^2 estimator: REML)

tau^2 (estimated amount of residual heterogeneity): 0 (SE = 0.2506)

tau (square root of estimated tau^2 value): 0

I^2 (residual heterogeneity / unaccounted variability): 0.00%

H^2 (unaccounted variability / sampling variability): 1.00

R^2 (amount of heterogeneity accounted for): 0.00%

Test for Residual Heterogeneity:

QE(df = 8) = 6.5966, p-val = 0.5807

Test of Moderators (coefficient 2):

QM(df = 1) = 0.0032, p-val = 0.9550

Model Results:

estimate se zval pval ci.lb ci.ub

intrcpt -0.0589 2.0712 -0.0284 0.9773 -4.1184 4.0005

`Female rate` -0.1931 3.4225 -0.0564 0.9550 -6.9010 6.5148

---

Signif. codes: 0 ‘***’ 0.001 ‘**’ 0.01 ‘*’ 0.05 ‘.’ 0.1 ‘ ’ 1

Warning message:

1 study with NAs omitted from model fitting.
