# Supplementary material for: Efficacy of non-invasive brain stimulation combined with antidepressant medications for depression: a systematic review and meta-analysis of randomized controlled trials
Source: Syst Rev. 2024 Mar 20;13:92. doi: 10.1186/s13643-024-02480-w (PMC10953221; doi:10.1186/s13643-024-02480-w)
Supplement: Supplementary file 4 — Supplementary Materials file 4. [file 13643_2024_2480_MOESM4_ESM.doc]

**Sensitivity Analysis**


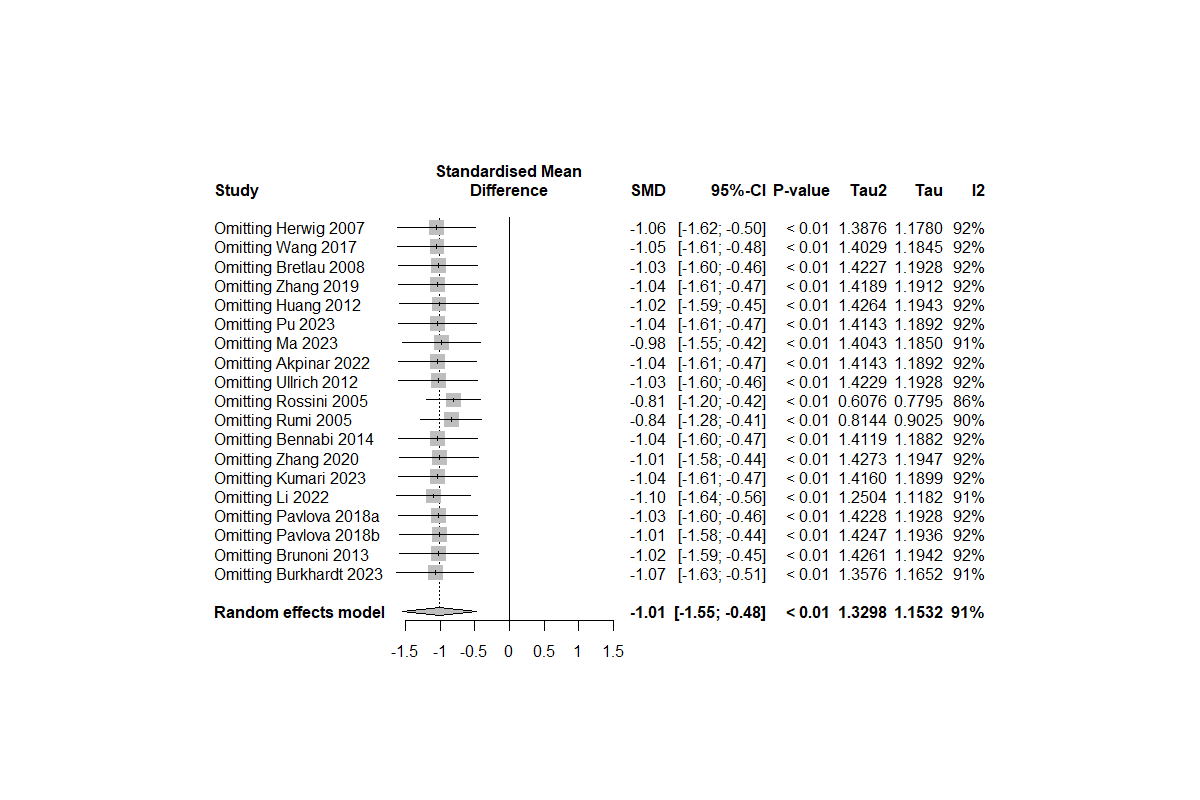


Fig. S1 Sensitivity analysis of depression scores


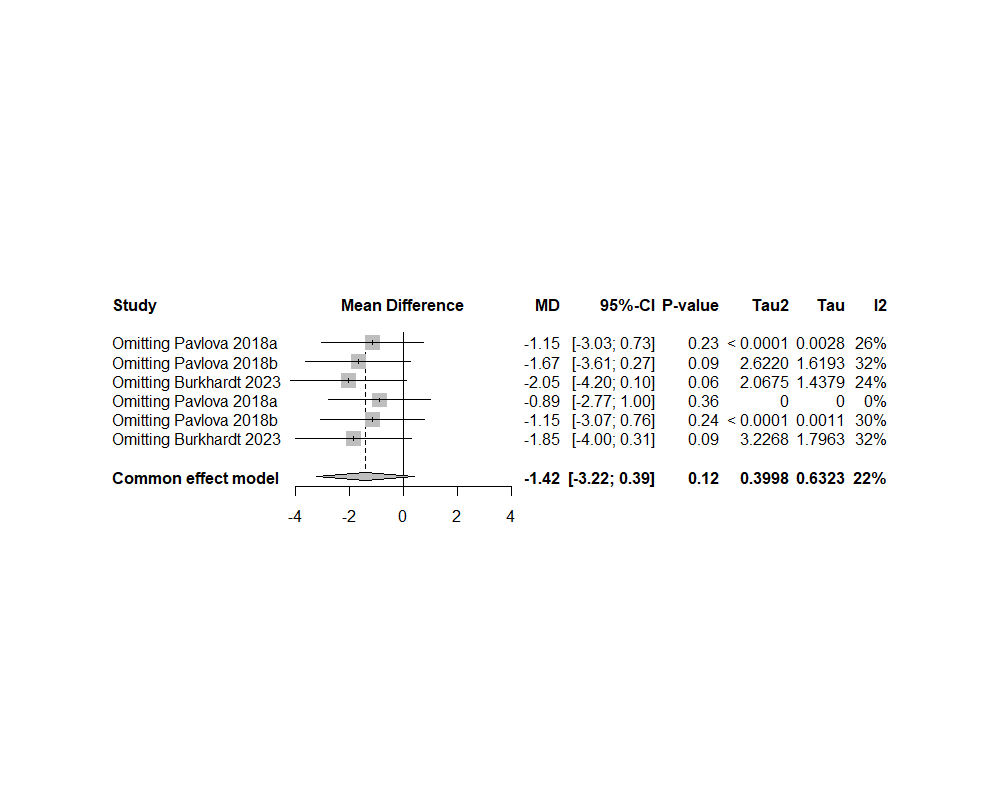


Fig. S2 Sensitivity analysis of anxiety score


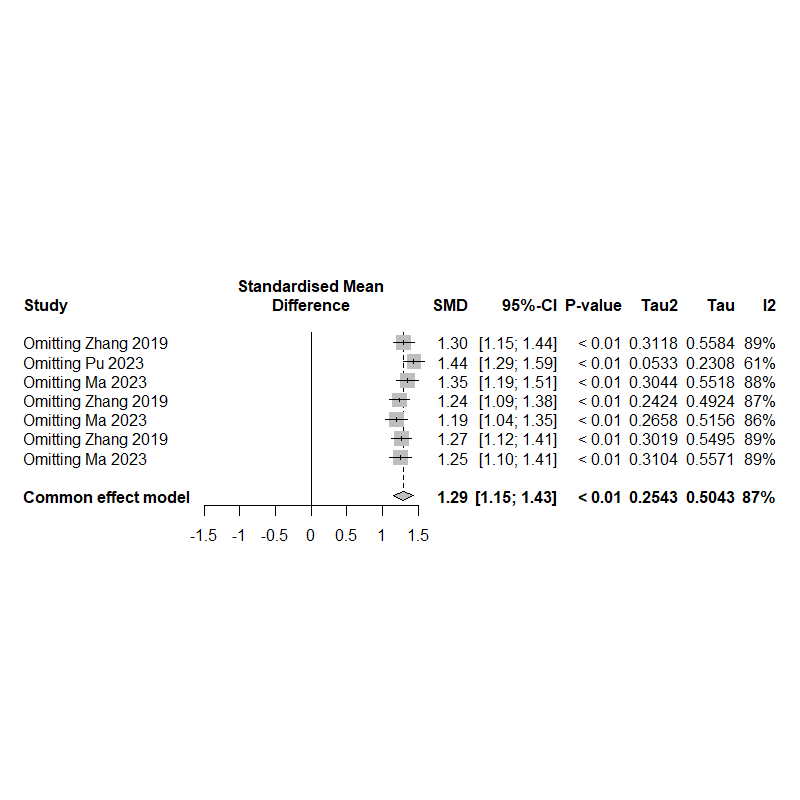


Fig. S3 Sensitivity analysis of levels of neurotransmitters


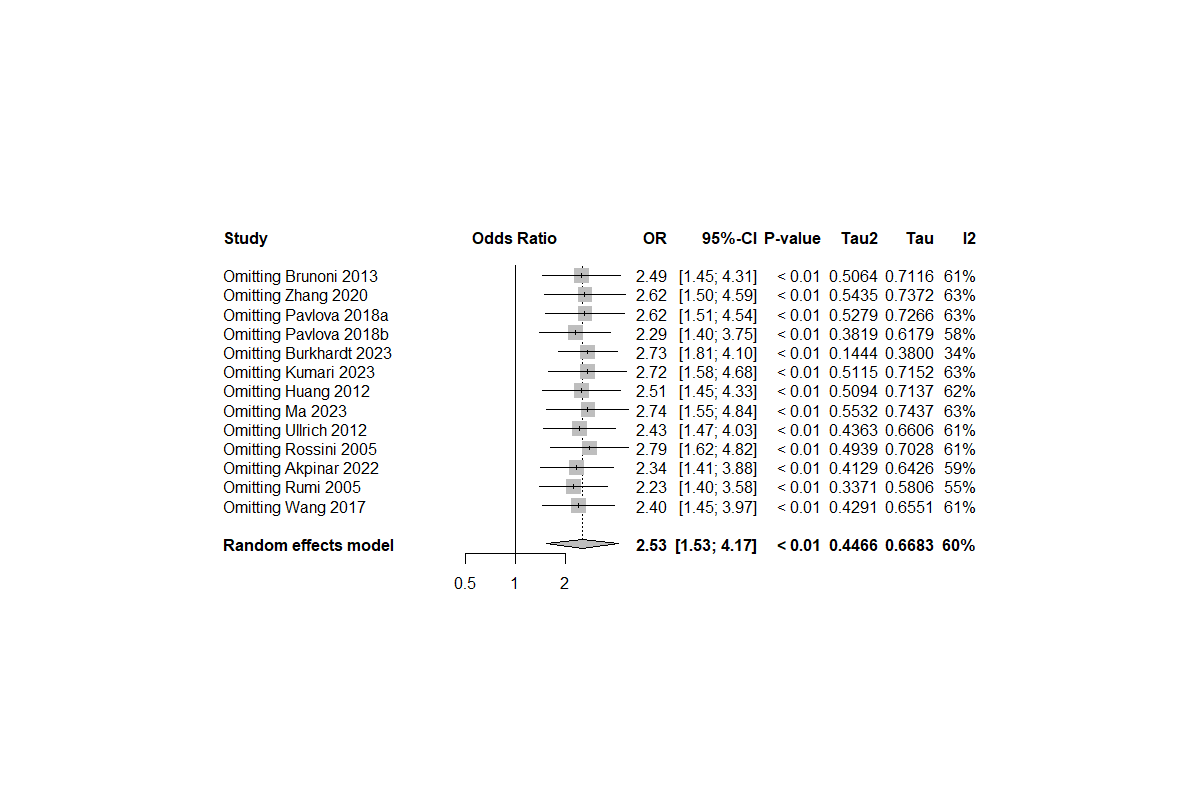


Fig. S4 Sensitivity analysis of response rate


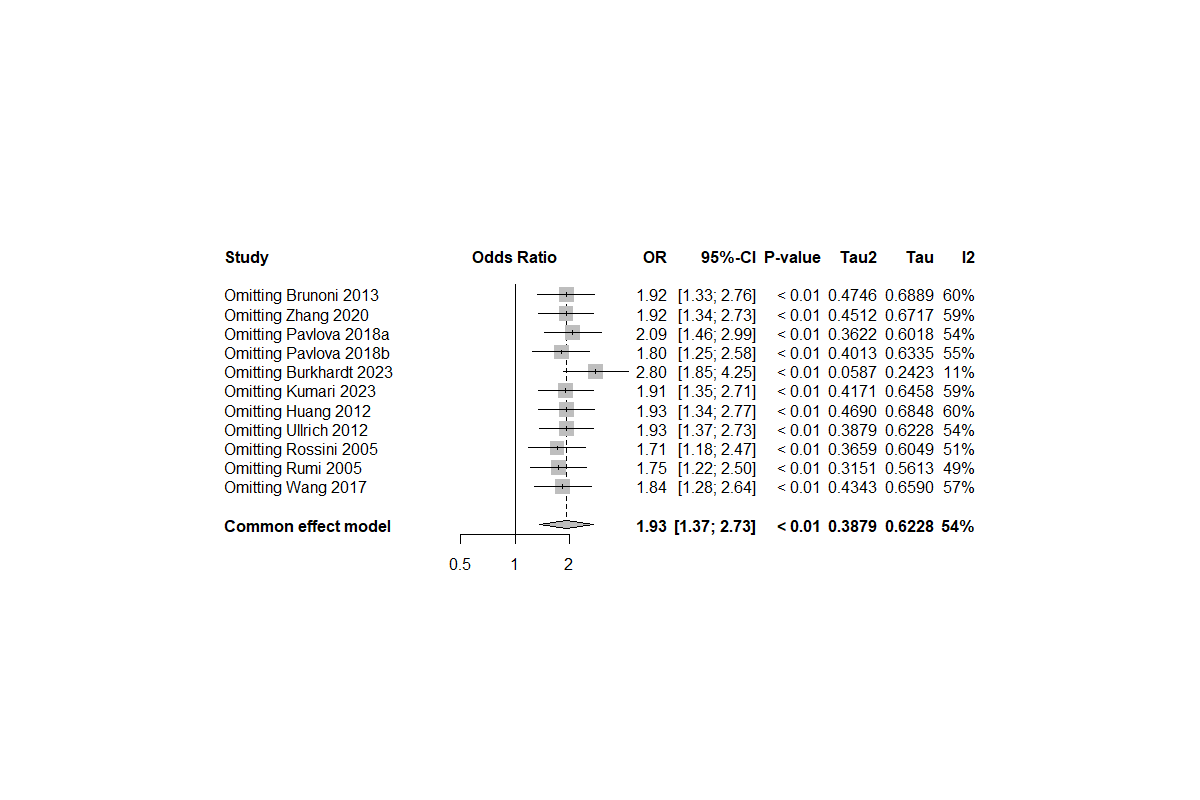


Fig. S5 Sensitivity analysis of remission rate


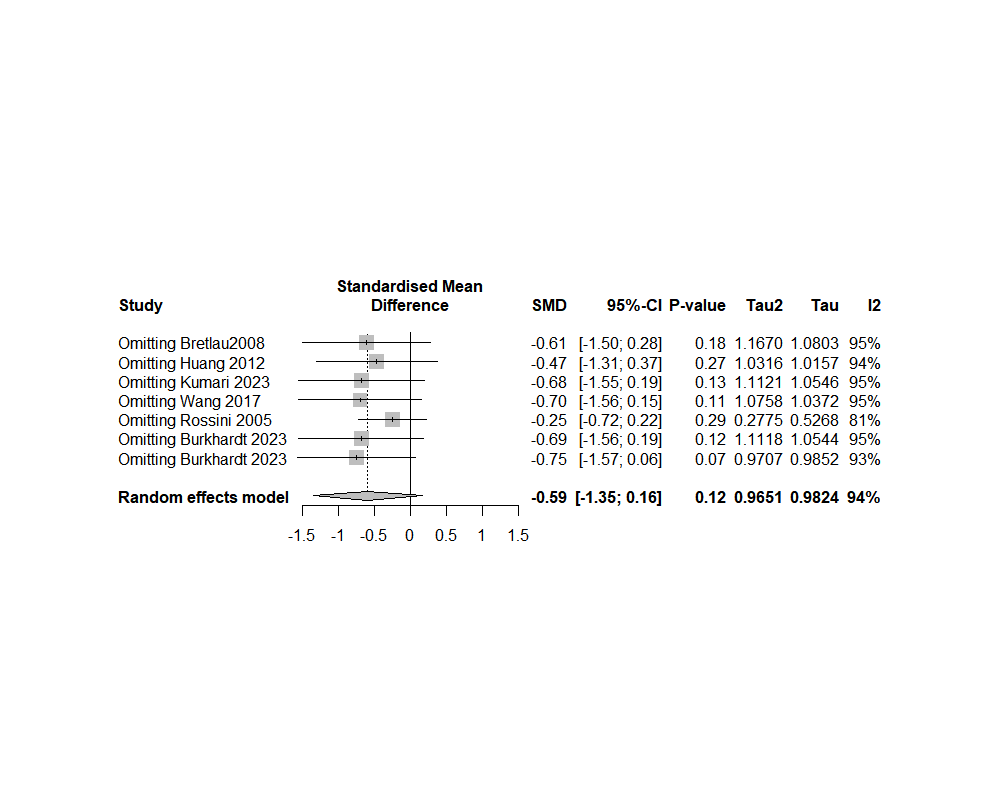


Fig. S6 Sensitivity analysis of follow-up time


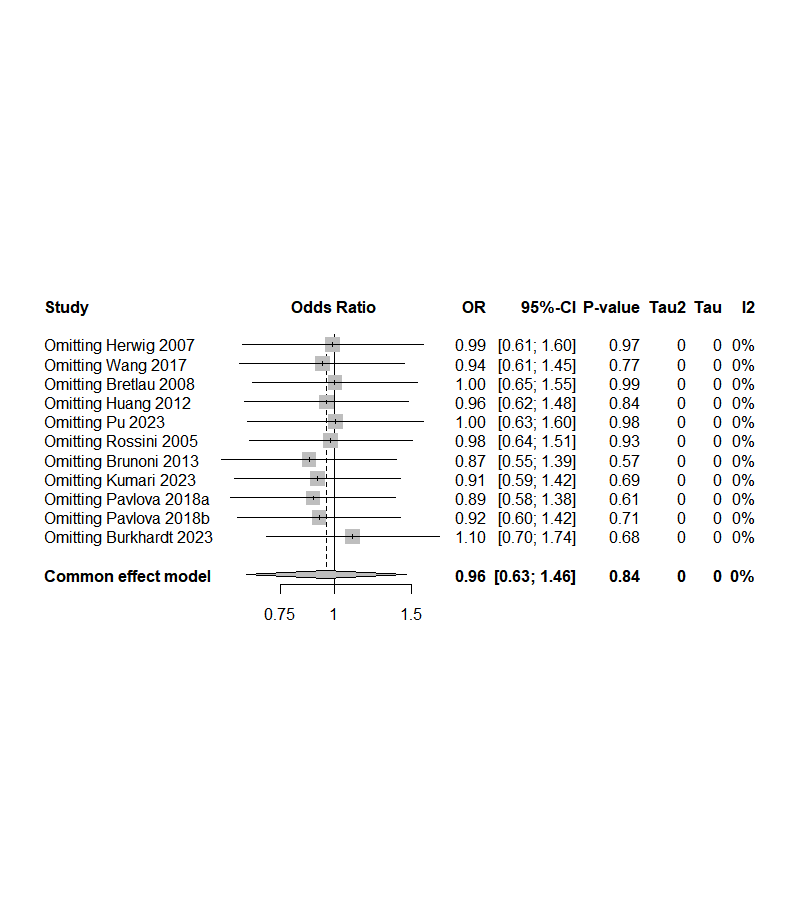


Fig. S7 Sensitivity analysis of droup-out rate
