# Supplementary material for: Efficacy of non-invasive brain stimulation combined with antidepressant medications for depression: a systematic review and meta-analysis of randomized controlled trials
Source: Syst Rev. 2024 Mar 20;13:92. doi: 10.1186/s13643-024-02480-w (PMC10953221; doi:10.1186/s13643-024-02480-w)
Supplement: Supplementary file 5 — Supplementary Materials file 5. [file 13643_2024_2480_MOESM5_ESM.doc]

# Publication bias

## Figure S8 : Funnel plots (a) depression score; (b) response rate; (c) remission rate; (d) drop out rate

(a) depression score (b) response rate


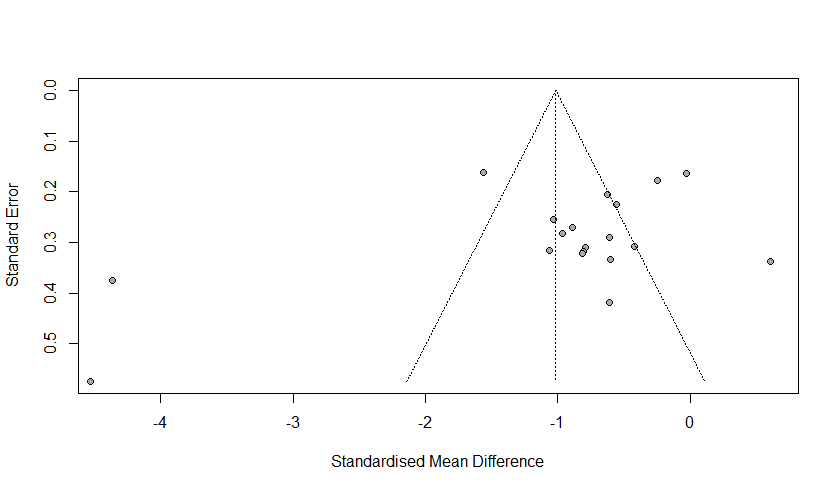

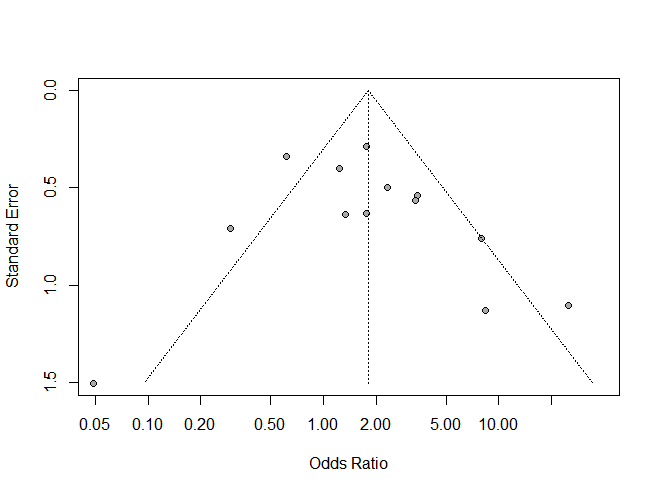


(c) remission rate (d) drop out rate


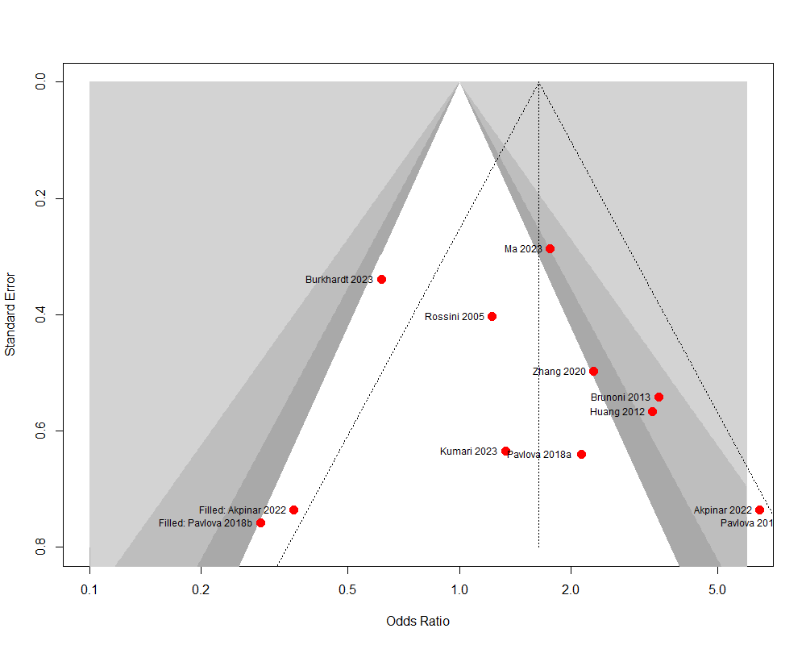

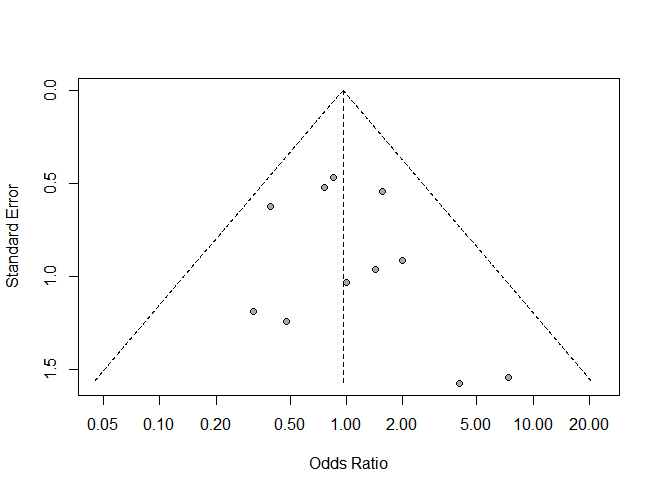


**Table S5. Egger's test for each outcome**

| **depression score** | | | **response rate** | | | **remission rate** | | | **drop out rate** | | |
| --- | --- | --- | --- | --- | --- | --- | --- | --- | --- | --- | --- |
| t | df | *p* | t | df | *p* | t | df | *p* | t | df | *p* |
| -0.18 | 17 | 0.32 | 3.34 | 11 | 0.00 | 1.54 | 8 | 0.16 | 1.24 | 9 | 0.24 |
