# Supplementary material for: Efficacy of non-invasive brain stimulation combined with antidepressant medications for depression: a systematic review and meta-analysis of randomized controlled trials
Source: Syst Rev. 2024 Mar 20;13:92. doi: 10.1186/s13643-024-02480-w (PMC10953221; doi:10.1186/s13643-024-02480-w)
Supplement: Supplementary file 6 — Supplementary Materials file 6. [file 13643_2024_2480_MOESM6_ESM.doc]

**Influence factors**

Table S1: Univariable meta-regression for remission rate

| **Variable** | **Remission rate** | | |
| --- | --- | --- | --- |
|  | Coef (B) | 95% CL | *P* |
| *Clinical characteristics* |  |  |  |
| Type of NIBS | -0.96 | -1.93 to 0.02 | 0.05 |
| Total Session | -0.00 | -0.05 to 0.05 | 0.96 |
| Severity of depression |  |  |  |
| Major depression | -0.40 | -2.43 to 1.63 | 0.69 |
| Mild to moderate depressive | -0.66 | -2.99 to 1.67 | 0.57 |
| Baseline score ^a^  Class of antidepressant | 0.04 | -0.03 to 0.11 | 0.29 |
| SSRIs | -1.58 | -3.43 to 0.26 | 0.09 |
| SSRIs and SNRIs | -0.53 | -2.82 to 1.76 | 0.65 |
| Demographics |  |  |  |
| Sample size | -0.01 | -0.02 to 0.00 | 0.12 |
| Age | 0.00 | -0.09 to 0.11 | 0.86 |
| Female rate | 1.26 | -2.88 to 5.14 | 0.54 |

Note: SSRIs :serotonin-norepinephrine reuptake inhibitors; SNRIs: serotonin-norepinephrine reuptake inhibitors, ^a^Baseline score was calculated by the weighted arithmetic mean of depression scores of NIBS and control groups groups. Each variable was analysed separately in a meta-regression model, Coef(B) represents the regression coeffcient of each linear regression, representing the slope of each model, 95% CI is the 95% confidenceinterval of the beta coeffcient values.

Table S2: Univariable meta-regression for depression score

| **Variable** | **depression score** | | |
| --- | --- | --- | --- |
|  | Coef (B) | 95% CL | *p* |
| Clinical characteristics |  |  |  |
| Type of NIBS | 0.79 | -0.26 to 1.84 | 0.14 |
| Total session | 0.00 | -0.05 to 0.05 | 0.92 |
| Severity of depression |  |  |  |
| Major depression | -0.63 | -2.55 to 1.27 | 0.51 |
| Mild to moderate depressive | -0.15 | -2.32 to 2.01 | 0.89 |
| Moderate to severe depression | 0.36 | -2.67 to 3.38 | 0.82 |
| Baseline score ^a^  Class of antidepressant | -0.03 | -0.12 to 0.06 | 0.50 |
| SNRIs | -0.35 | -2.60 to 189 | 0.76 |
| SNRIs and NaSSAs | 0.30 | -2.51 to 312 | 0.83 |
| SSRIs | -0.24 | -2.36 to 1.89 | 0.83 |
| SSRIs and SNRIs | -1.30 | -3.78 to 1.18 | 0.31 |
| TCAs | -3.97 | -6.99 to -0.96 | 0.01* |
| Demographics |  |  |  |
| Sample size | 0.00 | -1.01 to 0.01 | 0.51 |
| Age | 0.00 | -0.06 to 0.06 | 0.97 |
| Female rate | -4.45 | -8.37 to -0.6 | 0.02* |

Note: *p<0.05; SSRIs :serotonin-norepinephrine reuptake inhibitors; SNRIs: serotonin-norepinephrine reuptake inhibitors, NaSSAs :Noradrenergic and specific serotonergic antidepressants; TCAs: Tricyclic Antidepressive Agents aBaseline score was calculated by the weighted arithmetic mean of depression scores of NIBS and control groups groups. Each variable was analysed separately in a meta-regression model, Coef(B) represents the regression coeffcient of each linear regression, representing the slope of each model, 95% CI is the 95% confidenceinterval of the beta coeffcient values.

Table S3: Univariable meta-regression for response rate

| **Variable** | **Response rate** | | |
| --- | --- | --- | --- |
|  | Coef (B) | 95% CL | *P* |
| *Clinical characteristics* |  |  |  |
| Type of NIBS | -0.45 | -1.50 to 0.58 | 0.39 |
| Total Session | -0.02 | -0.06 to 0.02 | 0.36 |
| Severity of depression |  |  |  |
| Major depression | -1.27 | -3.90 to 1.34 | 0.34 |
| Mild to moderate depressive | -0.76 | -3.65 to 2.12 | 0.60 |
| Baseline score ^a^  Class of antidepressant | 0.04 | -0.04 to 0.13 | 0.38 |
| SNRIs | 1.32 | -2.13 to 4.78 | 0.45 |
| SSRIs | -0.12 | -1.43 to 1.19 | 0.85 |
| SSRIs and SNRIs | -0.81 | -2.74 to 1.10 | 0.40 |
| TCAs | 2.18 | 0.59 to 4.97 | 0.12 |
| Demographics |  |  |  |
| Sample size | -0.09 | -0.01 to -0.00 | 0.01* |
| Age | -0.00 | -0.09 to 0.07 | 0.88 |
| Female rate | 1.57 | -2.25 to 5.39 | 0.42 |

Note: *p<0.05; SSRIs :serotonin-norepinephrine reuptake inhibitors; SNRIs: serotonin-norepinephrine reuptake inhibitors, NaSSAs :Noradrenergic and specific serotonergic antidepressants; TCAs: Tricyclic Antidepressive Agents aBaseline score was calculated by the weighted arithmetic mean of depression scores of NIBS and control groups groups. Each variable was analysed separately in a meta-regression model, Coef(B) represents the regression coeffcient of each linear regression, representing the slope of each model, 95% CI is the 95% confidenceinterval of the beta coeffcient values.

Table S4: Univariable meta-regression for Drop-out rate

| **Variable** | **Drop-out rate** | | |
| --- | --- | --- | --- |
|  | Coef (B) | 95% CL | *P* |
| *Clinical characteristics* |  |  |  |
| Type of NIBS | 0.39 | -1.51 to 1.29 | 0.39 |
| Total Session | -0.11 | -0.230 to 0.00 | 0.05 |
| Severity of depression |  |  |  |
| Major depression | -0.81 | -2.95 to 1.31 | 0.45 |
| Mild to moderate depressive | -0.20 | -2.41 to 2.00 | 0.85 |
| Moderate to severe depression | -0.52 | -2.75 to 1.7 | 0.64 |
| Baseline score ^a^  Class of antidepressant | -0.00 | -0.08 to 0.08 | 0.97 |
| SSRIs | 0.25 | -1.26 to 1.77 | 0.74 |
| SSRIs and SNRIs | 0.11 | -3.32 to 2.24 | 0.75 |
| SNRIs and NaSSAs | -0.46 | -1.66 to 1.88 | 0.90 |
| Demographics |  |  |  |
| Sample size | --0.01 | -0.02 to -0.00 | 0.11 |
| Age | -0.02 | -0.09 to 0.03 | 0.38 |
| Female rate | -0.19 | -6.90 to 6.51 | 0.95 |

Note: *p<0.05; SSRIs :serotonin-norepinephrine reuptake inhibitors; SNRIs: serotonin-norepinephrine reuptake inhibitors, NaSSAs :Noradrenergic and specific serotonergic antidepressants; TCAs: Tricyclic Antidepressive Agents aBaseline score was calculated by the weighted arithmetic mean of depression scores of NIBS and control groups groups. Each variable was analysed separately in a meta-regression model, Coef(B) represents the regression coeffcient of each linear regression, representing the slope of each model, 95% CI is the 95% confidenceinterval of the beta coeffcient values.
